# Relative sea level projections constrained by historical trends at tide gauge sites

**DOI:** 10.1126/sciadv.ado4506

**Published:** 2025-10-01

**Authors:** Mahé Perrette, Matthias Mengel

**Affiliations:** Potsdam Institute for Climate Impact Research, 14473 Potsdam, Germany.

## Abstract

Assessing the impacts of future relative sea level rise requires projections consistent with historical observations. However, existing projections often do not align with past data, complicating adaptation planning, impact assessments, and communication. We present a spatial Bayesian model that generates local projections at tide gauge sites from historical records. The model integrates tide gauges, GPS, and satellite altimetry with past and future constraints on mountain glaciers, polar ice sheets, thermal expansion, ocean circulation, land water storage, and glacial history. By separating unforced ocean variability from long-term trends, we provide posterior estimates of sea level change and vertical land motion. The inclusion of local constraints reduces uncertainty in near-term local projections while producing global median projections and uncertainty ranges similar to those in the Sixth Assessment Report (AR6) of the Intergovernmental Panel on Climate Change (IPCC). The model enables projections of local relative sea level rise for any given global temperature trajectory, illustrated with three IPCC AR6 Working Group III pathways.

## Teaser

Local sea level rise projections consistent with historical data improve coastal impact assessment and adaptation planning.

## INTRODUCTION

Sea level rise is a major consequence of climate change (*1*) and has profound impacts on coastal regions, negatively affecting humans and ecosystems alike (*2*, *3*). Much attention has been given to global sea level change and its relation to climate change, but threats to the coasts depend on the relative sea level rise in the regions at risk, which emerges as a complex combination of climatic and nonclimatic processes (*4*, *5*). The impact-relevant metric of relative sea level rise as measured by tide gauges is the combination of rising ocean waters and the subsidence (or uplift) of the land (*6*). To quantify related losses and damages (*7*), it is necessary to estimate the effect of greenhouse gas emitters on this impact-relevant metric, which so far has not been done on a global scale. To adapt to future relative sea level rise (*8*–*10*), the best available knowledge on the local contributions should be used to not only minimize risk, but also avoid maladaptation and overinvestment.

Scholars have combined multiple historical datasets and process knowledge into globally consistent reconstructions of past regional sea level rise (*11*–*14*). It is possible to partition past sea level rise into its major global contributors since the early 20th century (*13*), being thermal expansion, glacier and ice sheet mass loss, and land water storage changes. Future regional sea level rise projections are now abundant (*15*–*17*) with a synthesis of the current state of knowledge in the Sixth Assessment Report (AR6) of the Intergovernmental Panel on Climate Change (IPCC) (*1*). Kopp *et al*. (*18*) present projections that translate process-based estimates and expert elicitation of global sea level rise to the local scale at tide gauge locations. Other studies (*19*–*21*) use similar frameworks to project relative sea level rise probabilistically by combining uncertain contributions through Monte Carlo sampling. So far, all global projection studies for local relative sea level share a common limitation—the data and knowledge of global and local scale is not combined into one framework, so uncertainty propagates from global climate change to the local projections, but the local observational data inform neither the projections at the specific or nearby locations, nor the global contributions to reduce overall uncertainty. This one-directional information flow from global to local leads to large uncertainty ranges at the local scale, which is often in contrast to the smaller uncertainty ranges of trends directly estimated from local data. Existing frameworks incorporating local tide gauge data are limited to the nonclimatic background contribution to local relative sea level change (*1*, *18*). In the IPCC AR6 projections, the individual contributions to sea level rise and their uncertainties are computed separately from a common global temperature pathway and then added, assuming independent errors (*1*).

Missing local constraints are a problem for planners as they do not only rely on sea level projections derived from large-scale processes, but also on local knowledge that is often inferred from historical data and experience (*22*–*25*). For impact assessments, the missing integration is problematic because vertical land motion (VLM) can be an important driver of sea level impacts (*10*, *26*, *27*) but is not necessarily related to anthropogenic climate change, and this differentiation is a critical aspect for the loss and damage debate.

Moreover, the fixed scenario setup in many of the available approaches hinders analyses that rely on nonstandard or new scenarios, e.g., on attribution and sea level commitment (*28*, *29*), the differential impacts from sea level rise (*30*), or the role of mitigation policies (*31*). The fixed scenario setup also hinders sea level projections that are consistent across IPCC working groups, an important task that has already been achieved for aggregated atmospheric climate variables through reduced-complexity climate models (*32*).

Bayesian spatial methods make it possible to combine available information consistently into one model including their uncertainties, even if they originate from different domains or cover different time periods (*33*, *34*). So far, studies using such methods advanced the field of sea level reconstruction, working on the recent (*35*, *36*) and extended historical periods (*37*). See (*38*) for a review of uncertainty quantification approaches in sea level reconstructions.

While probabilistic frameworks are commonly used for sea level projections (*19*–*21*), very few studies use a Bayesian approach for uncertainty quantification (*39*, *40*). To date, there are no studies that use a Bayesian approach to combine spatial historical data with models relating local sea level rise to global climate change. In our case, the spatial historical data include tide gauge records (*41*, *42*), Global Positioning System (GPS) measurements (*43*), and satellite altimetry data (*44*). As we aim to produce sea level projections from climate pathways, we go beyond these historical data, which are typically used in sea level reconstruction studies. We also include knowledge on the contributors to global sea level change for both past and future (*1*, *13*), their relation to climate change as encoded in empirical relations (*45*–*47*), and their regionally varying effect on relative sea level change (*48*, *49*). We present a Bayesian spatial model that integrates these data to estimate annual mean sea level change from the year 1900 to the year 2100. The model enables projections of local relative sea level rise at tide gauge sites for any given global mean temperature trajectory, and we illustrate this with pathways from the IPCC AR6 Working Group III (WG3) (*31*).

### Bayesian spatial model for mean sea level change

The model consists of a set of deterministic mathematical equations that link global mean temperature *T* with the local mean sea level (*s* = 1) or coastal height (*s* = 2) change $y^{s}$ relative to the Earth’s center of mass, at any time *t* and location *x*

$$\begin{array}{c} y^{s}\;\left(x,t\right)=\sum_{k=1}^{24}y_{k}\left[t,T\left(t\right),\alpha_{k},\varepsilon_{k}\left(t\right)\right]\;f_{k}^{s}\left(x,\beta_{k}\right)+t\beta_{25}\left(x\right)\;\delta_{s,2} \end{array}$$ (1)

where $y_{k}$ are space-invariant source contributions (generally, the time-varying global mean or mass source) and $f_{k}^{s}$ are time-invariant fields or fingerprints (*1*, *13*, *18*–*21*, *49*), with $\alpha_{k}$ and $\beta_{k}$ being model parameters. The source contributions have a climate-driven term parameterized in relation to global mean temperature except for land water, and a noise term $\varepsilon_{k}$ to account for unforced natural variability in the rate of the source contributions. The rate of residual VLM $\beta_{25}\left(x\right)$ captures additional, local processes and is constant in time in the model (*13*). A more detailed version and explanation of Eq. 1 is given in Materials and Methods (Eq. 2). The source contributions include mountain glaciers in 19 glaciated regions (*k* = 1–19), the Greenland and Antarctic ice sheets (*k* = 20, 21), land water storage changes (*k* = 22), ocean sterodynamic changes (*k* = 23), and glacial isostatic adjustment [(GIA) (*k* = 24)]. Each source contribution is associated with two fingerprints, one describing geocentric sea level (GSL; *s* = 1), as observed from space and relative to the center of mass of the Earth, and the other describing the vertical movement of the coast (*s* = 2). Both components add to relative sea level rise. The Kronecker symbol $\delta_{s,2}$ indicates that the residual VLM does not affect GSL.

The sea level fingerprints $f_{s}^{k}$ are formed by the gravitational effect of the ice and land water mass changes, the associated land uplift due to unloading, and their influence on the Earth’s rotation (*6*). We separate the effect of ocean circulation and density changes, also called sterodynamic sea level change, into climate-driven changes that are sustained in time and into transient, unforced interannual variability. An example of climate-driven change is the enhanced sea level rise along the Northeast American coast, associated with a slowdown of the meridional overturning circulation(*50*). Following (*21*, *48*), we extend the fingerprint concept to climate-driven sterodynamic sea level changes through a formulation that links global mean thermal expansion with the local sterodynamic sea level change. Unforced ocean variability is treated separately in the local likelihood function, which we describe below.

We follow a Bayesian framework to estimate the model parameters and resulting sea level time series between 1900 and 2100 according to a range of global and local observations. The model parameters $\alpha_{k}$ and $\beta_{k}$ are randomly sampled from a prior distribution and remain constant in time for the full period, while the noise $\varepsilon_{k}\left(t\right)$ is sampled for each year. We define prior parameter distributions according to published datasets when possible or we choose a distribution that is sufficiently broad to have little impact on the posterior (see Materials and Methods and Table 1). Additional observational constraints enter the model through the likelihood function, which expresses the goodness of fit of each model sample with respect to the observations. The combination of prior parameter distributions and likelihood function determines the posterior distribution of the model samples. Each model sample is a full set of global and local sea level time series for 578 tide gauge locations, including their contributions as described by Eq. 1.

**Table 1.** Uncertain model parameters and their prior distribution. Noise hyperparameters, an estimate of the variance, are marked with asterisk (*) to differentiate them from noise samples. Greenland and Antarctica only include the ice sheets and not the peripheral mountain glaciers. The peripheral mountain glaciers are included as mountain glaciers (*k* = 5 and *k* = 19, respectively). PC refers to “principal components.” The timescales in the noise distribution and the length scale in the VLM residual prior refer to an exponential distribution corr(x,y)=exp−∣x−y∣/λ in time or space.

Variable	Source	Type	Climate	Distribution	Units	Shape	Dataset
${\tilde{a}}_{1-19}$	Glaciers	Trend	Yes	Exp(0.3)	1/K	19	
${\tilde{b}}_{1-19}$		Trend	Yes	N(1, 2)		19	
$V_{2000,1-19}$		Trend	Yes	N (from data)	mm	19	AR6 table 9.SM.2 (*1*); Farinotti *et al*. (*85*)
$u_{1901,2015}$		Trend	No	U(17, 48)	mm		Parkes and Marzeion (*84*)
$\varepsilon_{1-19}$		Noise	No	N(0, $\sigma_{k,\text{obs}}$ , 5 years)	mm/year	19, 200	Malles and Marzeion (*51*)
$a_{20}$	Greenland	Trend	Yes		mm/year per K		
$b_{20}$		Trend	Yes		mm/year		AR6 table 9.S (*1*)
$q_{20}$		Trend	Yes		mm/year^2^ per K		
$\sigma_{20}$		Noise*	No		mm/year		
$\varepsilon_{20}$		Noise	No	N(0, $\sigma_{20,}$ , 5 years)	mm/year	200	
$b_{21}$	Antarctica	Trend	Yes		mm/year		AR6 table 9.S (*1*)
$a_{21}$		Trend	Yes		mm/year per K		
$q_{21}$		Trend	Yes		mm/year^2^ per K		
$\varepsilon_{21}$		Noise	No		mm/year	200	Frederikse *et al*. (*13*)
$\beta_{22}$	Land water	Trend + Noise	No		mm/year	200	Frederikse *et al*. (*13*), AR6 (*1*)
$a_{23}$	Sterodynamic	Trend	Yes		mm/year per K		
$b_{23}$		Trend	Yes		mm/year		AR6 table 9.S (*1*)
$\sigma_{23}$		Noise*	No		mm/year		
$\varepsilon_{23}$		Noise	No	N(0, $\sigma_{23}$ )	mm/year	200	
$\beta_{23}$		Fingerprint	Yes	N(0, $\sum_{23}$ )		20	CMIP6 (*54*) (Historical + SSPs; zos, zostoga 1900-2100)
$\beta_{24}$	GIA	Fingerprint	No	N(0, $\sum_{24}$ )		30	Caron *et al*. (*90*), 5000 ensemble members
$\beta_{25}$	VLM residual	Fingerprint	No		mm/year	578	

**Fig. 1.**
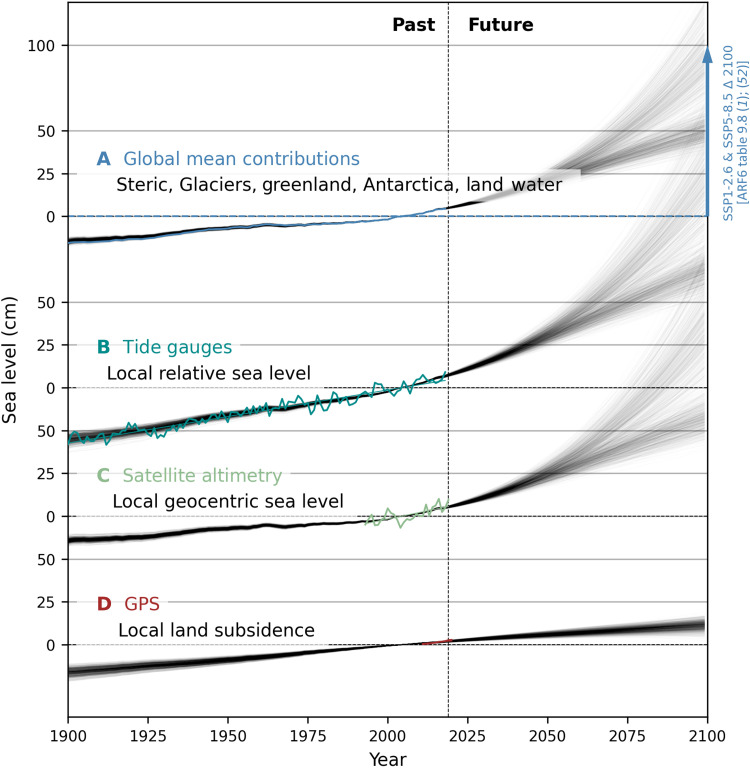
Schematic view of the constraints used in this study. Global sea level contributions (**A**) from thermal expansion, glacier loss, Greenland, and Antarctic ice sheet loss and land water storage changes. Historical global contributions are constrained by reconstructions. Future constraints are from IPCC AR6 for all contributions except for glacier regions. We include estimates for two future scenarios, SSP1-2.6 and SSP5-8.5. The local constraints consist of (**B**) tide gauge records, which measure relative sea level, (**C**) satellite altimetry, which measures GSL change, and (**D**) GPS, which measures VLM. The measurement period can vary between 1900 and 2018 for tide gauges and is 1993–2019 for satellite altimetry and 2000–2020 for GPS. We use the linear trend over the period of availability. The black thin lines are illustrative and show the time series produced by our model for the SSP1-2.6 and the SSP5-8.5 scenarios for the global mean sea level (total including land water contribution) (A) and for local relative sea level, GSL, and land subsidence in New York City in (B) to (D). See Materials and Methods for references and details on the input data.

Figure 1 gives an overview of the observations and constraints entering the model (see also Table 2). To constrain the model, we use the historical time series (1900–2018) for each source contribution from a recent sea level reconstruction study (Fig. 1A) (*13*). For glaciers, we use the data from (*51*, *52*) for 19 individual regions. We model peripheral glaciers with the glacier model and therefore remove their contribution from the historical time series for Greenland and Antarctica (*13*) to avoid double counting. This also ensures consistency with the IPCC future ice sheet estimates, which exclude peripheral glaciers. We also constrain the model with estimates of future sea level contributions, using the IPCC medium confidence estimates for each component for the year 2100. The IPCC provides the data for the low-emission shared socioeconomic pathway (SSP) 1-2.6 and the high-end pathway SSP5-8.5 in table 9.8 of (*1*), which largely span the potential future emissions range, and we therefore use these scenarios. The inclusion of the future constraints serves to integrate physical knowledge from more complex models. We do not use the global mean sea level rise from reconstructions as a constraint. This avoids double counting with local tide gauges and satellite altimetry that inform the reconstructions. Thus, reconstructed historical global sea level rise serves as an independent estimate for evaluating the global performance of the model.

**Table 2.** Constraints for the likelihood function. The year 2100 is an abbreviation for the cumulative sea level rise in 2100 relative to the reference period 1995–2014.

Model variable	Domain	Period	Type	Shape	Dataset
Glaciers (excl. uncharted)	19 regions	1901 to 2018	Observations, reconstructions (time series)	19, 118	Malles and Marzeion (*51*), Zemp *et al*. (*56*)
Greenland ice sheet	1 region	1901 to 2018	Observations (time series)	118	Frederikse *et al*. (*13*) without glacier region 5 (median)
Antarctic ice sheet	1 region	1901 to 2018	Observations, reconstructions (time series)	118	Frederikse *et al*. (*13*) without glacier region 19 (median)
Steric	Global mean	1901 to 2018	Observations (time series)	118	Frederikse *et al*. (*13*)
Glaciers	19 regions	2100	Model projections (RCP8.5 ∼ SSP5-8.5)	19	Marzeion *et al*. (*52*)
Greenland ice sheet	1 region	2100	Model projections (SSP1-2.6, SSP5-8.5)	2	AR6 table 9.8 (*1*)
Antarctic ice sheet	1 region	2100	Model projections (SSP1-2.6, SSP5-8.5)	2	AR6 table 9.8 (*1*)
Steric	Global mean	2100	Model projections (SSP1-2.6, SSP5-8.5)	2	AR6 table 9.8 (*1*)
Relative sea level	Local	1900 to 2019	Tide gauges (linear trend)	578	PSMSL (*41*, *42*), Frederikse *et al*. (*13*), CMIP6 (*54*) [zos piControl]
Geocentric sea level	Local	1993 to 2019	Satellite altimetry (linear trend)	578	Satellite altimetry (*44*), CMIP6 (*54*) [zos piControl]
Vertical land motion	Local	2000 to 2018	GPS observations, reconstructed VLM field (linear trend)	578	Hammond *et al*. (*43*)

The local historical observations enter the model as a set of multiyear trends from tide gauges (Fig. 1B and figs. S1 and S2) (*42*), satellite altimeters (Fig. 1C) (*44*), and GPS station data and a smooth VLM field obtained from GPS stations (Fig. 1D and fig. S3) (*43*). The time period for the trend calculation is 1993–2019 for satellite altimetry and varies between locations for tide gauges, within a maximum time span of between 1900 and 2018, depending on data availability (fig. S2). GPS records also vary in the time span they cover, but it is generally a period between 2000 and 2020, so we treat that constraint as describing a constant rate between these two years.

To isolate the sea level response driven by global climate change in our model, we need to account for measurement errors and unforced variability inherent in the real-world observational data. For global historical time series, we use a contribution-specific noise term. For the local observations, we estimate the satellite observation error building in (*53*), which includes aspects such as location-specific tropospheric and orbital corrections. We infer the tide gauge observation error from comparison with the satellite altimeter’s interannual variance and attribute the excess variance to the measurement error term. Alongside measurement errors, the likelihood includes a probabilistic description of temporally and spatially correlated, unforced ocean variability. We describe this variability using long preindustrial control simulations from the Phase 6 of the Coupled Model Intercomparison Project (CMIP6) archive (*54*) for which we adjust the variance to match the observed interannual variance from satellite altimetry. This ensures that each CMIP6 simulation has a plausible interannual variability on the timescale for which altimetry measurements are available, while also accounting for multidecadal variability not captured by the satellite record. We use the adjusted CMIP6 ensemble to calculate the covariance between residual trends across tide gauge locations over diverse time spans covering spatial, tide gauge–to–satellite, and autocorrelation through unforced ocean variability (see Materials and Methods).

We describe the model, the priors, and the likelihood in detail in Materials and Methods. Posterior samples are generated using the No-U-Turn Sampler (NUTS) as implemented in pymc (*55*) and represent the central result of this work.

## RESULTS

### Comparison to global mean sea level change in the past and future

**Fig. 2.**
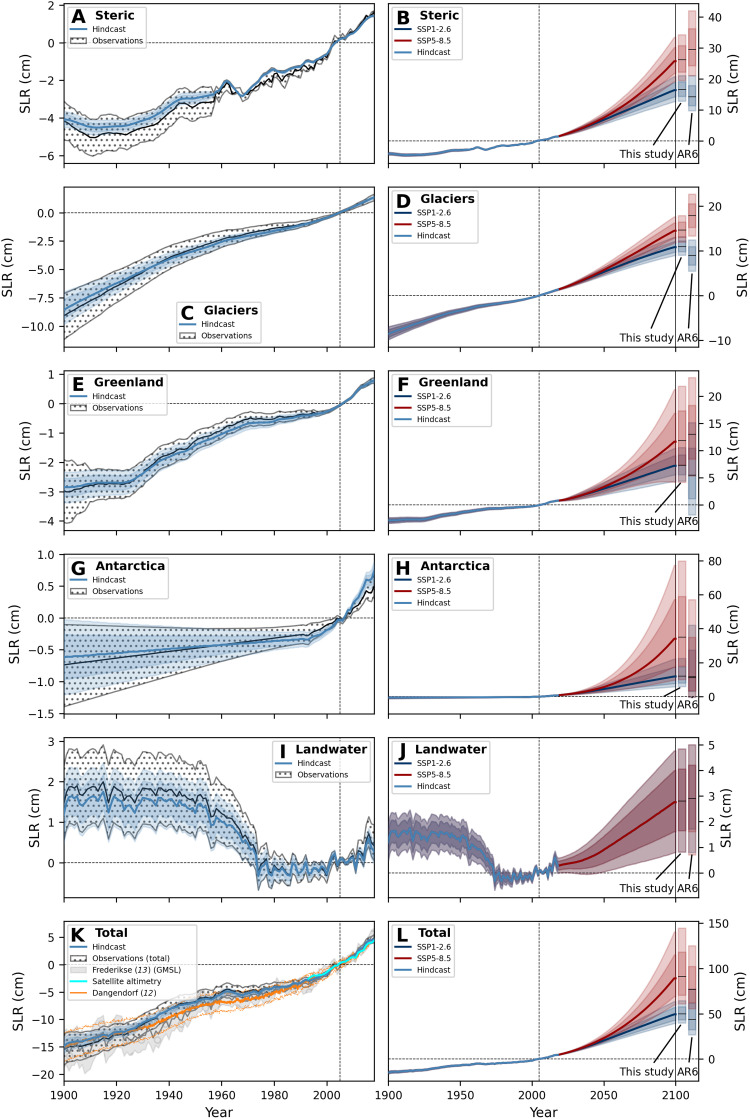
Components of global sea level rise from 1900 to 2100 (model posterior). Left panels (**A**), (**C**), (**E**), (**G**), (**I**), and (**K**) show model posteriors for the historical period with a thick median line and 67% and 90% range shading (light and very light blue), compared to observations (black line as median and dotted area as 90% uncertainty range). Right panels (**B**), (**D**), (**F**), (**H**), (**J**), and (**L**) show model posterior for the future period with median (thick line) and 67% (light shading) and 90% range (dark shading) for the SSP1-2.6 (dark blue) and SSP5-8.5 (red). Total sea level rise [(K) and (L)] is not used to calibrate the model, and we show independent historical reconstructions and satellite observations for comparison. Bars at the right compare our estimates and the IPCC medium confidence estimates for 2100 (median and 67% and 90% ranges). All data are shown relative to the IPCC reference period 1995–2014 with the vertical black dashed line indicating the center of this period. See Materials and Methods for references and details on input data. GMSL, Global Mean Sea Level reconstruction.

The posterior model ensemble shows that our model hindcast can generally capture the global historical evolution (Fig. 2, left) as well as the future IPCC constraints (Fig. 2, right bars). Peripheral glaciers are subtracted from the original Greenland and Antarctica time series in (*13*) and added to the glacier component to be comparable to our model results. Our model matches the most important of the 19 glacier regions but cannot fully capture historical glacier loss in some smaller regions (fig. S8), partly because it uses global rather than regional temperature as forcing, which limits its ability to reproduce regional details, particularly in the higher northern latitudes and the early 20th century, where the observations have larger error bars. Our total sea level rise estimate (Fig. 2, bottom) is the sum of the contributions and matches historical reconstructions well even though the total sea level rise is not used as a constraint in the model.

The posterior needs to reconcile multiple constraints within the model’s structure. To illustrate the calibration process and the role of the priors, we show, in addition to Fig. 2, the model output with only the prior and with only the global constraints (*1*, *13*, *51*, *56*), respectively. The prior (fig. S4) is wide by design, to encompass the historical observations (*13*, *51*, *56*) and IPCC future constraints (*1*), but is centered around the observed present-day rates for the source contributions in Eq. 1 (see Materials and Methods). Our conservative prior choice for ice sheets is visible with their almost symmetric distributions around the present-day baseline. The land water prior is sampled directly from past reconstructions and IPCC projections, and we therefore see a perfect match in the prior. Applying the global constraints (fig. S5), the model behaves as expected and shrinks the ranges to the uncertainty bands provided by global observations. The full posterior (Fig. 2) needs to additionally reflect the more complex landscape of local constraints. While small shifts in all components are visible, the Antarctic contribution is notable as it becomes more sensitive to global warming. The high similarity between the global-only-constraints version and the full posterior provides confidence that our model formulation is in line with the global and local constraints. We show versions of prior and global-only-constraints model output for the 19 glacier regions in figs. S9 and S10, which illustrate that the glacier behaves similarly as the other components along the two constraining steps.

The full posterior yields total global sea level rise projections (Fig. 2, right) that smoothly extend the historical records (Fig. 2, left). For the year 2100, they are 6 and 14 cm higher than the IPCC medium confidence projections (*1*) in the medians for SSP1-2.6 and SSP5-8.5, respectively (Fig. 2, bars in right). Under strong global warming, the main difference is Antarctica, whose higher sensitivity results in a median contribution that is 23 cm greater than in the IPCC medium confidence projections for SSP5-8.5. This is a consequence of the temperature-dependent parameterization for Antarctica in contrast to the IPCC (their median Antarctica medium confidence projections have no scenario dependency, but their uncertainty range increases for scenarios with stronger global warming). A version of the model without the local constraints aligns more closely with the IPCC (fig. S5). Alternative and equally valid formulations for the Antarctic contribution exist (see Discussion). A comparison of our component time series with the IPCC is shown in fig. S7.

### Consistency with tide gauge, satellite altimetry, and GPS

**Fig. 3.**
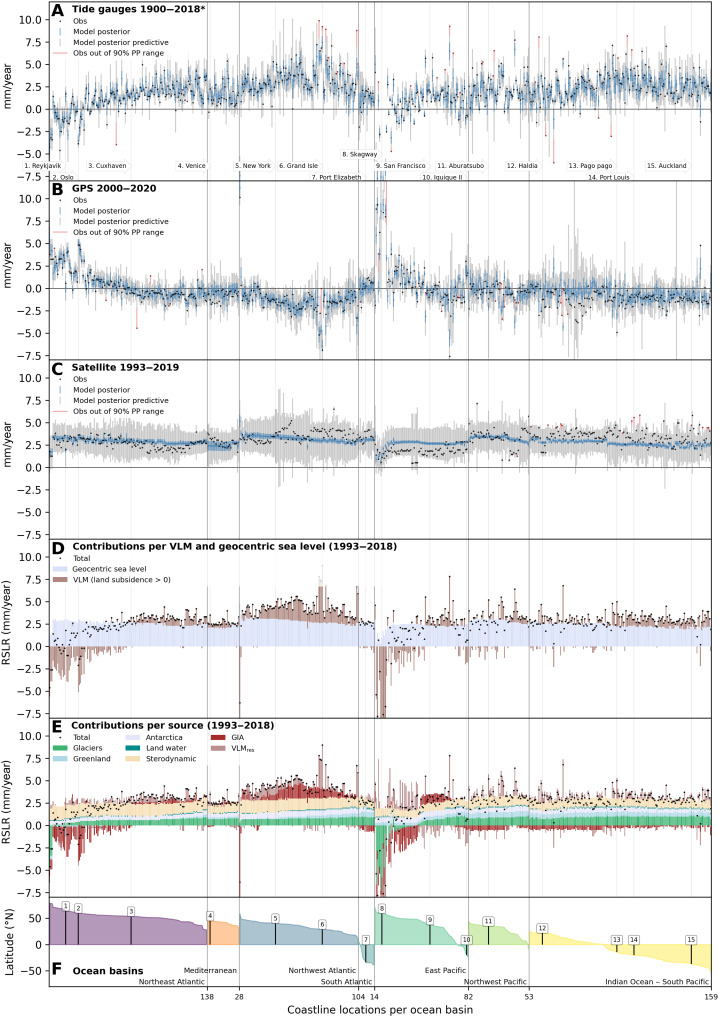
Sea level rise at individual tide gauges, constraints, and composition. (**A** to **C**) Local observational constraints to the model from tide gauges (A), GPS stations (B), and satellite data (C). For each tide gauge location, the black dot shows observations. The blue dot and bar show the median and the 90% range of the relative sea level posterior. The gray range shows the 90% range of the posterior predictive (PP) estimate. Any observation outside this range is marked as a red dot, with a red shading highlighting the distance. (**D**) Decomposition of relative sea level rise (RSLR) into GSL rise (light blue bars) and VLM (brown bars), between 1993 and 2018. Positive VLM indicates land subsidence and positive contribution to relative sea level rise. (**E**) Decomposition of relative sea level rise into the individual contributors. In both (B) and (E), contributors are set to the negative part of the *y* axis if they are negative and lead to sea level drop. Latitude and ocean basin of each tide gauge (**F**) with tide gauges sorted according to ocean basin and latitude. Locations presented in Figs. 3 and 4 are labeled in (A) and (F), and indicated in (A) to (E) as light gray vertical lines (see fig. S1 for map of locations). The period 1900–2018* in (A) is the maximum coverage length of tide gauges with most of them having shorter lengths. Ranges for some stations go beyond the *y*-axis limits to improve overall readability. VLM_res_, Vertical Land Motion residual.

The model is able to reproduce past relative sea level rise in a broad variety of tide gauge locations. We evaluate the predicted local trends with observations using the linear trends over their respective coverage periods indicated in Fig. 1. We show a comparison with tide gauges, GPS, and satellite altimetry observations for the historical period in Fig. 3. Tide gauge locations are ordered by ocean basin and latitude (Fig. 3F and fig. S1). As we do not simulate unforced oceanic variability, the output of the model (Eq. 1, model posterior) is the change in sea level without this variability (blue markers and range in Fig. 3, A to C). To make the output comparable to observations, which include unforced variability (tide gauges and satellite altimetry) and measurement error (tide gauges, satellite altimetry, and GPS), we additionally show the output of the posterior predictive sampling (gray range in Fig. 3, A to C). The posterior predictive sampling combines the model posterior distribution and the likelihood to predict the outcome of measurements, thus including unforced ocean variability and measurement errors as described in Eq. 15.

The observed trends (black dots in Fig. 3, A to C) lie within the 90% posterior predictive range for 94% of tide gauges, 95% of GPS estimates, and 97% of satellite altimetry data, and for all three constraints in 87% of locations. Where this is not the case, the observation is colored in red with the distance between the posterior predictive range and the observation highlighted.

The satellite altimetry linear trend from the 27-year record can have a strong imprint from unforced natural variability. Accordingly, the model posterior rarely matches satellite observations, but the posterior predictive range generally covers the observed fluctuations (Fig. 3C). The trend distribution of each observational dataset across locations (fig. S12) for the model posterior and posterior predictive shows that, particularly for satellite observations, an appropriate treatment of error and variability is necessary to reproduce the broad observed historical trend distribution. The impact of unforced oceanic variability is less pronounced for the tide gauge constraint (Fig. 3A and fig. S12A) because of the generally longer record length (fig. S2). The GPS posterior predictive range (Fig. 3B and fig. S12B) is wider than the GPS observations, which are based either on GPS station data when the station is less than 100 m away from the tide gauge or, for most cases, on a preprocessed smooth VLM field (*43*). The wider range is due to an error term added to the GPS data to adjust the low errors from the smooth field (see Materials and Methods and fig. S28C).

### Partitioning of past local relative sea level rise

The posterior estimate provides a detailed assessment of underlying contributions to local sea level rise (Fig. 3, D and E). We show the partitioning of relative sea level rise during the altimetry period into GSL rise and VLM in Fig. 3D and into all modeled components of relative sea level rise in Fig. 3E for the period 1993–2018. GSL rise dominates VLM as a contributor to relative sea level rise for the majority of locations (91%). Exceptions to this rule are largely located in the far northern latitudes, where isostatic rebound leads to a negative GIA contribution, and in the Gulf of Mexico (near Grand Isle in Fig. 3D), where anthropogenic influence leads to the sinking of land (*27*), which is captured by the residual VLM term in our model. Other locations with large VLM tend to be more scattered and often feature major residual VLM, which is indicative of land subsidence (or, occasionally, uplift) that cannot be explained by the other processes covered in the model. In 90% of locations, the residual VLM term is between −2.1 and 0.9 mm/year, with a median at −0.3 mm/year. The skewness toward negative values confirms the predominance of residual land subsidence compared to uplift. The sterodynamic contribution dominates over the mass-related contributions, including barystatic mass input and the associated gravitational, rotational, and deformational effects, to sea level rise (Fig. 3E) for the Northeast Atlantic coasts. In contrast, mass-related changes tend to dominate in other regions, especially the Pacific, in broad agreement with an ocean basin wide assessment (*13*).

### Future relative sea level rise

The model smoothly extends the past contributions to the future. Once calibrated, the model only needs a future global mean temperature pathway as input for projecting sea level rise at tide gauge locations, contrasting earlier works that combine data for a small set of fixed scenarios (*1*, *18*, *19*). All climate-sensitive components of local sea level rise adjust through the empirical relations between global mean temperature change and global sea level (Eq. 1). We illustrate this through local relative sea level projections for two illustrative mitigation pathways (IMPs) and one reference pathway of the IPCC AR6 WG3 report (*31*). The sustainable development pathway (IMP-SP) and the gradual strengthening pathway (IMP-GS) are compatible with the climate goals of 1.5°C and 2°C warming in 2100, respectively. The current policies (CurPol) reference pathway leads to median global warming of around 3°C in 2100. To focus on the spread in sea level response to climate and not on the climate response to emissions, we use the respective median global mean temperature response from 1900 to 2100 (*57*) to drive our sea level model, which is indicated with “*” in the figures (e.g., Current policies*).

**Fig. 4.**
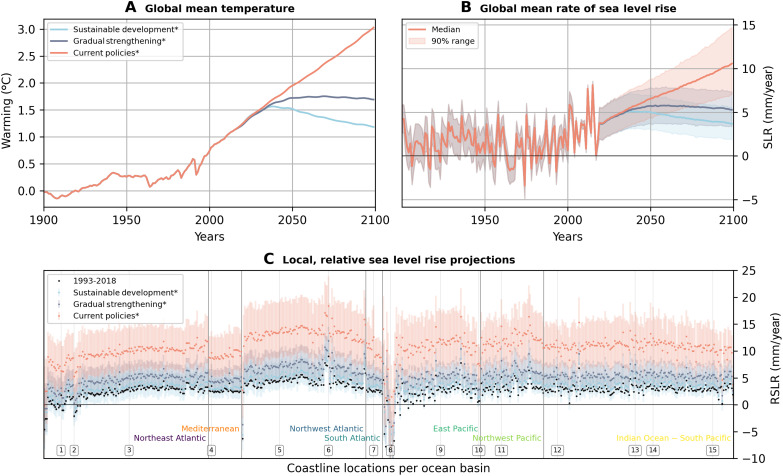
Past and projected sea level change for three IPCC AR6 WG3 scenarios. Median global mean temperature change (**A**), global mean rate of sea level change (**B**), and local rate of sea level change in 2100 (**C**) for the IMP-SP and IMP-GS and for the CurPol reference scenario. The shadings indicate 90% ranges. Local sea level rates for the 1993–2018 period are shown as black dots in (C). Ocean basins and featured locations are indicated following Fig. 3 and fig. S1. The asterisks (*) in the scenario names indicate that only the median temperature pathway was used for each scenario.

Figure 4 shows the global temperature response and the global sea level response together with the respective local relative sea level response at the tide gauge stations in 2100 as rates of change for the three IPCC AR6 WG3 pathways. We can infer measures relevant for climate policy from the projections. As an example, for the IMP-SP consistent with the Paris Agreement (*58*), the local rate in 2100 will be similar to the sea level rise experienced already today, but unprecedented if emissions follow current policies. Concretely, the local rate in 2100 will be in the median 1.3, 1.8, and 3.6 times the 1993–2018 rate in the IMP-SP, the IMP-GS, and the CurPol pathway, respectively (2.4, 3.5, and 8.2 times for the 95th percentile; the percentiles across samples and locations are reported). Figure 4 builds on individual median temperature pathways to illustrate sea level uncertainty in our model. In addition, we show projections that combine uncertainty from the temperature ensemble for each pathway and our sea level posterior in fig. S21.

### Relative sea level rise at 15 example locations

**Fig. 5.**
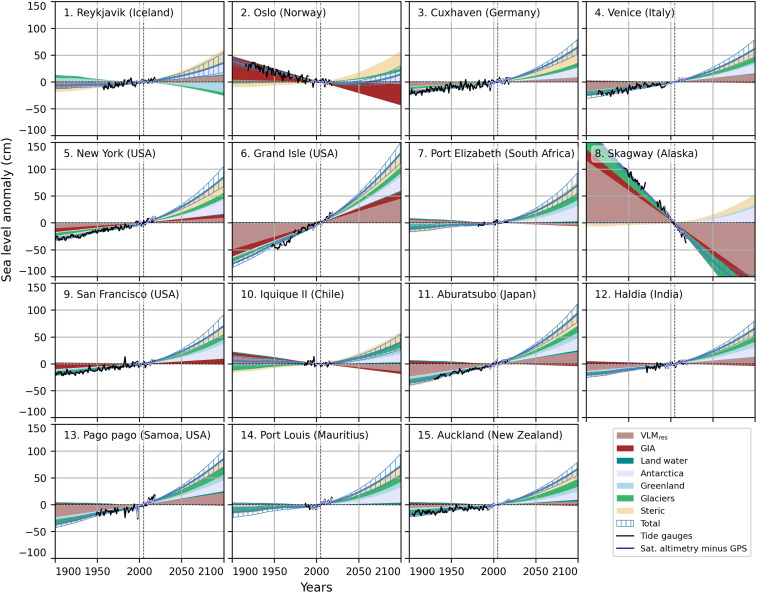
Past and projected (CurPol) relative sea level rise and its components at selected tide gauge locations. The shading shows median contributions of all individual components, and the blue line with 90% error bars shows relative sea level. Tide gauge records (black) and satellite minus GPS time series (dark blue with white) are overlaid. All data are shown relative to the IPCC reference period 1995–2014 with the vertical black dashed line indicating the center of this period.

We show in Fig. 5 how the different contributors add up to relative sea level rise in 15 example locations for the modeled period from 1900 to 2100. We show in fig. S22 a zoom to the historical period from 1900 to 2018. We use locations from (*21*) or replace them with nearby locations if exact matches are not available in the data that we use for correcting nodal tides (see fig. S1 for the map and details) (*13*). The locations are marked in Fig. 3, A and F. Our model closely aligns with observed tide gauge (black line) and satellite minus GPS (blue-white line) time series, partitioning relative sea level rise varyingly into the different contributions (color-filled curves). Stations such as New York and Cuxhaven are representative for most tide gauge locations where VLM from GIA and GSL rise add up to rising relative sea level. The model captures relative sea level fall as observed in Oslo (Norway) and Skagway (Alaska, USA) through a large VLM component. Skagway is an example for large residual VLM (the largest in this study) where robust land uplift cannot be explained by the modeled contributions (see Discussion). Grand Isle in the Gulf of Mexico and Pago Pago in American Samoa are other examples of large residual VLM. VLM in the Gulf of Mexico is affected through a multitude of processes not related to global climate change, including deltaic processes (in combination with the building of levees that isolate the surrounding wetlands from sediment supply from the Mississippi) and oil and gas extraction (*27*, *59*). A large earthquake in 2009 led to strong subsidence in the aftermath in Pago Pago (*60*).

Concerning future sea level rise, we show in Fig. 5 our model estimate for the CurPol pathway (*31*) at the 15 example locations. The rise is dominated by geocentric, climate-driven components. In particular, for most locations, the climate-driven share is larger than the residual VLM rates estimated from the past (Eq. 1). While Skagway is an exception and residual VLM contributes a major share of the projections by 2100, for three quarters of the 578 locations included in this study, the residual VLM rate contributes less than 12% of relative sea level in 2100 for the CurPol pathway, less than 16% for the IMP-GS, and less than 19% for the IMP-SP. This represents a decline from 29% for present-day rates.

We compare our results for the 15 example locations with the IPCC AR6 (*1*) for the SSP1-2.6 and the SSP5-8.5 scenarios (figs. S23 and S24, respectively). Our results are generally consistent with the IPCC. This is expected as we use IPCC AR6 future global estimates to constrain our model, a similar fingerprint approach to translate global to local estimates, tide gauge trend observations to constrain VLM, and the assumption that historical residual VLM continues linearly. A key difference is the temperature-dependent Antarctic contribution, which leads to higher median estimates and wide uncertainty ranges for temperatures far above the historical range (see Discussion). This leads to higher median total sea level rise and relatively high uncertainty ranges for SSP5-8.5 in our approach (uncertainty ranges comparable to the IPCC estimates) despite the constraining with the local data. In contrast, our uncertainty ranges for SSP1-2.6 are lower compared to the IPCC estimates.

**Fig. 6.**
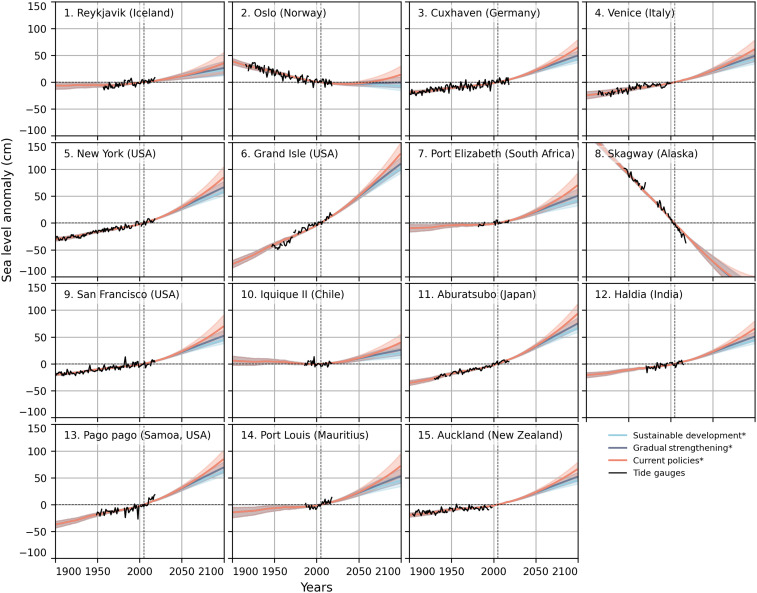
Past and projected sea level change for three IPCC AR6 WG3 scenarios at selected tide gauge locations. Lines show median relative sea level rise for the respective scenario, and shadings show 90% uncertainty intervals. Tide gauge records (black) and satellite minus GPS time series (dark blue with white) are overlaid. The asterisks (*) in the scenario names indicate that only the median temperature pathway was used for each scenario.

Figure 6 shows the relative sea level rise for the three IPCC AR6 WG3 pathways at the 15 example locations. For all the locations, except Oslo and Skagway, a future with continuing sea level rise is locked in for all three scenarios, but a difference between continued acceleration for the current policies pathway versus slowing sea level rise for the IMP-SP is visible. For Oslo, the IMP-SP versus the CurPol pathway means a shift from sea level stabilization to future sea level rise.

### Sensitivity to local constraints

We test how well the model can reproduce historical trends of one local observational dataset when constrained only by the other two (fig. S13, orange lines and shading). To have a point of comparison, we also include an experiment where none of the three local constraints are used (fig. S13, green lines and shading). In aggregate over all locations, the GPS and satellite constraints can almost make up for the lack of a tide gauge constraint (fig. S13A). Similarly, the GPS observations are largely reproduced with a combination of tide gauges and satellite altimetry, with a slight shift to higher values (fig. S13B). GSL appears mostly in line with the prior distribution and insensitive to which set of constraints are used (fig. S13C). A basin-scale analysis focusing on relative sea level offers a more mixed picture (fig. S14). While the experiment with GPS and satellite altimetry is able to explain 57% of the overall variance found in the observations against 85% in the experiment that includes tide gauges, in some basins, the inference skill is close to zero (South Atlantic and Northwest Pacific), and it is excellent in others (e.g., East Pacific). In the South Atlantic, only few tide gauges are available, and they can be largely affected by river discharge (*61*, *62*). The GPS coverage is also poor in that area. Both issues make cross-validation between these datasets challenging. The Northwest Pacific is heavily affected by earthquakes that often come not only with sudden but also with postseismic changes that violate our assumption of a linear GPS trend (*63*). There is a general improvement compared to not using any constraint at all (41% of observation variance explained), but the performance is degraded in some basins (Northeast Atlantic, South Atlantic, and Northwest Pacific) or shows minimal improvement (Indian Ocean–South Pacific). The clear beneficiaries of using satellite altimetry and GPS instead of no constraint are the East Pacific and the Northwest Atlantic. These areas have better GPS coverage (figs. S1 and S3), and more data points to perform statistics (fig. S2).

We also assess the sensitivity of future projections to the exclusion of local observational constraints, assumptions regarding local errors and VLM, and the inclusion of the IPCC low-confidence estimates for ice sheets (figs. S15 and S16). Overall, the projections are robust, with most choices resulting in only moderate changes. A consistent pattern, however, is that excluding observational datasets—whether GPS alone, GPS and satellite altimetry, or all three (GPS, satellite, and tide gauges)—leads to reduced local projections and narrower uncertainty ranges. This occurs because, in the absence of local observations, the residual VLM term is left to absorb the influence of the remaining constraints. When all three datasets are included (our default configuration), the residual VLM is more tightly constrained. In this setting, this is compensated by a more temperature-sensitive Antarctic component, resulting in higher median sea level projections and broader uncertainty ranges. A switch to IPCC low-confidence ice sheet projections [last column of table 9.9 in IPCC AR6 (*1*); fig. S17] leads to a broader uncertainty range for Greenland, while Antarctica is only slightly shifted upward (fig. S18). Overall, the resulting local projections stay very close to the default setting (figs. S15 and S16).

We illustrate the effect of withholding observations of 50% randomly sampled locations for the 15 example locations under the CurPol scenario (fig. S19). Our estimates remain largely stable under the perturbation, with the exception of locations exhibiting large residual VLM. When excluding the residual VLM component, sea level projections at withheld stations are consistently well captured (fig. S20). Skagway is again an outlier as the exceptionally high magnitude of VLM also involves higher-order GIA components that are not shared with nearby locations. Grand Isle and Aburatsubo, which also have large residual VLM, show stable projections in the other components when the station data are excluded.

### Comparison to IPCC regional uncertainties

**Fig. 7.**
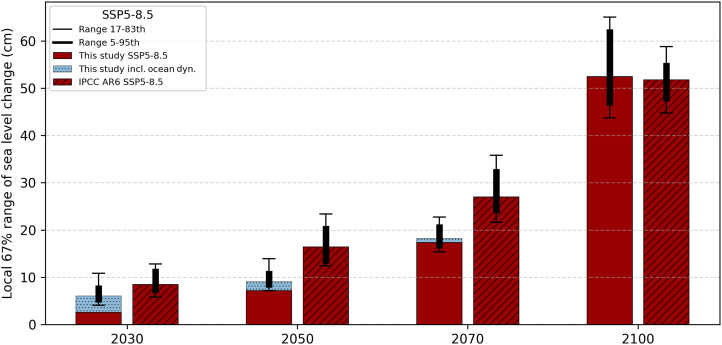
Distribution of uncertainty ranges across tide gauges. Median local uncertainty ranges are calculated for each of the 578 tide gauges included in this study. Bar heights show the median across sites, with whiskers indicating the 67% and 90% ranges, respectively, for the years 2030, 2050, 2070, and 2100. Results are shown for this study (red bars) including ocean variability (dotted blue bars) and the IPCC AR6 (hatched red bars) for the SSP5-8.5 scenario.

Last, we compare the local uncertainty ranges of our future relative sea level projections with those from the IPCC medium confidence projections (*1*, *64*), using SSP5-8.5 for illustration (Fig. 7). The reduced uncertainty in our projections before 2100 arises from the local constraining in combination with the time-continuous model, which allows for a bidirectional information flow between local and global components as well as past and future when estimating the posterior ensemble. Ocean variability is part of the likelihood in our approach and thus not constrained through observations. It is statistically independent from the other components of the model and is added on top of the constrained ensemble to enable comparison with the IPCC projections. Because the variance from ocean dynamics is approximately constant over time, while the climate-driven contributions grow, its impact on the total variance decreases over time. The IPCC AR6 simulations lack an initialization step with local and global observations, which results in simulations with larger- or smaller-than-observed rates of rise from day 1, and their uncertainty range is large in the early decades of their simulations. In contrast, it takes most of the 21st century for the local uncertainty to grow to levels comparable with those of the IPCC AR6. This is due to the fully dependent posterior ensemble across all contributions, in contrast to Monte Carlo methods that rely on independent sampling per component (*19*–*21*).

## DISCUSSION

Projections of the future are more trustworthy if consistent with past experience. For local sea level rise, this means that local sea level projections should be consistent with the decadal evolution of nearby observational data without being overly sensitive to shorter-term variability and measurement errors. So far, there has been no satisfying method available to fully integrate the available global and local knowledge on relative sea level rise to produce such projections. We present here a spatial Bayesian model, which fills this gap. The model enables projections of relative sea level rise and its components that emerge from the past observational data and evolve along a given global mean temperature pathway. Incorporating local data, it reduces the uncertainty of local relative sea level rise projections within this century. Our method bridges the gap between approaches that extrapolate historical trends (*24*, *65*) and projections from complex numerical models that lack feedback from local and often also global observations (*1*).

There are important limitations of the approach. Our choice of including unforced interannual variability from the ocean in the likelihood, instead of modeling the full time series explicitly, is valid to make projections ranging from decadal to longer timescales. However, the transient ocean state is not captured and therefore cannot be propagated forward for shorter forecasts. We use CMIP6 models to estimate the imprint of the interannual variability on the local multiyear sea level trends. Although we take several steps to mitigate the biases in such models (see Materials and Methods), we cannot rule out that coarse resolution and inadequate representation of coastal dynamics influence our results (*66*).

The local residual VLM contribution can become substantial when the sum of all other contributions cannot account for the historical local observations. While its contribution is small for most stations, the projection of the historical residual VLM into the future is speculative because there is no common large-scale process or driving force underlying it. We assume that the current rate of residual VLM continues to 2100 similar to (*18*). As many of the uncovered processes, including earthquakes and anthropogenic liquid extraction, can be nonlinear, our linear approach likely underestimates the true local uncertainty in places with relevant residual VLM (*63*). An alternative assumption is zero future residual VLM, as, for example, applied in (*19*) when it is known that anthropogenic activity led to subsidence or uplift in the past but has already faded [e.g., Tokyo (*67*)]. As we openly provide the dataset with all components including the residual VLM, different assumptions can be explored.

The residual VLM component can serve as an indicator of model skill: Relative sea level rise at locations where this term is small can largely be explained by known processes data included in our model, thus raising confidence in the projections. An outlier is Skagway, Alaska. Skagway and the neighboring stations have extremely rapid land uplift rates above 15 mm/year. The causes for the strong uplift include isostatic rebound following the rapid deglaciation of the Glacier Bay ice field after the Little Ice Age and the ongoing larger-scale regional deglaciation (*68*). Although we model glaciers in 19 regions and related uplift, the large residual VLM at Skagway suggests that our glacier and GIA fingerprints lack sufficient detail at such small spatial scales. We note that the tide gauge locations used in this study are concentrated in regions of relatively stable land. As many sites of strong land subsidence are highly populated and not within the tide gauge locations here, our finding does not rule out a potentially important role for VLM in the impacts of relative sea level rise.

Empirical relations between sea level components and global mean sea level change enable local relative sea level projections for new global mean temperature pathways as a smooth extension of the historical record. They have been criticized as they assume that the dominant processes in the period of observations will also dominate in the future (*69*). While this is an issue when using empirical relations to predict future sea level change from past data (*37*, *70*), it is less critical in the work presented here as we do not produce independent future sea level projections, but use empirical relations to integrate past and future local and global information. Our method incorporates the medium confidence estimates of future Antarctic ice loss from the IPCC AR6 (*1*), which are largely independent of the future warming scenario. We still make the modeling choice of a dependency on global mean temperature change while not incorporating the low-confidence projections for calibration.

The Antarctic contribution is highly uncertain, as past and present-day observations provide only limited constraints on its magnitude by 2100, due to the long response times to external forcing and potential instabilities of Antarctic loss (*1*, *71*, *72*). As the quadratic dependency on temperature leads to stronger dependence on warming than the IPCC AR6 medium confidence projections, the range for the SSP1-2.6 scenario becomes smaller than in AR6. As we cannot judge from current knowledge the true dependency on global warming of Antarctica (*73*) and Antarctic ice loss is steadily increasing in the observational records (*74*), we argue that our choice is equally valid to one that neglects dependence on global warming. Such choice is in line with the IPCC AR6 low-confidence projections. We assume that we can model the combined response of ice sheet surface mass balance and dynamic ice loss as a function of global mean temperature change. This has been treated differently in IPCC AR5 (*75*), where both contributions are separated and the ice dynamics part does not depend on global mean temperature change (*46*). A more recent study based on the AR5 approach assesses a temperature-dependent Antarctic ice dynamic contribution (*21*). We also simulate Antarctica and its contribution to regional sea level with a single model without a split between West and East Antarctica, assuming that the observed fingerprint of Antarctic ice loss will be valid within the 21st century. This is different to the IPCC AR6, which provides global numbers for Antarctica, but uses separate fingerprints for West and East Antarctica for regional projections. The relative contributions of the West Antarctic Ice Sheet (WAIS) and East Antarctic Ice Sheet (EAIS) may evolve between the recent past and 2100, which could influence projections in areas where the fingerprints differ most. The effect is expected to be smaller for regions far from Antarctica, as the fingerprints of WAIS and EAIS are relatively similar there. Ice modeling studies so far indicate low probability of relevant ice loss from East Antarctica within the 21st century (*76*), but our approach may still lead to overly confident uncertainty ranges for Antarctica.

For time horizons beyond 2100, rate limitations through the depletion of major ice basins in West Antarctica and the Greenland ice sheet can come into play. Local sea level projections with our method would thus need careful re-examination of the empirical dependence on global mean temperature, inclusion of physical bounds, or a switch to more physical models such as two-layer ocean models for thermal expansion (*77*, *78*). For glaciers, we already applied an upper physical bound through the available current glacier volume per region (see Eq. 2). More generally, regional climate change, which ultimately drives glacier and ice sheet mass loss, does not always have to be correlated to global climate change, so global mean temperature can be questioned as a predictor (*79*).

Further work should carefully consider how to apply the model to coastlines where tide gauges are sparse and where the residual VLM is poorly constrained. Our sensitivity experiment using satellite altimetry and GPS data but without tide gauges indicate that local sea level rise can be estimated without tide gauge data [see also (*80*)]. Such work would need further investigation and potentially more sophisticated treatment of the VLM component (*63*). Future work could also follow the reasoning in (*79*) and implement local climate indicators as predictors in sea level component models instead of global mean temperature. As is, the approach has a number of direct applications. In combination with simple climate models, the approach can be used to attribute observed damages of coastal flood events near tide gauges to sea level change or global climate change (*81*), to greenhouse gas emissions, or even to major emitters (*29*). The concept of committed sea level rise (*28*) could be extended in a study on the impact-relevant metric of local relative sea level rise and its components, including the reassessment of the approach for multicentury time horizons.

## MATERIALS AND METHODS

### General model formulation

The general modeling approach is described in the main text with Eq. 1. It represents the sea level change (*s* = 1) or VLM (*s* = 2) $y^{s}$ , relative to the Earth’s center of mass at any location *x* and time *t* between 1900 and 2100, as a function of global mean temperature *T* and time. The equation can be expanded in differential form on the annual rate of sea level rise ${y^{\prime }}^{s}$

$$\begin{array}{c} \begin{array}{c} y^{s}{\left(x,t\right)}^{\prime }=\sum_{k=1}^{19}\left[\left(a_{k}T\left(t\right)+b_{k}\right)V_{k}^{*}{\left(t\right)}^{1-m}+u^{\prime }\left(t\right)\gamma_{k}+\varepsilon_{k}\left(t\right)\right]\;f_{k}^{s}\left(x\right)\;19\;\text{glacier regions}\\ \;+\sum_{k=20}^{21}\left[q_{k}T^{2}\left(t\right)+a_{k}T\left(t\right)+b_{k}+\varepsilon_{k}\left(t\right)\right]\;f_{k}^{s}\left(x\right)\;2\;\text{polar}\;\text{ice}\;\text{sheets}\\ \begin{array}{c} \;+\left[\eta \left(t\right)+\varepsilon_{22}\left(t\right)\right]\;f_{22}^{s}\left(x\right)\;\text{land water storage}\\ \;+\left[a_{23}T\left(t\right)+b_{23}+\varepsilon_{23}\left(t\right)\right]F_{23}\left(x\right)\cdot \beta_{23}\;\text{sterodynamic}\\ \begin{array}{c} \;+F_{24}^{s}\left(x\right)\cdot \beta_{24}\;\text{GIA}\\ \;+\beta_{25}\left(x\right)\delta_{s,2}\;{\text{VLM}}_{\text{res}} \end{array} \end{array} \end{array} \end{array}$$ (2)

Each contribution described in Eq. 2 consists of (i) the climate-driven terms, whose parameters $a_{k}$ , $b_{k}$ , and $q_{k}$ (*k* = 1...21, 23) are a subset of $\alpha_{k}$ from Eq. 1; (ii) unforced natural variability or noise $\varepsilon_{k}$ ; and (iii) the associated local fingerprint $f_{k}^{s}\left(x\right)$ . For contributions *k* = 23–25, the fingerprints are calculated as a matrix product $F_{k}^{s}\cdot \beta_{k}$ that represents a parameterization of a spatial distribution, where $\beta_{k}$ is a random variable and $F_{k}^{s}$ is a set of spatial modes. Sea level change (*s* = 1) and VLM (*s* = 2) are always correlated through their common factor (the part of the product without superscript *s*). Parameters that are treated as random variables are marked in bold in Eqs. 1 and 2 and are summarized in Table 1. The terms “random variable,” “uncertain,” “sampled,” or “estimated” parameters are used interchangeably in Materials and Methods. All terms are described in detail in dedicated sections below.

#### Prior distributions for temperature-driven trends

Surface air temperature is taken as an anomaly with respect to 1995 to 2014. As a result, over that period, the mean sea level rate is exactly (*k* = 20, 21, 23) or closely (*k* = 1...19) associated with $b_{k}$ for the climate-driven terms. This allows us to specify prior distributions for this parameter that are centered on the data. However, we choose a prior variance larger than suggested by the data to account for the possibility that part of that trend may stem from natural variability. The temperature sensitivity parameter $a_{k}$ is constrained to be positive for the linear models with respect to temperature (*k* = 1...19, 23), consistent with our prior knowledge that the ocean expands and glaciers melt as they warm. This is achieved by an exponential distribution $\text{Exp}\left(\lambda \right)∼p\left(x\right)=\lambda e^{\lambda x}$ , where the parameter λ, indicated in Table 1, is chosen to generate a wide-enough sea level curve (fig. S4), before being constrained by the data. For the ice sheets (*k* = 20, 21), which are modeled with a quadratic dependency on temperature, we choose the values for $a_{k}$ and $q_{k}$ conservatively, centered on zero and with a wide-enough range to encompass the AR6 projections.

#### Unforced natural variability

Accounting for natural variability is essential to avoid attributing all of the existing measurements to a long-term climate trend, which would otherwise risk overconfidence in the estimated model predictions. We treat the $\varepsilon_{k}$ noise terms in Eq. 2 as latent variables and sample them accordingly. This is in contrast to the local, unforced sterodynamic variability, which is instead accounted for as part of observation errors (see the “Likelihood” section in Materials and Methods). Modeling the effect of natural variability on global contributions as latent variables ensures that the variability is propagated to the local level. The error models for the global components are summarized in Eq. 3

$$\begin{array}{cc} p\left(\sigma_{k}\right)∼\text{Exp}\left(1\right) & k=20,23\\ {\text{Cov}}_{k}\left[\varepsilon_{k}\left(t_{1}\right),\varepsilon_{k}\left(t_{2}\right)\right]=\left\{\begin{array}{c} \sigma_{k}^{2}e^{–|t_{1}–t_{2}|/5}\\ \sigma_{k}^{2}\delta_{t_{1},t_{2}}\\ \text{from observation data} \end{array}\right. & \begin{array}{c} k=1\ldots 19,20\\ k=21,23\\ k=22 \end{array}\\ p\left(\varepsilon_{k}\right)∼N\left(0,{\text{Cov}}_{k}\right) & \end{array}$$ (3)

We retain fairly simple models of normally distributed, uncorrelated white noise on the rate of sea level rise for the Antarctic ice sheet and thermal expansion (*k* = 21, 23), but use a 5-year autocorrelation timescale for the glacier and Greenland contributions (*k* = 1–19, 20). Tests for sensitivity suggest a longer timescale of 10 or 15 years for these contributions also produce plausible results, but larger autocorrelation timescales tend to reduce the robustness of the trend estimation. ${\text{Cov}}_{k}$ represents the autocovariance function for the contribution *k*, $\sigma_{k}$ a variance parameter, and δ the Kronecker operator. For the Greenland ice sheet and thermal expansion (*k* = 20, 23), we let the model estimate the variance of the noise from the data as part of the sampling. The associated parameters $\sigma_{20}$ and $\sigma_{23}$ are sometimes called hyperparameters and are included in Table 1. Otherwise, the noise parameters are fixed and estimated from the data. For the land water contribution, we sample its variability from data for the historical (*13*) and future period (*1*), as detailed below. To illustrate the role of noise for the modeled global sea level components, we show versions of Fig. 2 and fig. S8 on the rate of sea level rise (figs. S6 and S11).

#### Mountain glaciers

We simulate the glacier contribution for 19 regions outlined in the Randolph Glacier Inventory [RGI; (*82*)]. Glacier melt in regions *k* = 1,...,19 is assumed to be the sum of a term driven by global mean temperature (*83*), of unforced natural variability, and of the contribution from uncharted glaciers (*84*). In contrast to the other temperature-dependent terms, the mass change rate of mountain glaciers is proportional to, and limited by, their remaining volume [or surface area via a volume-area scaling exponent 1−m=0.76 ; (*83*)]. This creates a nonlinear differential equation for the volume (Eq. 4) for which $V_{k}^{*}\left(t\right)$ is the solution when solved separately from the variability and unchartered glacier terms

$$V_{k}^{*}{\left(t\right)}^{\prime }\overset{\underset{\text{def}}{}}{=}-\left[a_{k}T\left(t^{\prime }\right)+b_{k}\right]V_{k}^{*}{\left(t\right)}^{1-m}$$ (4)

$$V_{k}^{*}\left(t\right)\geq 0$$ (5)

$$\begin{array}{c} V_{k}^{*}\left(t\right)=\text{max}{\left\{0,{V_{2005,k}}^{m}+m\int_{2005}^{t}\left[a_{k}T\left(t^{\prime }\right)+b_{k}\right]dt^{\prime }\right\}}^{1/m} \end{array}$$ (6)

$V_{2005,k}$ stands for the observed glacier volume in 2005, which we treat as an uncertain parameter. We choose a prior distribution on the normalized parameters ${\tilde{a}}_{k}$ and ${\tilde{b}}_{k}$ to remain consistent across the variety of volumes and melt rates covered by the RGI

$$\begin{array}{l} {\tilde{a}}_{k}=a_{k}V_{0,k}^{1-m}/r_{0,k}\\ {\tilde{b}}_{k}=b_{k}V_{0,k}^{1-m}/r_{0,k} \end{array}$$ (7)

where $V_{0,k}$ and $r_{0,k}$ are constants chosen equal to the best estimate of present-day glacier volume and melt rate as reported in the literature (*1*, *51*, *56*, *85*, *86*). The prior distributions for $V_{2005,k}$ , ${\tilde{a}}_{k}$ , and ${\tilde{b}}_{k}$ are indicated in Table 1.

The contribution from uncharted glaciers *u*(*t*) according to (*84*) was found to be essential to close the sea level budget in the first half of the 20th century (*13*). We distribute it over the 19 regions via a fixed set of weights $\gamma_{k}$ . We use the lower and an upper estimate for the contribution of uncharted glaciers between 1900 and 2015 and the remaining uncharted volume yet to melt (*84*). We model each estimate as an exponential decay on the rate. We then sample the time series as a weighted sum between the lower- and the upper-bound time series, with a uniform probability in between

$$\begin{array}{c} \;u_{i}(t)=u_{i}(\infty )\left(1-e^{-t/\tau_{i}}\right)\;i=1,2\\ \;u_{i}\left(2015\right)-u_{i}\left(1901\right)=\left\{ \begin{array}{c} 48\;\text{mm}\;i=1\\ 16.7\;\text{mm}\;i=2 \end{array}\right.\\ \begin{array}{c} \;u_{i}\left(\infty \right)=\left\{ \begin{array}{c} 48+2.4\;\text{mm}\;i=1\\ 16.7+2.1\;\text{mm}\;i=2 \end{array}\right.\\ u\left(t\right)=u_{1}\left(t\right)w+u_{2}\left(t\right)\left(1-w\right)\\ \;w∼U\left(0,1\right) \end{array} \end{array}$$ (8)

where *i* is 1 for the lower bound and 2 for the upper bound, $u_{i}$ is the cumulative contribution from uncharted glaciers, and $\tau_{i}$ is the decay timescale, which we set to match the 1901–2015 contribution according to table 1 in (*84*). The weight w , which determines the total glacier contribution between 1901 and 2015 (Table 1), is the only free parameter for the uncharted glacier contribution. In (*84*), only a global estimate is made. We distributed this contribution in proportion to the present-day glacier volume (*13*) from the RGI (*82*), with weights

$$\gamma_{k}=V_{0,k}/\Sigma_{k=1}^{18}V_{0,k}\;\text{if}\;k\neq 19\;\text{else}\;0$$ (9)

We exclude Antarctica from the distribution because it has a small proportion of small glaciers despite its overall large glacier volume (*84*) , and the region is thought to have experienced little melt in the past, prompting Frederikse *et al*. (*13*) to consider the overall contribution of peripheral glaciers before 2003 to be zero. Natural variability is added as a noise term on the rate and is assumed to have a 5-year autocorrelation timescale (Eq. 3), and the variance is derived from the observations [(*51*) for *k* = 1, 18 and (*56*) for *k* = 19] (Table 2). For simplicity and ease of integration with the sampler, we add the terms from Eqs. 3, 6, and 8 separately, as shown in Eq. 2.

#### Polar ice sheets

For the Greenland and Antarctic ice sheet (*k* = 20, 21), we use a model with a quadratic dependence on temperature to account for nonlinear ice mass change. We use the quadratic term to enlarge the possible range of future sea level rise from these sources, under the control of the IPCC AR6 medium confidence constraints for the year 2100 (see the “Likelihood” section). We model unforced variability differently for Greenland and Antarctica (see the “Unforced natural variability” section), largely due to data quality differences. For Greenland, where good-quality data are available over a long time period [(*13*) and references therein], we let the model estimate the variance as part of the sampling (hyperparameter $\sigma_{20}$ in Table 1). The Antarctica observational data are sparser, and a coarse assumption for most of the 20th century was used in (*13*) (see fig. S6G). Using the constraint directly, we would underestimate the true historical variance. To circumvent the limitation, we diagnose the noise magnitude by analyzing residuals on a linear trend fit on data from 2003 to 2018 and derive a standard deviation of 0.41 mm/year. The noise magnitudes for both ice sheets can be seen in fig. S6 (E and G).

#### Land water storage

We do not model land water storage (*k* = 22) as a function of global mean temperature and instead sample the contribution directly from available datasets. We merge the dataset for the historical period in (*87*) with the AR6 future projections dataset (*64*). We then fit multivariate normal distributions individually to each period, which account for autocorrelation in the respective time series. The land water contribution is weakly scenario dependent, but, here, we only use the SSP5-8.5 scenario as the differences are small. The mean of this term appears as η(t) in Eq. 2, and its noise component with zero mean as $\varepsilon_{22}$ (see Table 1). For this contribution, despite the “noise” label, we do not formally distinguish between uncertainty in the long-term trend (caused by structural factors) and unforced natural variability. This is not necessary because we do not make new projections and instead rely entirely on the original datasets.

#### Mass redistribution fingerprints

We use the mass redistribution fingerprints $f_{k}^{s}$ (*k* = 1,...,19, 20, 21, 22) from (*13*). Fingerprints for individual glacier regions were obtained directly from the corresponding author of (*13*), and the others from a publicly available repository (*87*). The fingerprints for Antarctica and land water storage are variable in time in the repository, and we derive a fixed fingerprint by linear regression over the 2000–2018 period. Our modeling of Antarctica with a single fingerprint from observations is different to the IPCC AR6 (*1*), which uses separate fingerprints for West and East Antarctica to produce regional projections (see fig. S25 for a comparison).

#### Sterodynamic changes

We model the effects of thermal expansion and of sustained, long-term changes in ocean dynamics (*k* = 23) through fingerprints scaled by the global mean thermal expansion (*21*, *48*). Global mean thermal expansion is modeled in linear relationship to global mean temperature *T* (*88*). Here, we do not use a dependency on the rate of change of temperature (*48*, *88*) because rapid changes are already captured by the noise term. Our single-equation implementation is simpler than the methodology applied in IPCC AR6, which is based on emulation with a two-layer ocean model (*77*, *78*). We derive fingerprints from CMIP6 historical and future projections data (*54*) already corrected for drift from (*89*). We perform a linear regression of the local, zero–global mean sterodynamic sea level field (zos variable) against the global mean steric expansion in the same model (zostoga variable). We derive one fingerprint per climate model for a total of 21 CMIP6 models for which both variables are available. Instead of using the fingerprints directly as a discrete random variable, we convert the individual fingerprints into a continuous random variable by means of a singular value decomposition (SVD), with mean and covariance equal to the sample mean and covariance. This allows for more efficient sampling through the sampler’s gradient calculation. If *X* describes the matrix of fingerprints and $\tilde{X}$ is the multimodel mean fingerprint of shapes (21,n) and (1,n) , respectively, where *n* is the number of locations, then the SVD of its deviation from the mean reads $X-\bar{X}=\mathrm{US}V^{\prime }$ where *U*, *S*, and $V^{\prime }$ have shapes (21,29) , (20,20) , and (20,n) , respectively; *S* is a diagonal matrix of eigenvalues and $U^{\prime }U=V^{\prime }V=I_{20}$ . The expression in Eq. 2 is obtained by defining $\beta_{23}\overset{\underset{\text{def}}{}}{=}\left\{1,\beta_{23,1},\;\ldots ,\beta_{23,20}\;\right\}$ , where each $\beta_{23,j}$ is sampled from a normal distribution with zero mean and standard deviation given by $S_{i,j}$ , and the corresponding base fingerprints. $F_{23}\overset{\underset{\text{def}}{}}{=}\left\{\tilde{X},V_{1}\ldots V_{20}\;\right\}$ $\beta_{23,j}$  It can be shown that the mean and covariance of $F_{k}\cdot \beta_{k}$ matches the empirical covariance calculated directly from the 21 fingerprints. We assume here that the sea level changes related to ocean dynamics do not substantially impact VLM and thus only calculate the GSL fingerprint (*s* = 1).

#### Glacial isostatic adjustment

We model spatially correlated VLM and GSL changes (contribution *k* = 24 in Eq. 2) using a 5000-member ensemble of GIA simulations spanning a range of Earth rheologies and ice-load histories (*90*). The ensemble, obtained from the corresponding author, includes both GSL and VLM fields and is accompanied by likelihood weights. To reduce computational cost, we perform a likelihood-weighted SVD of the deviations from the ensemble mean. Specifically, we compute the SVD of the matrix

$$p^{1/2}\left(X-\mu \right)=\mathrm{W\Sigma}V^{⊤}≃W^{\prime }\Sigma^{\prime }{V^{\prime }}^{⊤}$$ (10)

where $X\in {\mathbb{R}}^{5000\times 2n}$ is the matrix of ensemble members (GSL and VLM), μ is the likelihood-weighted ensemble mean, $p\in {\mathbb{R}}^{2n\times 2n}$ is the diagonal matrix of likelihood weights, $V\in {\mathbb{R}}^{2n\times 2n}$ contains the empirical orthogonal functions (EOFs), $\Sigma \in {\mathbb{R}}^{5000\times 2n}$ contains the singular values, and $W\in {\mathbb{R}}^{5000\times 5000}$ is the weights. The prime symbol ( ${}^{\prime }$) on the right indicates their equivalent in reduced space, i.e., the truncated matrices with $W^{\prime }\in {\mathbb{R}}^{5000\times 30}$ , $\Sigma^{\prime }\in {\mathbb{R}}^{30\times 30}$ (diagonal), and $V^{\prime }\in {\mathbb{R}}^{30\times 2n}$ . We retain the first 30 EOFs, which explain 99.9% of the total variance and at least 97.5% of the variance at 97.5% of locations. We define the GIA rate as shown in Eq. 2

$$F_{24}^{\left(s\right)}\overset{\underset{\text{def}}{}}{=}\left\{\begin{array}{cc} V_{1},\;\ldots ,V_{n} & \;\text{for}\;s=1\\ V_{n+1},\;\ldots ,V_{2n} & \;\text{for}\;s=2 \end{array}\right.$$ (11)

$${y^{\prime }}_{24}^{\left(s\right)}\overset{\underset{\text{def}}{}}{=}\mu^{\left(s\right)}+F_{24}^{\left(s\right)}\cdot \beta_{24}$$ (12)

$$\beta_{24}∼N\left(0,\Sigma_{24}^{2}\right)$$ (13)

where $\beta_{24}\in {\mathbb{R}}^{30}$ is a parameter vector with variance equal to the singular values, and $F_{24}^{\left(s\right)}\in {\mathbb{R}}^{n,30}$ is the matrix of EOFs where the GSL component (*s* = 1) corresponds to the first *n* rows and the VLM component (*s* = 2) corresponds to the second *n* rows. This formulation preserves spatial correlation between GSL and VLM, incorporates the ensemble’s likelihood structure, and allows efficient sampling through a reduced parameter space.

#### Residual VLM

The residual VLM term (*k* = 25) does not affect GSL, indicated by $\delta_{s,2}$ , which is equal to zero for GSL and one for VLM. The VLM residual can represent a variety of processes from very local (mining) to regional (groundwater basins) or larger scales (GIA or climatic processes unresolved by our fingerprints). We sample the residual VLM term from a multivariate normal distribution with zero mean and 2 mm/year standard deviation and a spatial correlation structure of 100 km

$${\text{Cov}}_{25}\left(x_{1},x_{2}\right)={\left(2\;\text{mm}/\text{year}\right)}^{2}e^{-∥x1-x2∥/100\text{km}}$$ (14)

where the norm represents a spatial distance. The choice of 100 km is supported by a maximum in model performance metrics (fig. S26).

#### Global mean temperature

We use the IPCC AR6 global mean temperature pathways from the WG1 report Summary for Policymakers (*91*) for the historical period and SSP scenarios and from (*57*) for the IMP and CurPol pathway. We use both the median projections and the full ensemble. When using the full ensemble, we combine it with the posterior distribution via Monte Carlo sampling. In Eq. 2, the global mean temperature enters as an anomaly with respect to a 1995 to 2014 baseline.

### Likelihood

We calculate the likelihood p(obs∣y) of a model realization *y* matching a set of observations as a product of normal and multivariate normal probability distributions

$$\begin{array}{c} \begin{array}{c} \begin{array}{c} p\left(\text{obs}|y\right)=\prod_{k=1\cdots 21,24}\frac{\text{exp}\left[-\frac{1}{2}{\left(y_{k}^{\prime }-{\text{obs}}_{k}\right)}^{T}{\sum_{{\text{obs}}_{k}}}^{-1}\left(y_{k}^{\prime }-{\text{obs}}_{k}\right)\right]}{\sqrt{{\left(2\pi \right)}^{N}\left|\sum_{{\text{obs}}_{k}}\right|}}\;\text{past global time series}\\ \times \prod_{\begin{array}{c} m=2\;\text{for}\;k\leq 19\\ m=1\;\text{for}\;k=20,21,23 \end{array}}\frac{1}{\sqrt{2{\pi \sigma }_{k,m}}}\text{exp}\left\{-\frac{{\left[h_{k,m}\left(y_{k}\right)-\mu_{k,m}\right]}^{2}}{2\sigma_{k,m}^{2}}\right\}\;\text{ future global constraints} \end{array}\\ \times \prod_{\text{gps}\in \text{GPS}}\frac{1}{\sqrt{2{\pi \sigma }_{\text{gps}}}}\text{exp}\left\{-\frac{{\left[h_{\text{gps}}\left(y_{2}\right)-\mu_{\text{gps}}\right]}^{2}}{2\sigma_{\text{gps}}^{2}}\right\}\;\text{GPS}\;\text{trends}\\ \times \frac{\text{exp}\left\{-\frac{1}{2}{\left[h_{\text{tg},\text{sat}}\left(y^{3,1}\right)-\mu_{\text{tg},\text{sat}}\right]}^{T}{\sum_{\text{tg},\text{sat}}}^{-1}\left[h_{\text{tg},\text{sat}}\left(y^{3,1}\right)-\mu_{\text{tg},\text{sat}}\right]\right\}}{\sqrt{{\left(2\pi \right)}^{N}\left|\sum_{\text{tg},\text{sat}}\right|}}\;\text{tide gauges},\text{altimetry} \end{array} \end{array}$$ (15)

where $y_{k}$ and $y_{k}^{\prime }$ are the source contribution time series and their annual rates, as in Eqs. 1 and 2, respectively. $y^{1}$ and $y^{2}$ are the simulated GSL and VLM fields as in Eq. 1, variable in time and space. We also introduce *s* = 3 to denote relative sea level, which is $y^{3}=y^{1}-y^{2}$ . The operator h represents a transfer function or observation operator to match the simulated time series with normally distributed observations N(μ,σ) , or N(μ,Σ) in the multivariate case. The transfer function is generally based on a linear trend or a difference between two periods (see subsequent sections).

The first line of the likelihood represents the agreement between the modeled global contributions and their respective observed time series ${\text{obs}}_{k}$ , evaluated using a multivariate normal distribution. Each $y_{k}^{\prime }$ is compared with the corresponding observation using a covariance matrix $\Sigma_{{\text{obs}}_{k}}$ . This term includes contributions from glaciers, the ice sheets, thermal expansion and land water storage.

The second line captures constraints on projected global contributions in 2100 for specific scenarios of the IPCC AR6. For glaciers (*k* = 1–19), we use projections under SSP5-8.5 (*m* = 2), while for ice sheets and thermal expansion (*k* = 20, 21, 23), we use both SSP1-2.6 (*m* = 1) and SSP5-8.5. Each term is treated as a normal distribution with observational mean $\mu_{k,m}$ and standard deviation $\sigma_{k,m}$ . The Antarctic projections are modeled using a log-normal distribution to reflect the asymmetric distribution of possible outcomes. The corresponding transfer function thus operates on the logarithm of the modeled 2100 value. The IPCC estimates in table 9.8 of (*1*) are derived from probability boxes (*92*, *93*), which are different to probability distributions. Probability distributions are a good approximation for the probability boxes underlying the medium confidence IPCC projections used here, but not for the probability boxes underlying the low confidence IPCC projections [IPCC AR6 table 9.9 (*1*); fig. S17].

The third line describes the likelihood of observing VLM trends from GPS stations, given the simulated VLM. Each GPS observation is modeled as an independent normal distribution centered on the simulated value, with uncertainty $\sigma_{\text{gps}}$ . Where a GPS station lies within 100 m of a tide gauge, the station measurement is used directly; otherwise, we rely on the gridded VLM field from (*43*).

The final line represents the joint likelihood of observing tide gauge and satellite altimetry trends, based on the simulated relative sea level and GSL time series. Because local unforced ocean variability introduces correlations not captured by the model, we treat tide gauge and satellite observations jointly using a multivariate normal distribution. The transfer function $h_{\text{tg},\text{sat}}$ extracts the trends, and $\Sigma_{\text{tg},\text{sat}}$ denotes the full covariance matrix across locations and between measurement types. The covariance matrix is of shape N=2n , and ∣⋅∣ is the determinant operator.

#### Constraints on the global mean contributions

To constrain the past contributions of glaciers, we use two complementary datasets: the reconstructions in (*51*), which cover the period 1901 to 2018 but do not provide estimates for Antarctica, and reconstructions in (*56*), which begin in 1960 and include all 19 glacier regions. Following the method described in (*13*), we construct a synthetic ensemble by sampling from either dataset where both are available. The dataset in (*51*) includes 10 reconstructions based on alternative forcing combinations, which form the basis for their uncertainty estimate (uncertainties for each individual forcing are typically much smaller). We sample full time series when possible and individual years otherwise to preserve the autocorrelation structure of the underlying datasets. The glacier constraint does not apply to the uncharted glacier contribution.

For global mean thermal expansion and ice sheet contributions, we use observational time series compiled in (*13*), ensuring that peripheral glaciers are modeled separately as part of the glacier contribution. Because (*13*) includes peripheral glaciers with the ice sheets, we calculate the mean contribution from peripheral glaciers in our synthetic glacier dataset and subtract it from the ice sheet ensemble in (*13*) to avoid double counting. We also subtract the uncharted glaciers contribution. Both the data from (*13*) and our glacier dataset consist of 5000-member ensembles, from which we compute autocovariance matrices for each contribution $\Sigma_{{\text{obs}}_{k}}$.

Future global contributions are constrained using the scenario-based projections of the IPCC AR6 (*1*), specifically the global mean values in the year 2100 with respect to the 1995 to 2014 reference period for SSP1-2.6 and SSP5-8.5, as summarized in table 9.8 of the report. The provided percentiles are fitted with normal distributions for all components, except for Antarctica, for which we use a log-normal distribution to reflect the long-tailed behavior in the projections. For glaciers, future constraints are taken from the representative concentration pathway (RCP) 8.5 scenario in (*52*), applied to our SSP5-8.5 experiment. We omit constraints on the SSP1-2.6 experiment due to the reduced degrees of freedom in the glacier model.

#### Tide gauge data

We use the Revised Local Reference dataset from the Permanent Service for Mean Sea Level (PSMSL) (*41*, *42*), with a data cutoff in 2018. We select only records with more than 20 years of data and exclude those flagged for bad quality by PSMSL. We correct the data for the inverse barometer effect following (*94*) and for the nodal tide as in (*13*). Because the nodal tide dataset in (*13*) does not cover the entire PSMSL database, we further reduce the number of tide gauges to *n* = 578. In the likelihood calculation, we consider only the linear trends over the available period at each station. However, annual anomalies from these trends are also used, together with satellite data, to estimate tide gauge measurement error (see the “ Error covariance estimate for tide gauge and satellite altimetry” section). Tide gauges with data gaps are retained, and linear trends are computed using the available data. To ensure consistency when calculating error terms, we use only those ocean model data points that coincide temporally with available tide gauge observations, accounting for data gaps (see also fig. S27).

#### Satellite altimetry data

We use an aggregate, gridded product distributed by the Copernicus Climate Changes Service, version DT2021 (*44*), derived from 11 cross-calibrated satellite missions, from 1993 to 2019. The dataset is corrected for tide and weather, among various geophysical corrections (*95*). Of specific interest for this work, they apply a median filter within a 30-km strip along the coast, and they use an ice mask in high latitudes, which reduces coastal measurement errors by up to 10% compared to a previous version. The dataset is provided at monthly resolution, and we create an annual average version for use in this work. We add the provided correction for TOPEX-A instrumental drift to every grid cell as a spatially uniform term, which affects the first 5 years of the record. Only the linear trends between 1993 and 2019 for the satellite data cells neighboring the tide gauges are considered in the likelihood. However, residuals from the trend are also used (i) to correct the CMIP6 interannual variance and (ii) as input in our procedure to estimate tide gauge measurement error (see also sections below). There is no formal error attached to the satellite dataset. To calculate errors on the trends, we use a recent work by (*53*) to capture location-specific error estimates for the wet tropospheric correction, orbital drift, inter-mission bias, and high-frequency noise. We do not include their error attached to the GIA, because we model it explicitly here. No spatial correlation is included in their estimate. The end result is a trend error of ~0.5 mm/year standard deviation in most locations (see fig. S28B, “measurement error”).

#### Error covariance estimate for tide gauge and satellite altimetry

Annual tide gauge and satellite altimetry observations are related to simulated relative sea level and GSL in Eqs. 2 and 15 via

$$\begin{array}{l} {\text{obs}}_{\text{tg}}\left(t\right)=y^{\left(3\right)}\left(t\right)+\varepsilon_{\text{dyn}}\left(t\right)+\varepsilon_{\text{tg}}\left(t\right)\\ {\text{obs}}_{\text{sat}}\left(t\right)=y^{\left(1\right)}\left(t\right)+\varepsilon_{\text{dyn}}\left(t\right)+\varepsilon_{\text{sat}}\left(t\right) \end{array}$$ (16)

where $\varepsilon_{\text{dyn}}$ is the unforced ocean variability, and $\varepsilon_{\text{tg}}$ and $\varepsilon_{\text{sat}}$ are the measurement errors associated with tide gauge and satellite altimetry, respectively. Although the tide gauge and satellite measurements may come from different locations and time spans, we associate each tide gauge with its nearest satellite altimetry site. We neglect any influence of unforced ocean variability on VLM. In this work, we integrate the observations as a linear trend over the period of availability, which translates into

$$\begin{array}{l} \mu_{\text{tg}}=h_{\text{tg}}\left[y^{\left(3\right)}\right]+h_{\text{tg}}\left(\varepsilon_{\text{dyn}}\right)+{\tilde{\varepsilon }}_{\text{tg}}\\ \mu_{\text{sat}}=h_{\text{sat}}\left[y^{\left(1\right)}\right]+h_{\text{sat}}\left(\varepsilon_{\text{dyn}}\right)+{\tilde{\varepsilon }}_{\text{sat}} \end{array}$$ (17)

Here, $h_{\text{tg}}$ and $h_{\text{sat}}$ are linear operators that extract trends over the respective periods and locations covered by the tide gauge and satellite altimetry records, and ${\tilde{\varepsilon }}_{\text{tg}}$ and ${\tilde{\varepsilon }}_{\text{sat}}$ are the error terms after calculation of the linear trends, with variance ${\tilde{\sigma }}_{\text{tg}}^{2}$ and ${\tilde{\sigma }}_{\text{sat}}^{2}$.

Unforced ocean dynamics introduces correlations between tide gauges and satellite residuals, especially when the period of availability overlaps (Eq. 16). It also creates spatial correlations and autocorrelation. Its contribution is typically greater than the formal measurement errors (Fig. 3, A and B, and fig. S28). We model its impact on the linear trends as a multivariate normal distribution with zero mean and covariance matrix of size 2*n* × 2*n* where *n* is the number of locations, and 2 stands for the tide gauge and satellite altimeters. We note

$${\tilde{\varepsilon }}_{\text{dyn},i}=\left\{\begin{array}{cc} h_{\text{tg},i}\left(\varepsilon_{\text{dyn},i}\right) & \text{if}\;i\leq n\\ h_{\text{sat}}\left(\varepsilon_{\text{dyn},i-n}\right) & \text{if}\;i>n \end{array}\right.$$ (18)

$${\tilde{\varepsilon }}_{\text{dyn}}∼N\left(0,\Sigma_{\text{dyn}}\right)$$ (19)

To account for the covariance between the tide gauge and satellite altimetry trends, in space and over partially overlapping time windows, we generate synthetic realizations of $\varepsilon_{\text{dyn}}$ . We use data from the CMIP6 preindustrial control runs as proxy for unforced ocean variability of sea level (CMIP6 zos variable). The variable is first detrended by removing a quadratic trend in each location. We then apply a variance correction step in which we rescale the time series so that it has the same variance as the detrended satellite altimetry data over a 27-year period (1993–2019). For each variance-corrected CMIP6 model, we extract time series $\varepsilon_{\text{dyn},k=1\ldots n}\left(t\right)$ in 120-year windows (1900–2019) from the nearest valid model grid cell for each location, and we calculate the linear trends for the corresponding tide gauge ( ${\tilde{\varepsilon }}_{\text{dyn},k}$ ) and satellite altimetry sample ( ${\tilde{\varepsilon }}_{\text{dyn},k+n}$ ), thus obtaining a set of 2*n* trends ${\tilde{\varepsilon }}_{\text{dyn},i=1...2n}$ (Eq. 18) per time window. We repeat this step by sliding the window by 1 year as many times as the length of the preindustrial simulations permit. A covariance matrix is calculated from the resulting ${\tilde{\varepsilon }}_{\text{dyn}}$ samples. This procedure is applied for each CMIP6 model individually, and we then take the multimodel mean across all covariance matrices and use that as a proxy for the covariance from ocean variability

$$\Sigma_{\text{dyn}}\left(i,j\right)=\frac{1}{M}\sum_{m=1}^{M}\frac{1}{N_{m}-1}\sum_{l=1}^{N_{m}}\left({\tilde{\varepsilon }}_{i,l,m}{\tilde{\varepsilon }}_{j,l,m}\right)$$ (20)

where *m* refers to the CMIP6 models, *l* is an index for each sample, and $N_{m}$ the number of samples extracted for the model *m*. We exclude four models that have preindustrial control runs shorter than 200 years (AWI-ESM-1-1-LR: 100 years, GISS-E2-1-G-CC: 165 years, GISS-E2-2-G: 151 years, and KIOST-ESM: 150 years) and retain *M* = 53 models.

In fig. S29, we compare the resulting standard error ${\tilde{\varepsilon }}_{\text{dyn}}$ across all locations, for tide gauges and altimetry, with the formal error from fitting a linear trend to the observations, assuming an AR(1) model for the residuals. While for tide gauges, the distributions are comparable, for satellite altimetry, the CMIP6-based calculation used in this work generally yields larger errors. This confirms the advantage of using longer CMIP6-based surrogates to properly account for multidecadal oceanic variability.

Satellite trend errors ${\tilde{\varepsilon }}_{\text{sat}}$ are estimated after (*53*). To estimate tide gauge trend errors ${\tilde{\varepsilon }}_{\text{tg}}$ , we compare the annual variance in tide gauge records to that in satellite altimetry. Tide gauges typically experience larger high-frequency variability due to their coastal settings, often located in harbors or near river mouths, which expose them to wave action and river flow fluctuations. This expectation is supported by data: Residuals from linear trend fits show that the distribution of standard deviations is consistently broader for tide gauges than for satellite altimetry (fig. S30). We attribute the excess variance in tide gauges, relative to colocated satellite records, to measurement error

$$\begin{array}{c} \begin{array}{c} {\tilde{\sigma }}_{\text{tg}}=\left\{ \begin{array}{cc} \text{max}\left(\sqrt{\frac{\text{var}\left({\text{obs}}_{\text{tg}}\right)}{\text{var}\left({\text{obs}}_{\text{sat}}\right)}-1}\;{\tilde{\sigma }}_{\text{dyn},\text{tg}},0.1\;\text{mm}/\text{year}\right) & \text{if}\;\text{var}\left({\text{obs}}_{\text{tg}}\right)>\text{var}\left({\text{obs}}_{\text{sat}}\right)\\ 0.1\;\text{mm}/\text{year} & \;\text{otherwise} \end{array}\right. \end{array} \end{array}$$ (21)

where ${\tilde{\sigma }}_{\text{dyn},\text{tg}}$ is the imprint on natural variability on the tide gauge trends (the first *n* diagonal elements of $\Sigma_{\text{dyn}}$ ). This formulation neglects the impact of satellite measurement error on annual variance ( $\sigma_{\text{sat}}<<\sigma_{\text{dyn}}$ ). This term is labeled “measurement error” in fig. S28A and is treated as spatially uncorrelated, unlike ocean variability, which exhibits spatial structure. We impose a minimum tide gauge error of 0.1 mm/year to guard against underestimating uncertainties, even where tide gauge variance is lower than that of satellite altimetry. Tide gauge records are typically affected by hourly noise in the range of 1 to 2 cm (*96*). These random errors average out over time: For example, a daily error of 2 cm translates into an uncertainty of about 0.1 mm/year for a 5-year trend, decreasing with longer trend windows.

We obtain the full covariance matrix adding the measurement error variance on the diagonal

$$\begin{array}{c} \Sigma_{\text{tg},\text{sat}}\left(i,j\right)=\Sigma_{\text{dyn}}\left(i,j\right)+\left\{\begin{array}{cc} \delta_{\mathrm{ij}}\;{\tilde{\sigma }}_{\text{tg},i}^{2} & \text{if}\;i,j\leq n\\ \delta_{\mathrm{ij}}\;{\tilde{\sigma }}_{\text{sat},i-n}^{2} & \text{if}\;i,j>n\\ 0 & \text{otherwise} \end{array}\right. \end{array}$$ (22)

The resulting error variance is shown in fig. S28 (A and B), and the error covariance including all errors is shown in fig. S31.

#### GPS constraint

We use a VLM field that was specifically computed at tide gauge locations (*43*), which provides a smooth, time-invariant VLM rate from the GPS network. GPS stations are seldomly located in the direct vicinity of tide gauges, and notable variability has been documented for VLM over short spatial scales [e.g., (*67*, *97*)]. Hammond *et al*. (*43*) uses a median filter to remove outliers that are considered as not representative for a wider geophysical process.

For the few locations with a GPS station located less than 100 m away, we use the local GPS trend and formal error as calculated by the MIDAS trend estimator (*98*). The MIDAS method robustly estimates long-term trends in GPS time series by using median differences between annual positions, minimizing sensitivity to outliers and seasonal noise. For the remaining tide gauge locations, which are the majority, we use the smooth field from (*43*). The authors provide a formal error range for the GPS trend field. We assess their error range, calculated as a weighted mean of formal errors from neighboring stations, which is likely an underestimate of the true error at the tide gauges, so we propose a different calculation. We assume the true GPS trend at a tide gauge to be equal to the smooth field $\mu_{\text{gps}}$ provided in (*43*), plus a spatial error from the roughness of the VLM field $\varepsilon_{\text{space}}$ to quantify how much an actual location might differ from the smooth VLM field in (*43*), plus an error in time representing deviations from a linear trend $\varepsilon_{\text{time}}$ . Conditional on the model, we have

$$h_{\text{gps}}\left(y^{2}\right)=\mu_{\text{gps}}+\varepsilon_{\text{gps}}$$ (23)

with

$$\varepsilon_{\text{gps}}=\varepsilon_{\text{space}}+\varepsilon_{\text{time}}$$ (24)

We estimate the spatial error from the difference between smooth and true GPS trend at each of the neighboring GPS stations and the time error from the formal error provided in (*43*) from the MIDAS estimator. We assume both errors are independent and normally distributed, and we calculate their combined variance $\sigma_{\text{gps}}^{2}$ at the GPS stations. We then interpolate the variance at the tide gauge locations with a weighted median, consistent with the overall methodology from (*43*), and use the same distance-based weights. We assume that the resulting GPS error at tide gauges is normally distributed $\varepsilon_{\text{gps}}∼N\left(0,\sigma_{\text{gps}}\right)$ . Figure S28C shows how both error terms contribute to the total.

### Sampling

We use an established Bayesian inference algorithm, the NUTS (*99*) implemented in the pymc framework (*55*), to generate 4000 samples from four independent chains that approximate the posterior distribution of model parameters

p(θ∣obs)∝p(obs∣θ)p(θ)

This posterior distribution determines our model ensemble and is the basis for our probabilistic relative sea level rise projections. While the sampling is not entirely divergence-free (2% or less divergences per chain), the chains result in consistent posteriors for all components (see fig. S32). We report the full range across all chains.

## Supplementary Materials

### This PDF file includes:

Figs. S1 to S32 References
