# Supplementary material for: Relative sea level projections constrained by historical trends at tide gauge sites
Source: Sci Adv. 2025 Oct 1;11(40):eado4506. doi: 10.1126/sciadv.ado4506 (PMC12487886; doi:10.1126/sciadv.ado4506)
Supplement: Supplementary file 1 — Figs. S1 to S32 References [file sciadv.ado4506_sm.pdf]

Science Advances
AAAS

Supplementary Materials for

Relative sea level projections constrained by historical trends at tide gauge sites

Mahé Perrette and Matthias Mengel

Corresponding author: Matthias Mengel, mengel@pik-potsdam.de

Sci. Adv. **11**, eado4506 (2025)
DOI: 10.1126/sciadv.ado4506

This PDF file includes:

Figs. S1 to S32
References

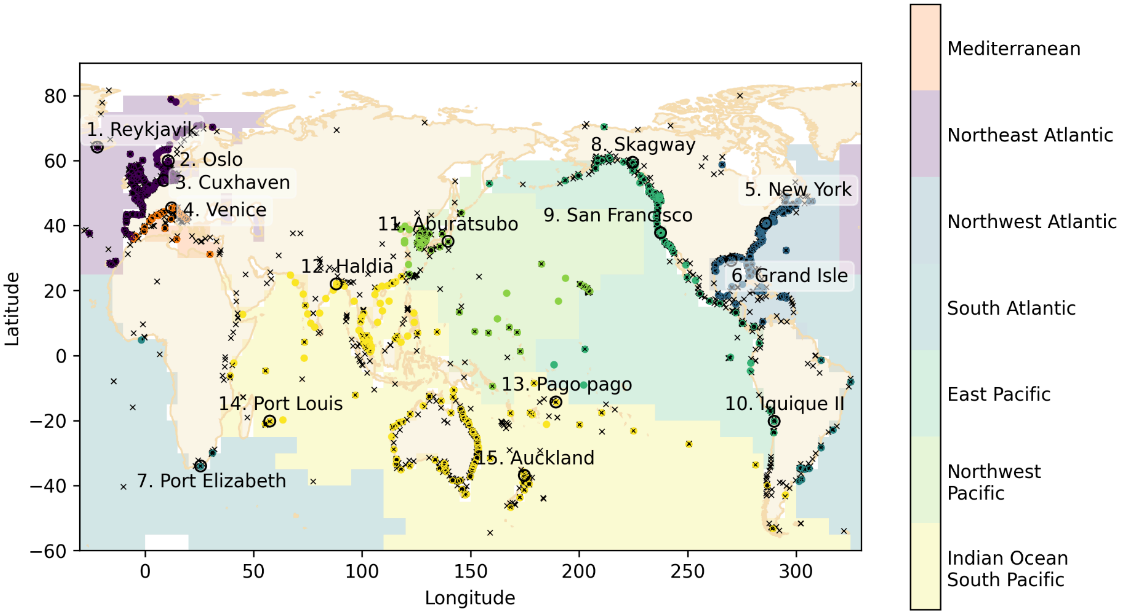

Fig. S1. Location of tide gauges, GPS stations and ocean basins. The colored dots show tide-gauge locations, and the black crosses show GPS stations that enter in the local GPS interpolation by (*43*). The basin definitions are adapted from (*100*), with the exception of the North Atlantic, redefined into Northeast Atlantic, Northwest Atlantic and Mediterranean to ease reporting. Locations featured with black circles are used as examples throughout this study, selected following (*21*) whenever possible, or in their vicinity when nodal correction data (*13*) was not available, to reflect a variety of sea level regimes. Nearby locations are Aburatsubo, Haldia, Iquique II and Port Elizabeth for the stations Mera, Diamond Harbour, Lima and Simons Bay in (*21*), respectively. See their Fig. 1 for comparison. We also include Venice (Italy) and Grand Isle (US, Gulf of Mexico) for interest in their residual VLM and to have 15 stations in total we leave out the Barentsberg, Newlyn, Palermo and Stanley II stations.

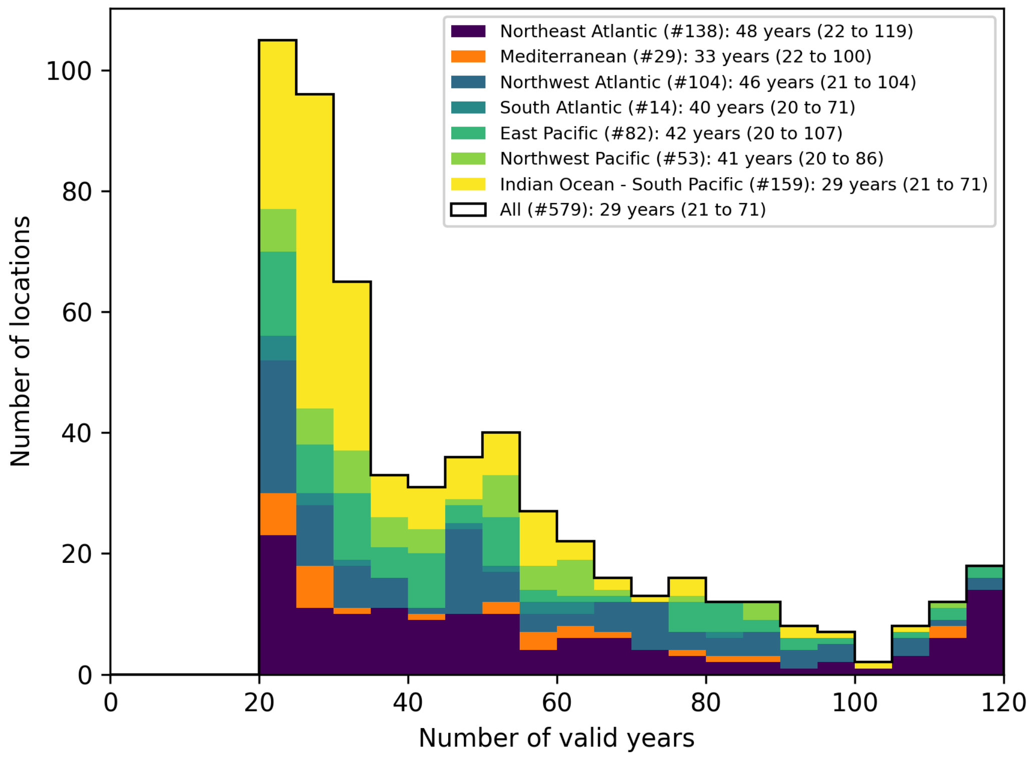

Fig. S2. Tide-gauge data availability per ocean basin. The legend indicates the number of tide-gauge stations considered per ocean basin, the median record length and the 90% range of the record length. A minimum of 20 years of data availability between 1900 and 2018 is requested.

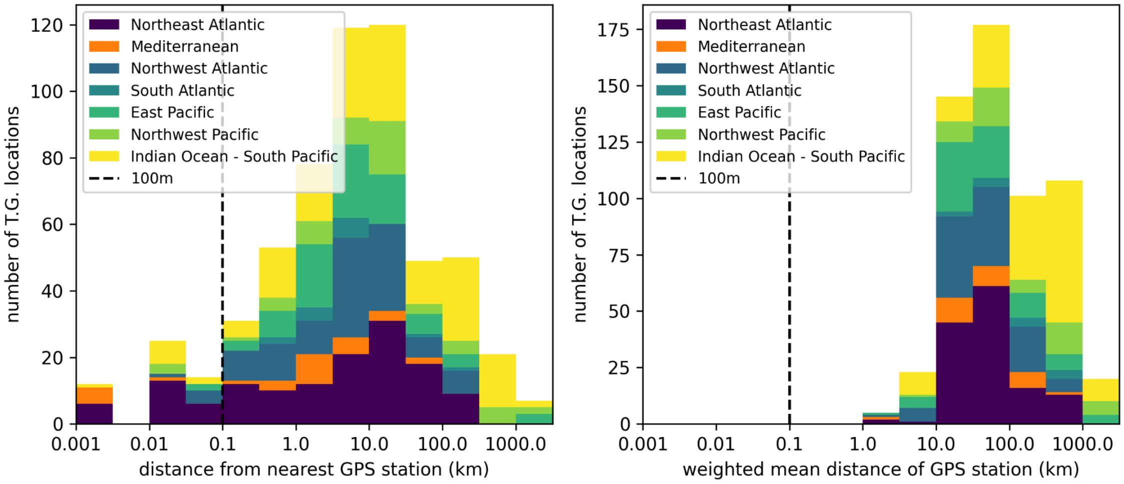

Fig. S3. Distance to GPS station, per ocean basin. Distance of the nearest GPS receiver (**left panel**), and weighted-mean distance from GPS receivers used in this study (**right panel**), following the methodology of (*43*). For the largest number of locations, the nearest GPS receiver lies around 10 km away. The vertical bar is the 100 m threshold below which we use the GPS station data directly, instead of the smooth VLM field by (*43*).

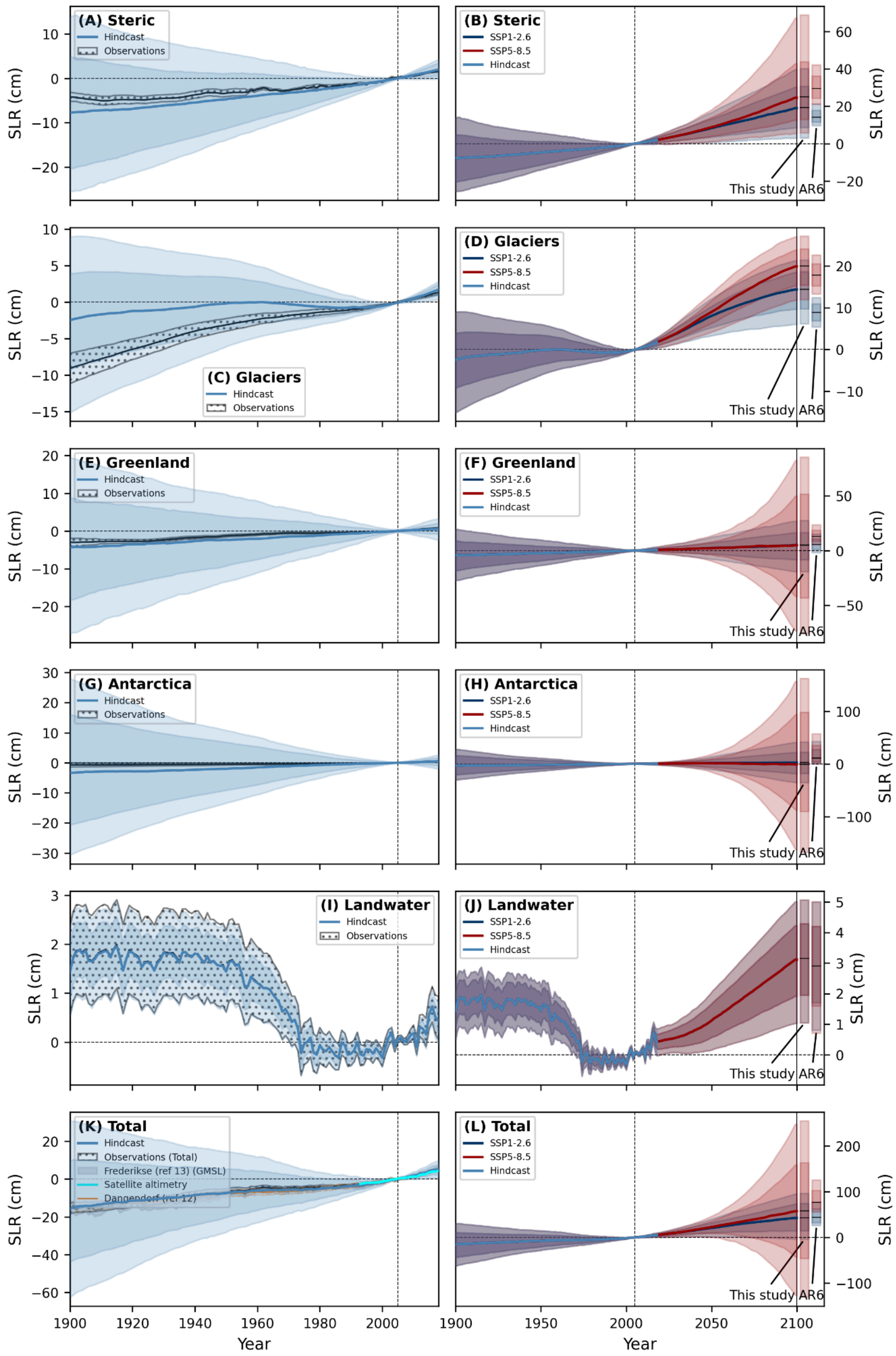

Fig. S4. Components of global sea level rise from 1900 to 2100 (model prior). Like Fig. 2, but showing the unconstrained model output only determined by the priors and not by the likelihood.

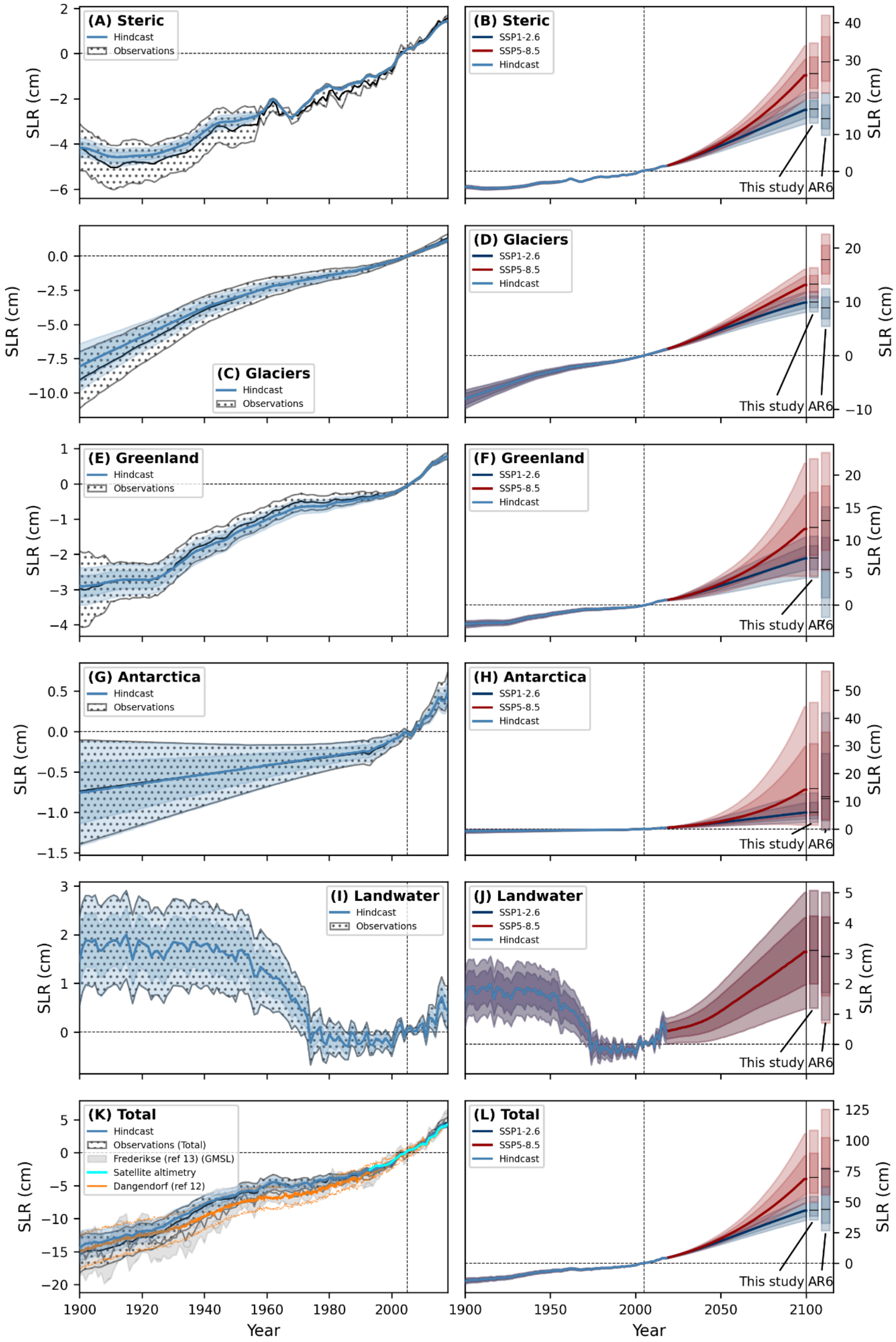

Fig. S5. Components of global sea level rise from 1900 to 2100 (global constraints only). Like Fig. 2, but only with global constraints applied, so without the local tide gauge, GPS and satellite data constraints.

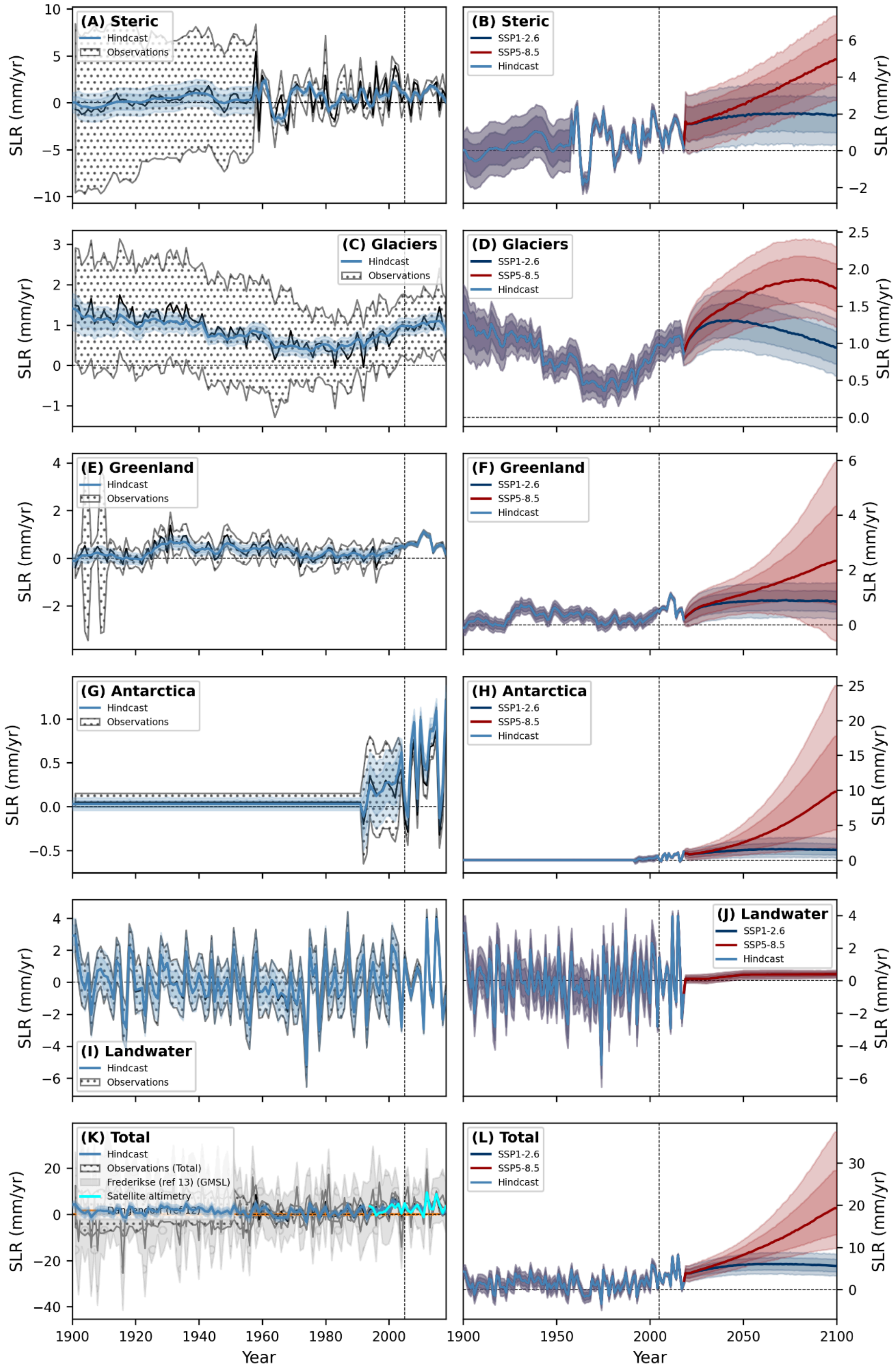

Fig. S6. **Components of global sea level rise from 1900 to 2100 (rates).** Like Fig. 2, but showing the rate instead of cumulative sea level rise.

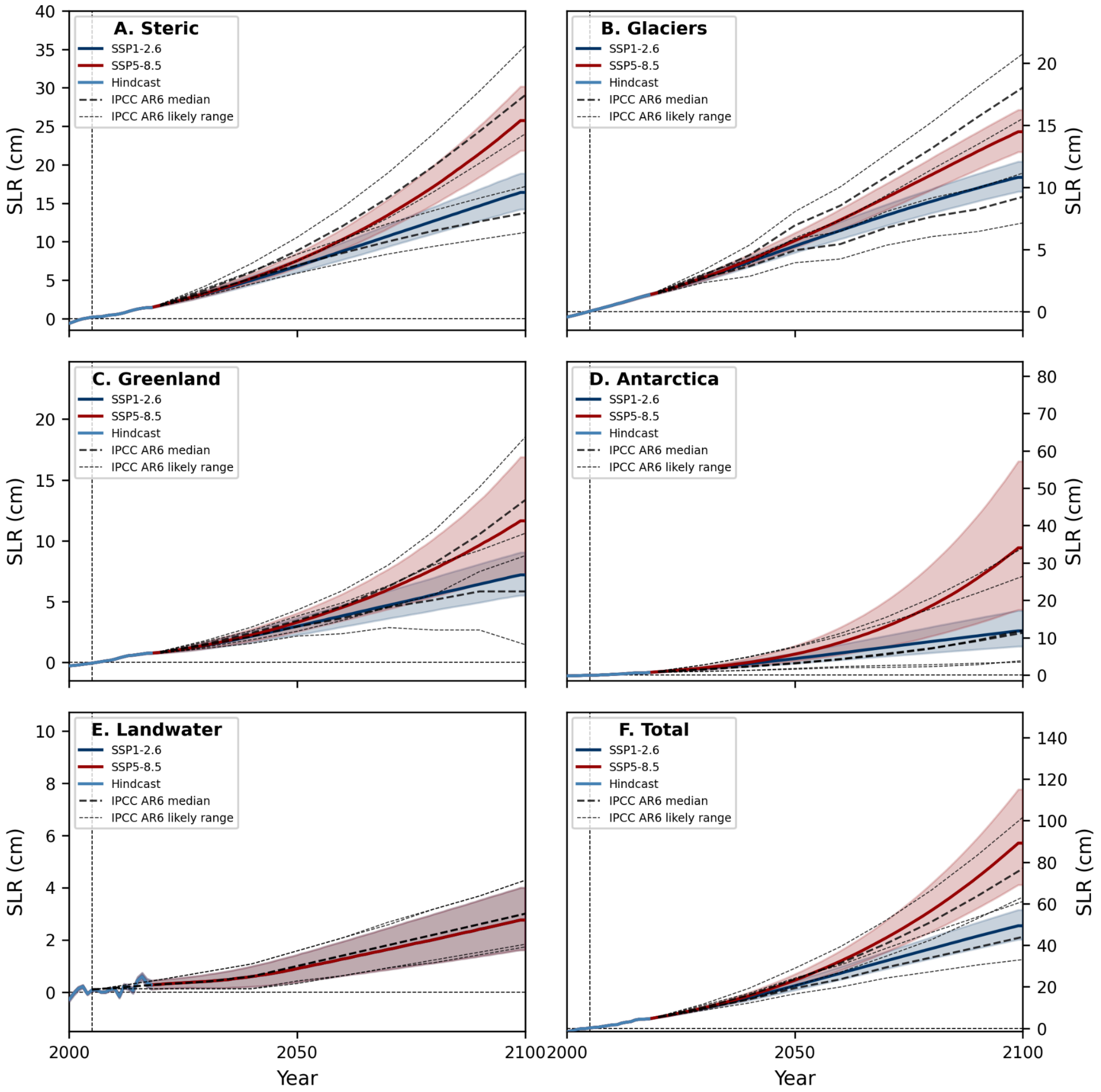

Fig. S7. Comparison of timeseries global projected contributions. We compare the time series of our results (as shown in Fig. 2) to the medium confidence timeseries projected by the IPCC AR6 (*64*). Colored shadings indicate our 67% uncertainty range. Thin dotted black lines indicate the IPCC likely range of the medium confidence projections. Much of the differences in the time series are due to differences in the projected 2100 numbers except for Greenland, which has a wider uncertainty towards the end of the 21st century in the IPCC projections.

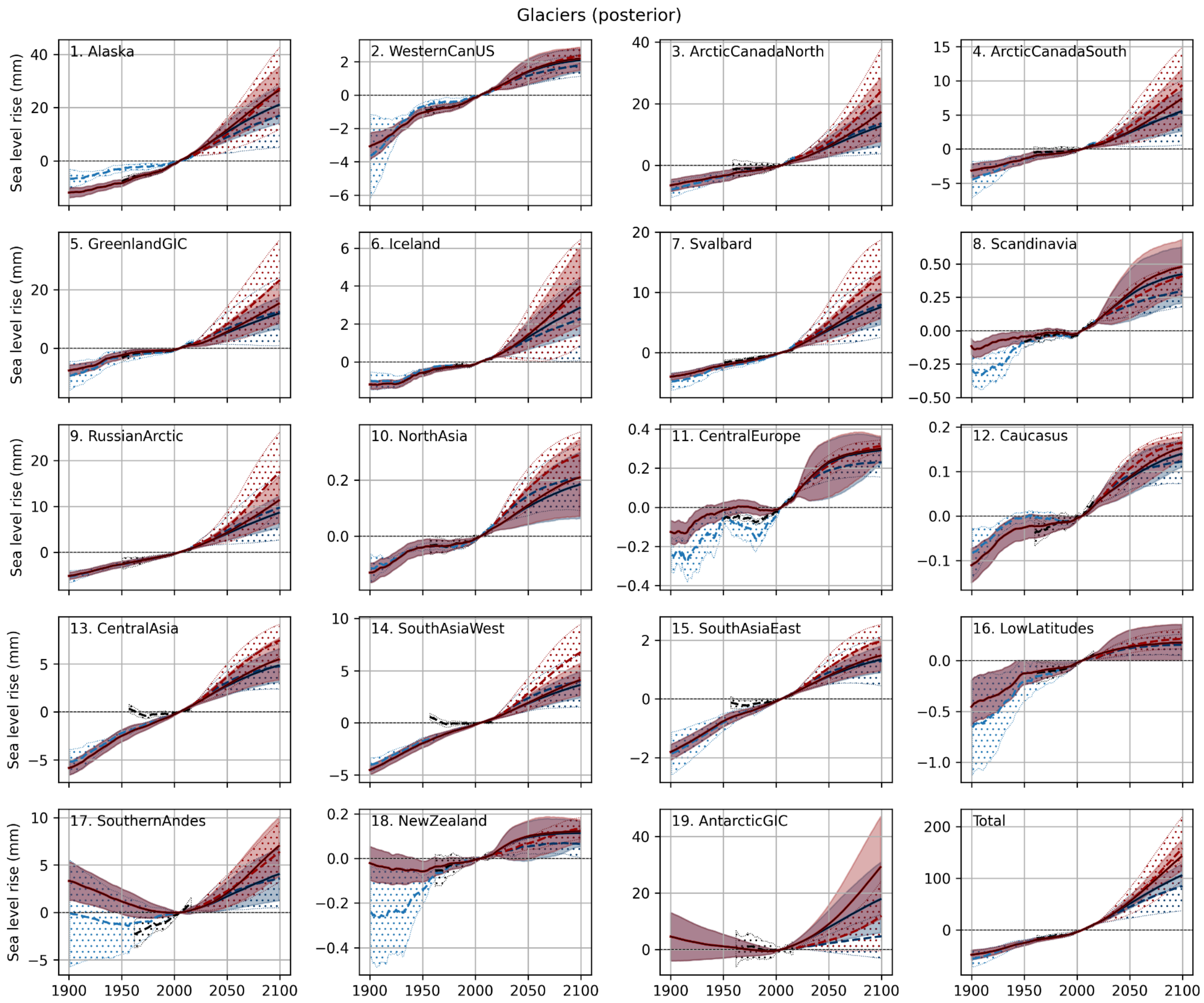

Fig. S8. Posterior glacier model simulation including local constraints. We show the model posterior (solid colored lines as median, shading as 90% range), for historical, SSP1-2.6 (green) and historical and SSP5-8.5 (red). The blue and black hatched lines with dotted 90% range for the historical periods are reconstruction by (*51*) and (*56*), which were both resampled following (*13*) to be used in the calibration. The hatched lines with dotted 90% range for the future period are projections by (*52*) for RCP2.6 (green) and RCP8.5 (red). As our model uses global and not regional temperature as forcing, not all regional details are fully captured, in particular in the higher Northern latitudes and the earlier 20th century. Comparison with fig. S10 shows how local tide gauges, satellite altimetry and GPS constraints change the posterior. All data is shown relative to the IPCC reference period 1995-2014.

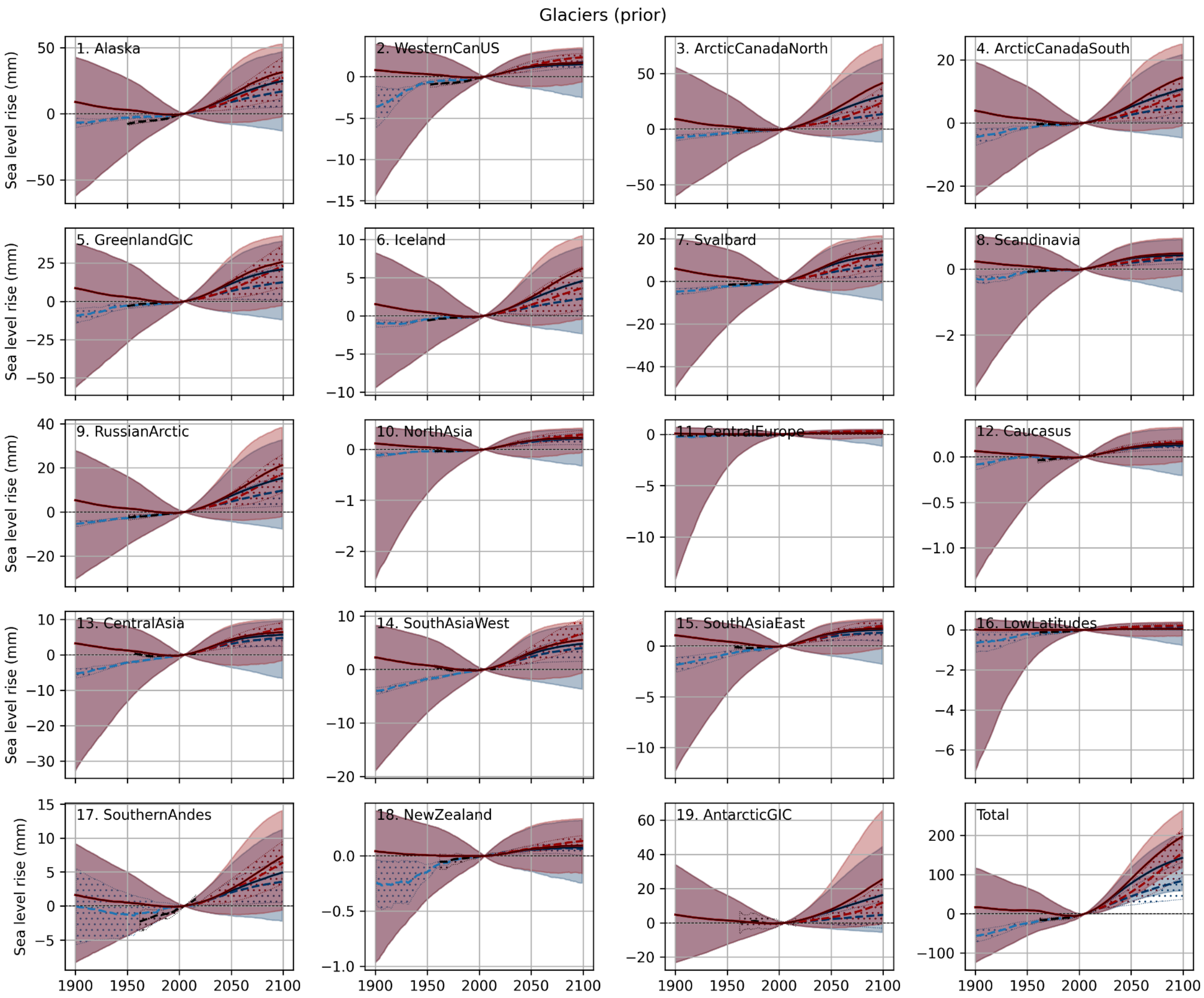

Fig. S9. Glacier model simulation (model prior). Same as fig. S8, but showing the unconstrained model output. The output is here only determined by the priors and not by the likelihood. See also fig. S4.

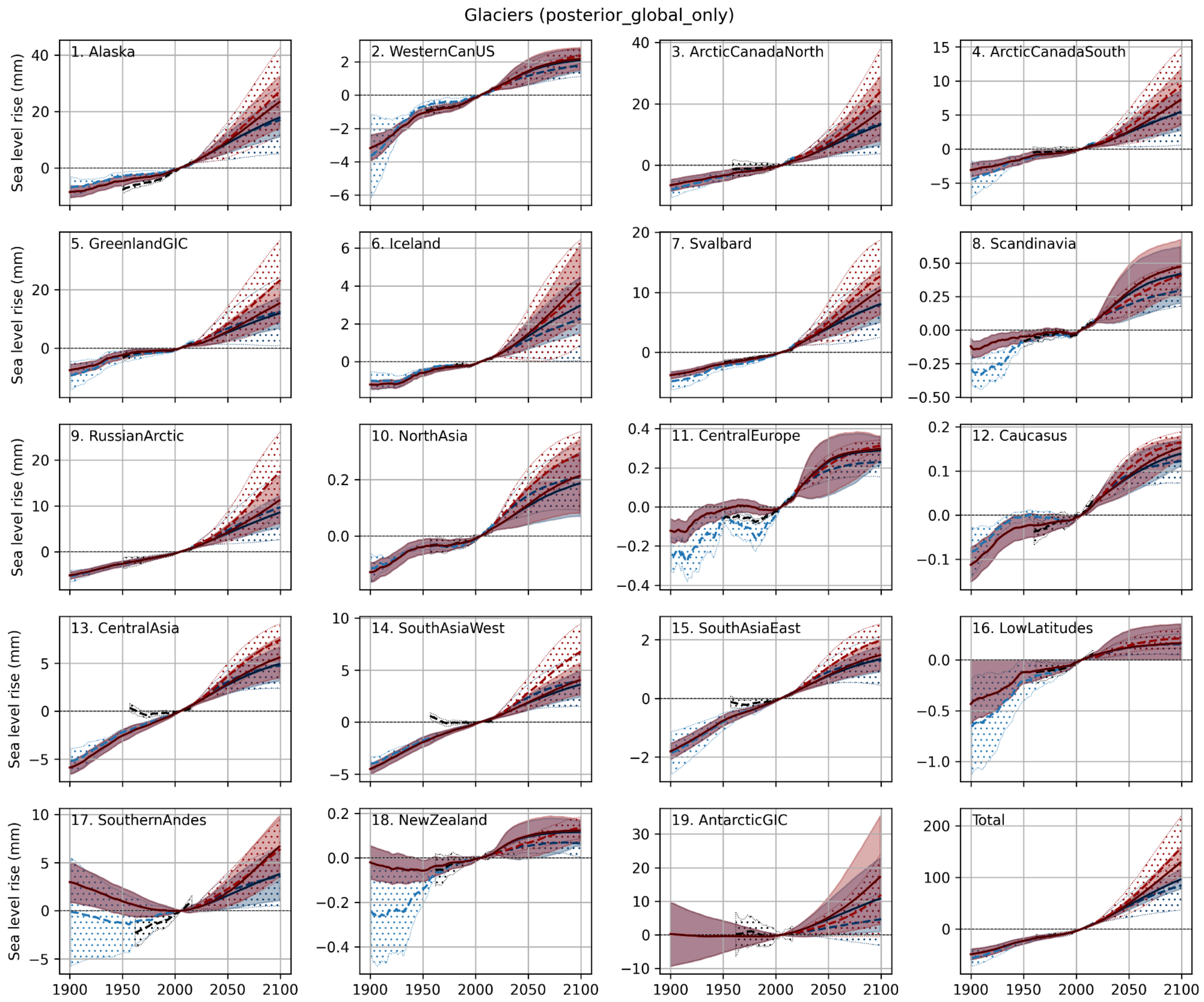

Fig. S10. Posterior glacier model simulation (global constraints only). Same as fig. S8, but with only global constraints applied. The model output shown is from simulations where the local tide gauge, GPS and satellite data constraints were not applied. See also fig. S5.

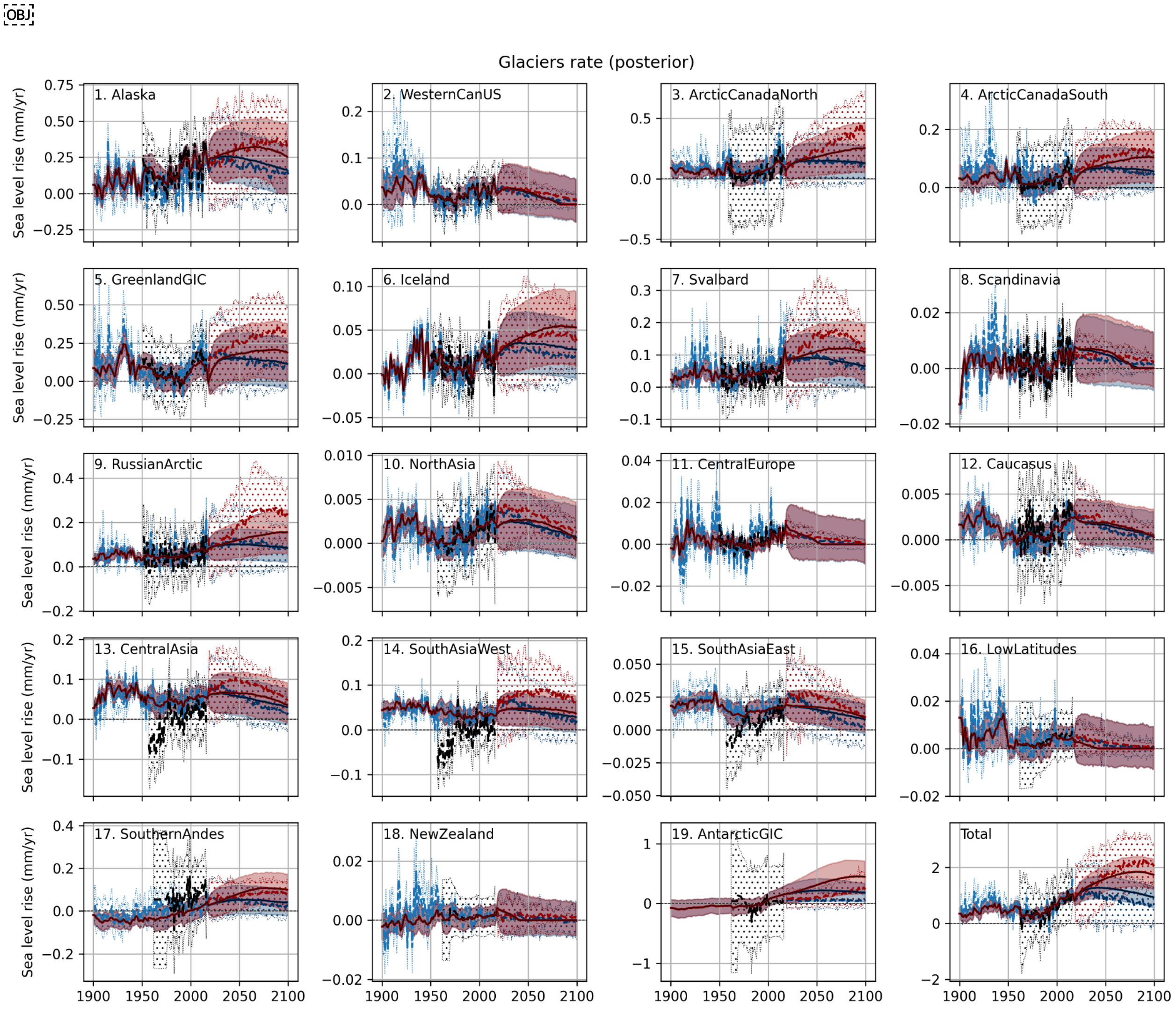

Fig. S11. Posterior glacier model simulation including local constraints (rates). Same as fig. S8, but for the rate instead of the cumulative sea level rise contribution.

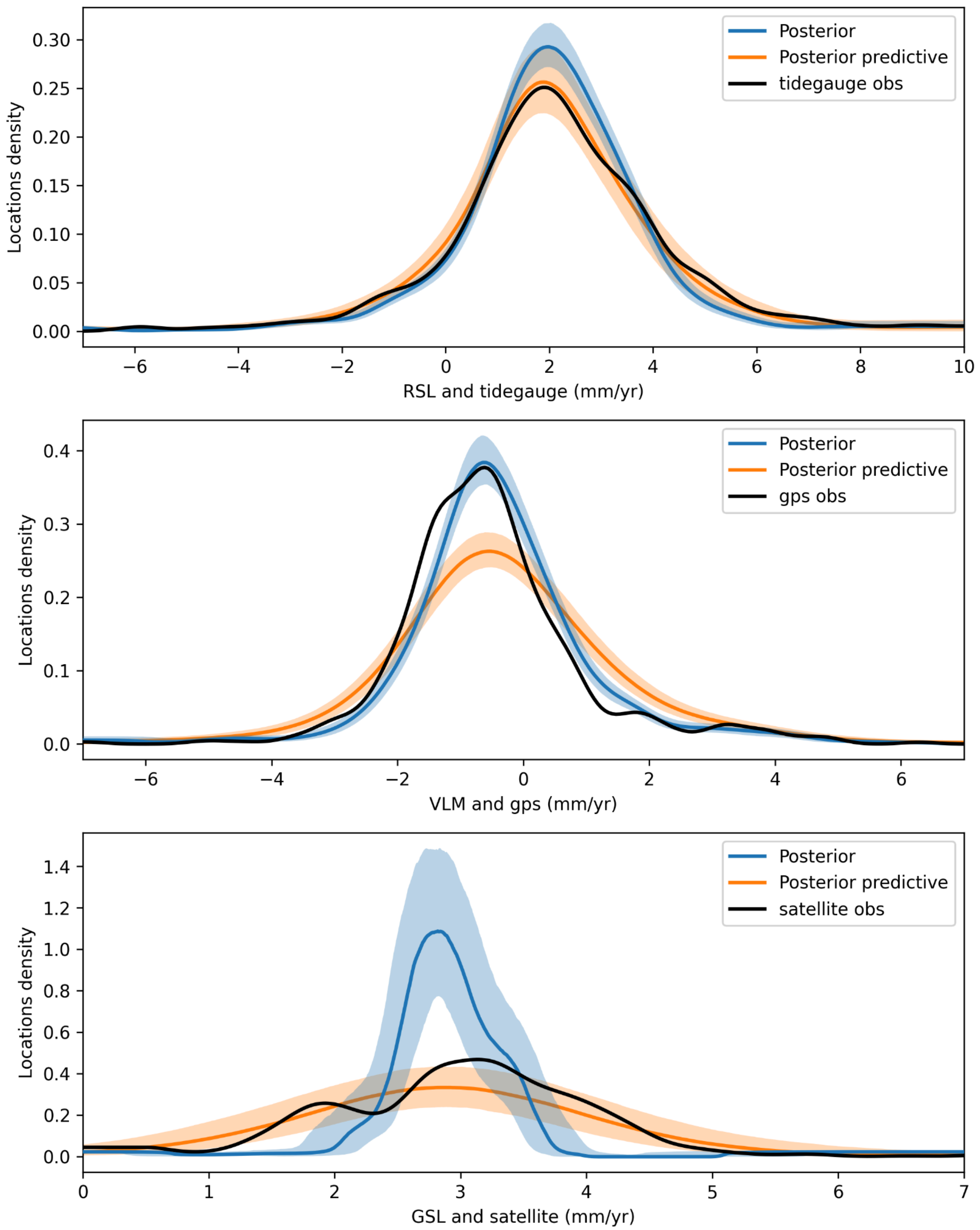

Fig. S12. Posterior, posterior predictive distributions and observational constraints across all tide gauge locations. The colored lines and shading depict the median and 90% range of the posterior and posterior predictive rate of the relative sea level contribution (A), vertical land motion (B) and geocentric sea level (C). Rate here refers to the mean rate of the respective observational coverage period following Fig. 3. The corresponding distribution of observational constraints is shown in black.

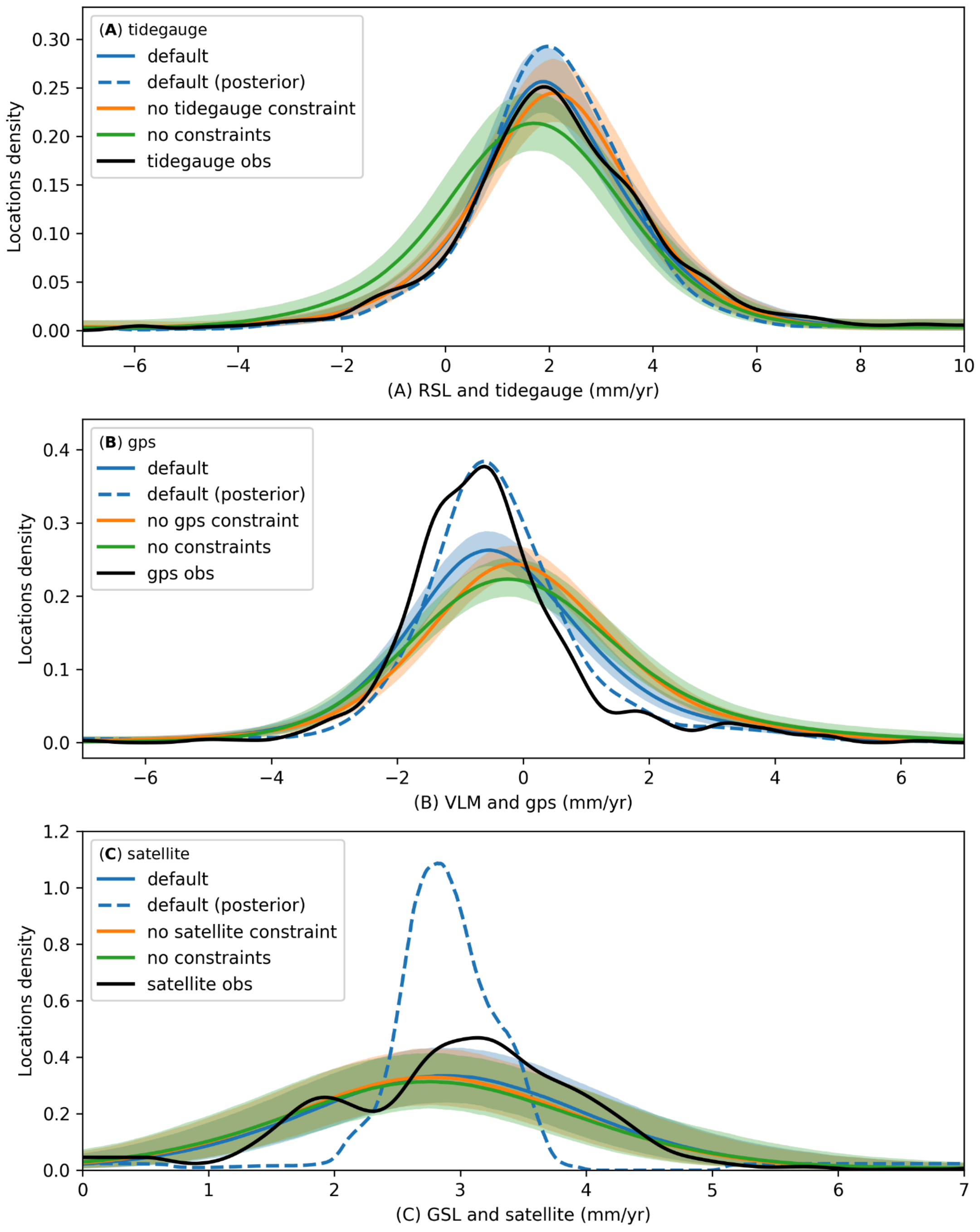

Fig. S13. Posterior predictive distributions and observational constraints across locations. The colored lines and shading depict the median and 90% range of the posterior predictive 1993-2018 rate of the relative sea level contribution (**A**), vertical land motion (**B**) and geocentric sea level (**C**). Rate here refers to the mean rate across the respective observational coverage period following Fig. 3. The corresponding distribution of observational constraints is shown in black. For each type of observation, three experiments are shown: the default experiment with all three constraints (blue), one experiment with the corresponding constraint removed (orange), and one experiment with no local constraints (green). For the default experiment, we also show the median distribution from the model posterior (dashed blue line) without the posterior predictive sampling step. We estimate median and 90% range from a Kernel Density Estimate fit performed across locations for each model realization.

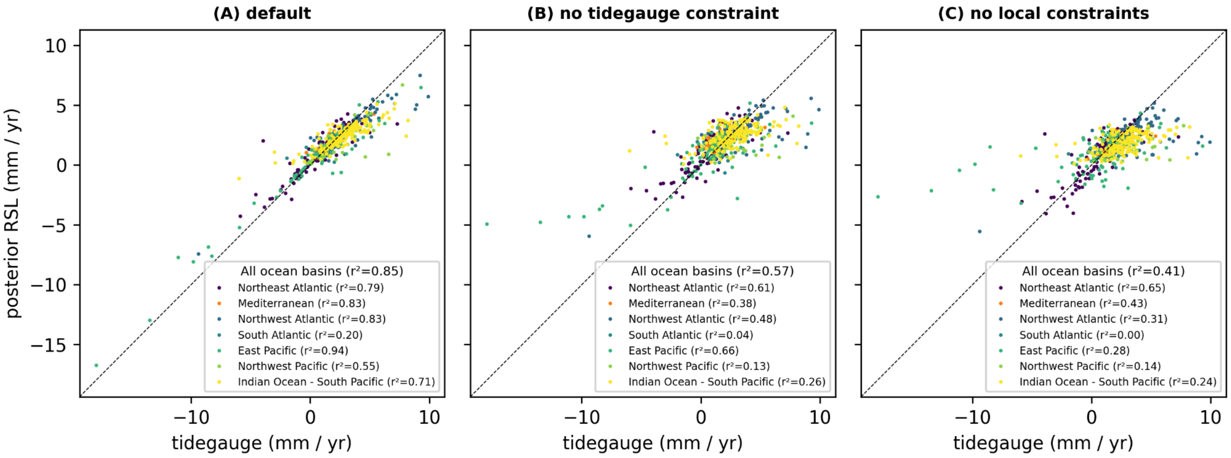

Fig. S14. Correlation between posterior relative sea level and observed tide-gauge trend per ocean basin. Default experiment (left panel), an experiment with only GPS and satellite altimetry constraint (center panel), and an experiment with all local constraints removed (right panel). Each dot represents the median rate for one tide-gauge location. The coefficient of determination r^2 is indicated in the legend for each ocean basin. Unforced natural variability is not included in the posterior trend.

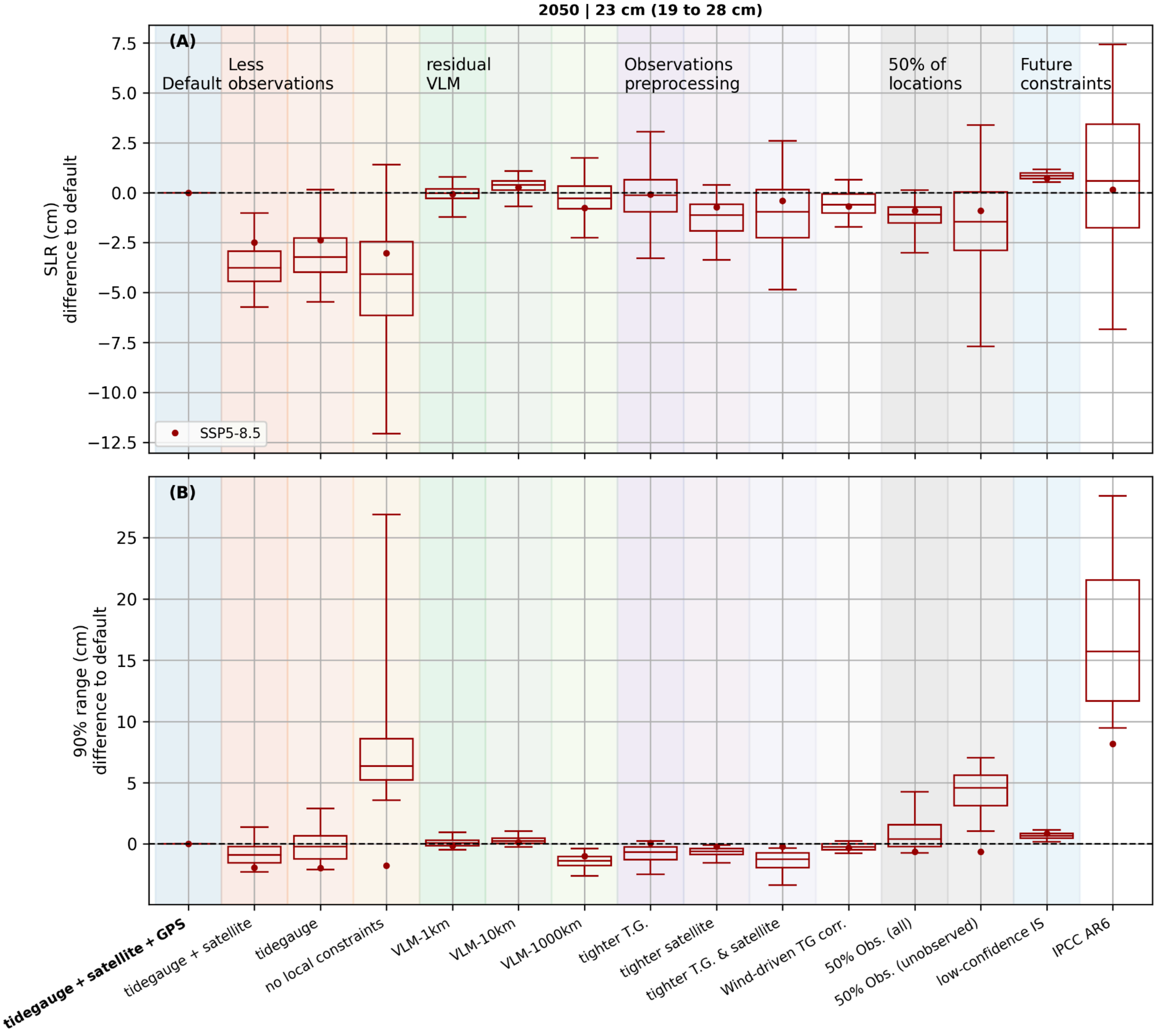

Fig. S15. Sensitivity experiments for local constraints. We show the median projections (A) and the 90% ranges (B) for the year 2050 in the SSP5-8.5 scenario, as a difference with respect to the default model. The distributions have been aggregated across locations as spatial percentiles (5th, 25th, 50th, 75th and 95th percentiles) displayed as whiskers. The global mean value is indicated as a dot. The experiments shown from left to right include such with leaving out complete observational datasets (red), with different VLM spatial correlation scales (green, 1km, 10km and 1000km instead of 100km default), assumption of tighter local errors (purple, 0.1mm/yr instead of their default value for the labeled component); an alternative wind correction following (*101*); leaving out all observational data from 50% of tide gauge stations (grey); and using the IPCC AR6 Table 9.9 (*1*) low confidence SSP5-8.5 ice sheet bounds for 2100 instead of the medium confidence SSP1-2.6 and SSP-5.8.5 bounds (blue). The 50% experiments show averages over 10 conducted experiments with randomly sampled locations. We show statistics that include all stations (labeled all), and one only over the stations that are not included as observations in the sample (labeled unobserved). The IPCC AR6 is also used for comparison (white).

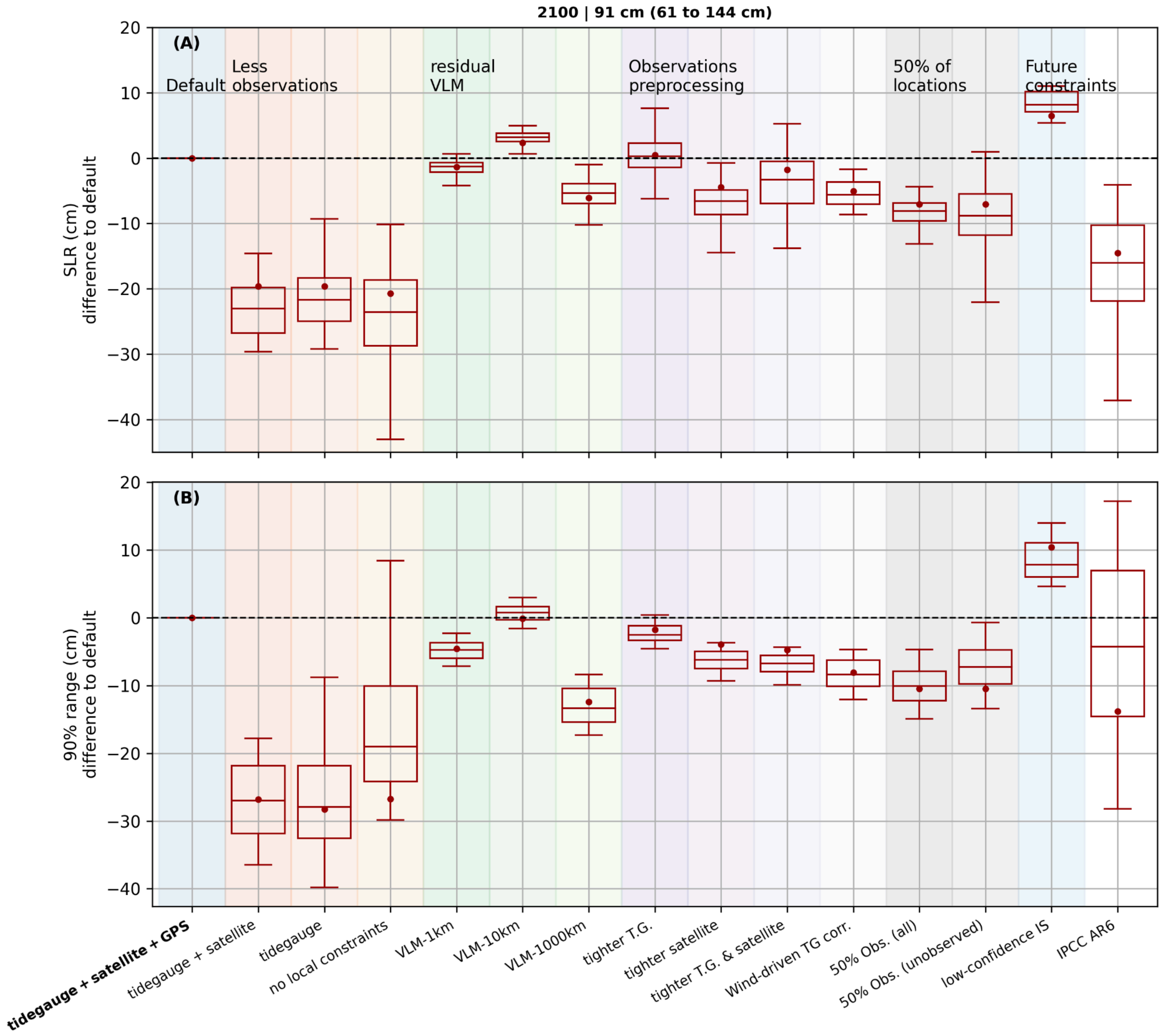

Fig. S16. Sensitivity experiments for local constraints. Same as fig. S15, but for 2100.

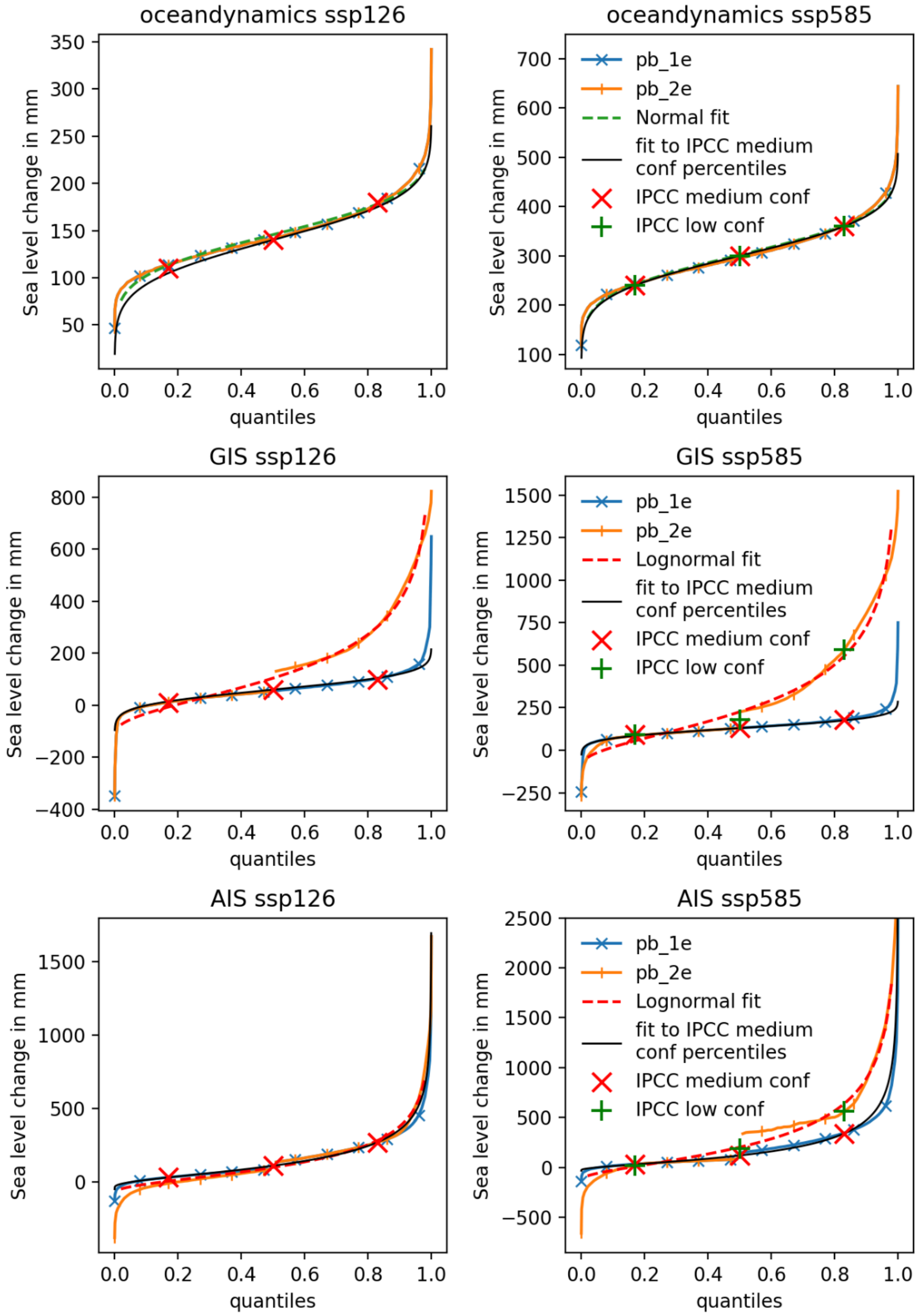

Fig. S17. Probability boxes of the IPCC and fits to IPCC percentiles. Probability boxes 1e (blue lines), which form the basis of the IPCC medium confidence projections and probability boxes 2e (yellow lines), which form the basis of the low confidence projections (*64*), the IPCC median and likely range (red crosses) of the medium confidence projections as reported in Table 9.8 (*1*), the IPCC median, 16.7th and 83.3th percentile (green pluses) of the low confidence projections from IPCC Table 9.9 (*1*), our fits to the median and likely range of medium confidence projections (black line), and normal and lognormal fits to the pboxes 2e (green and red hatched lines). We use the distributions fitted to the SSP1-2.6 and SSP5-8.5 medium confidence projections (normal

for thermal expansion and Greenland (GIS), and lognormal for Antarctica (AIS) as year 2100 constraints in our main results. We use the lognormal fits to the Greenland and Antarctica SSP5-8.5 contribution for the low confidence sensitivity experiment (see fig. S15, S16, S18). For glaciers, we use a more region-resolving approach, so glaciers is not shown here.

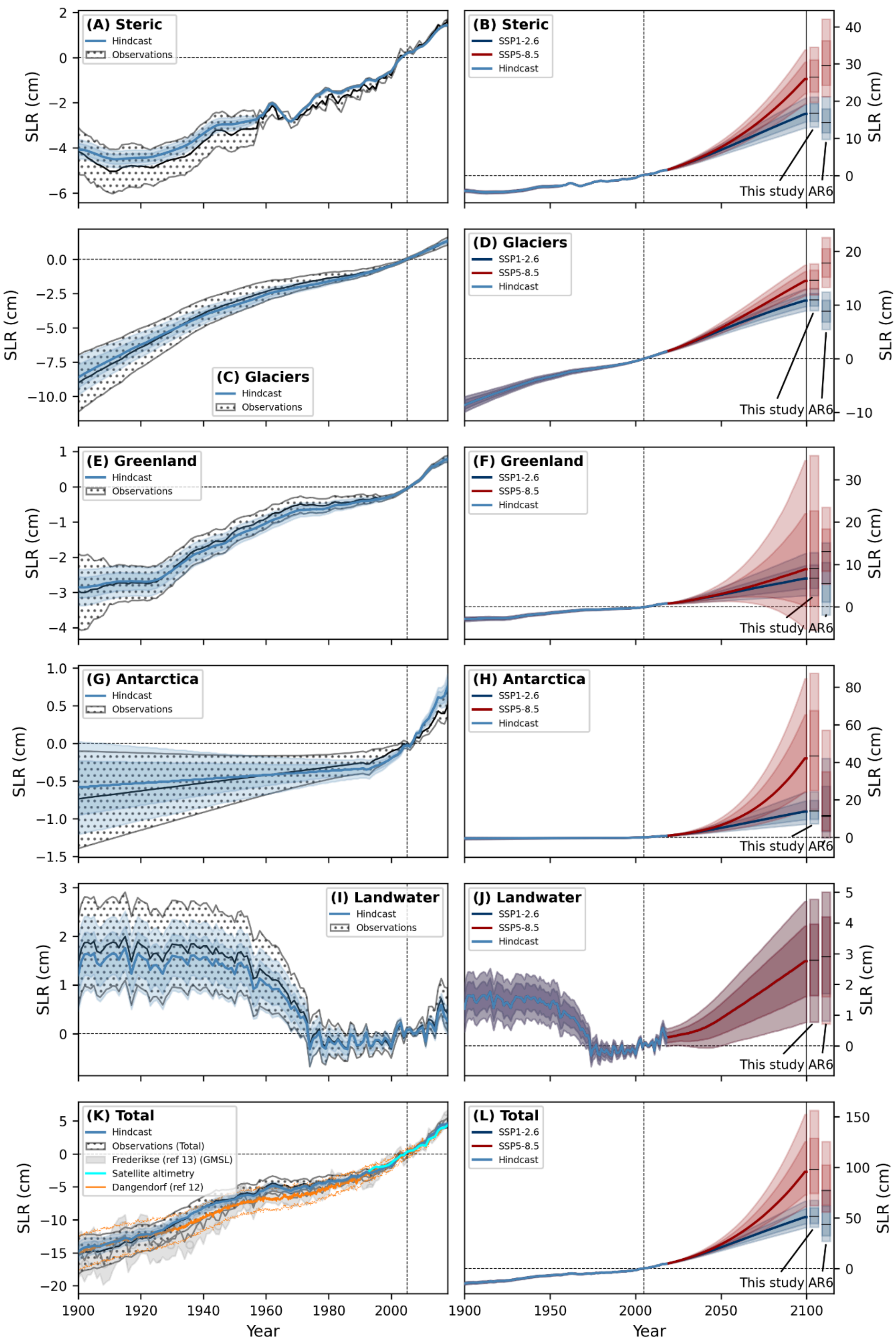

Fig. S18 Components of global sea level rise from 1900 to 2100 (low confidence sensitivity experiment). Like Fig. 2, but we use the IPCC AR6 WG1 low confidence estimates as constraints for 2100 (ref. (*1*) Table 9.9 last column). The AR6 bars at the very right shows the IPCC AR6 medium confidence estimates for comparison (not the low confidence estimates).

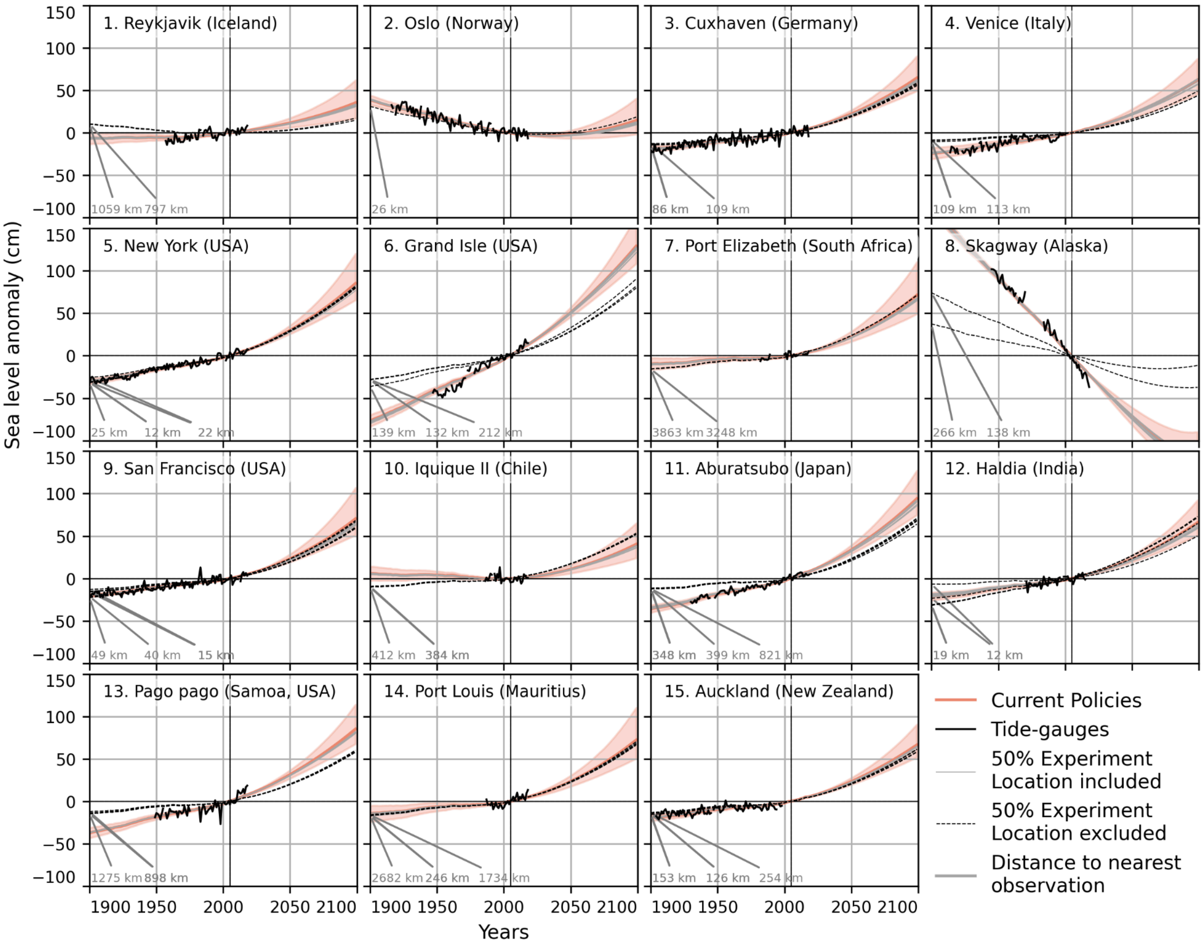

Fig. S19. Past and projected relative sea level change for the Current Policies pathway with observations of 50% randomly chosen tide gauge locations left out. Red line and shading show the median and 90% range of our default projections for the IPCC AR6 WG3 Current Policies pathway. The 50% exclusion experiments are shown as full, grey line when the specific location shown is observed (included) or as black dashed line when it is unobserved (excluded). We additionally indicate the distance to the nearest observation included (annotations within panels in km).

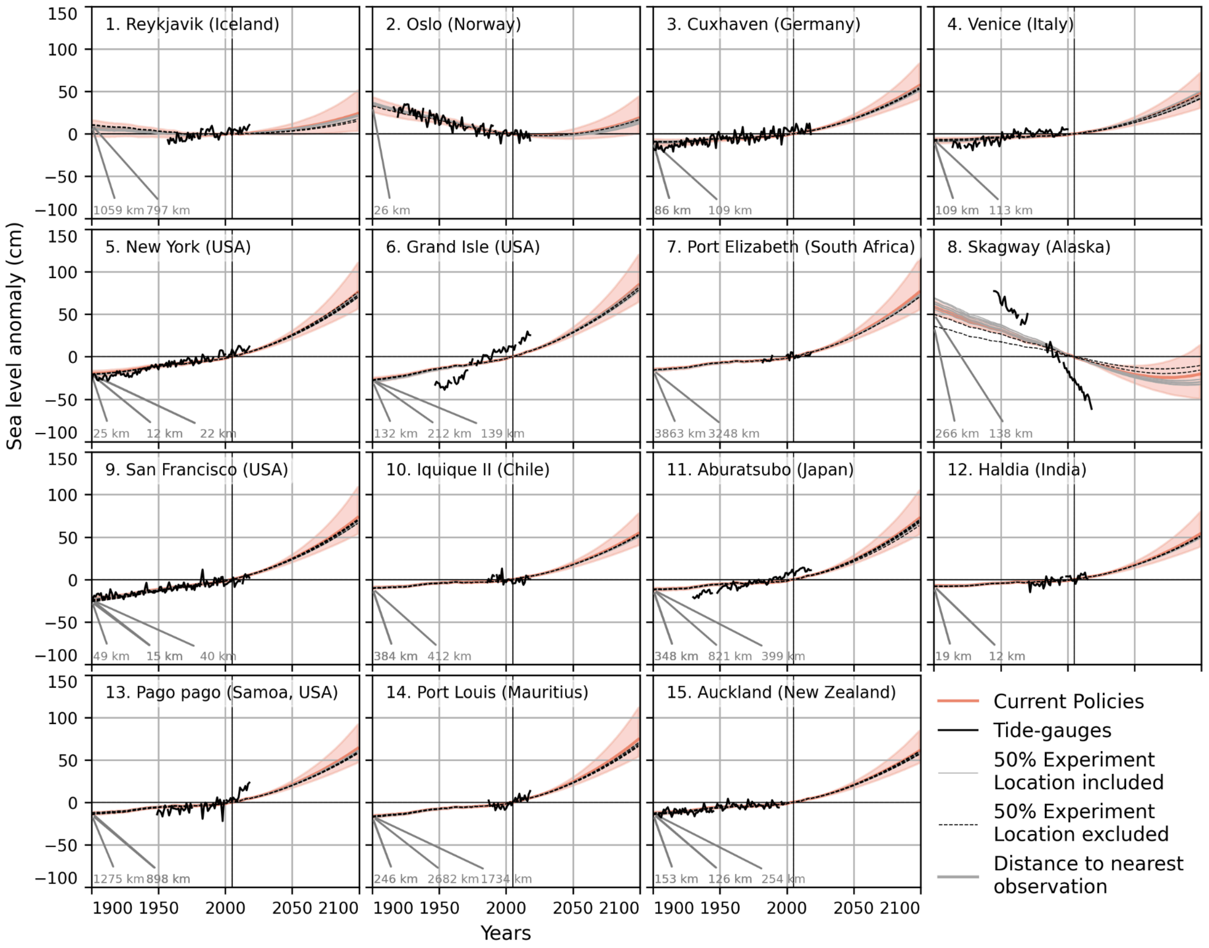

Fig. S20. Like fig. S19, but without the residual VLM contribution.

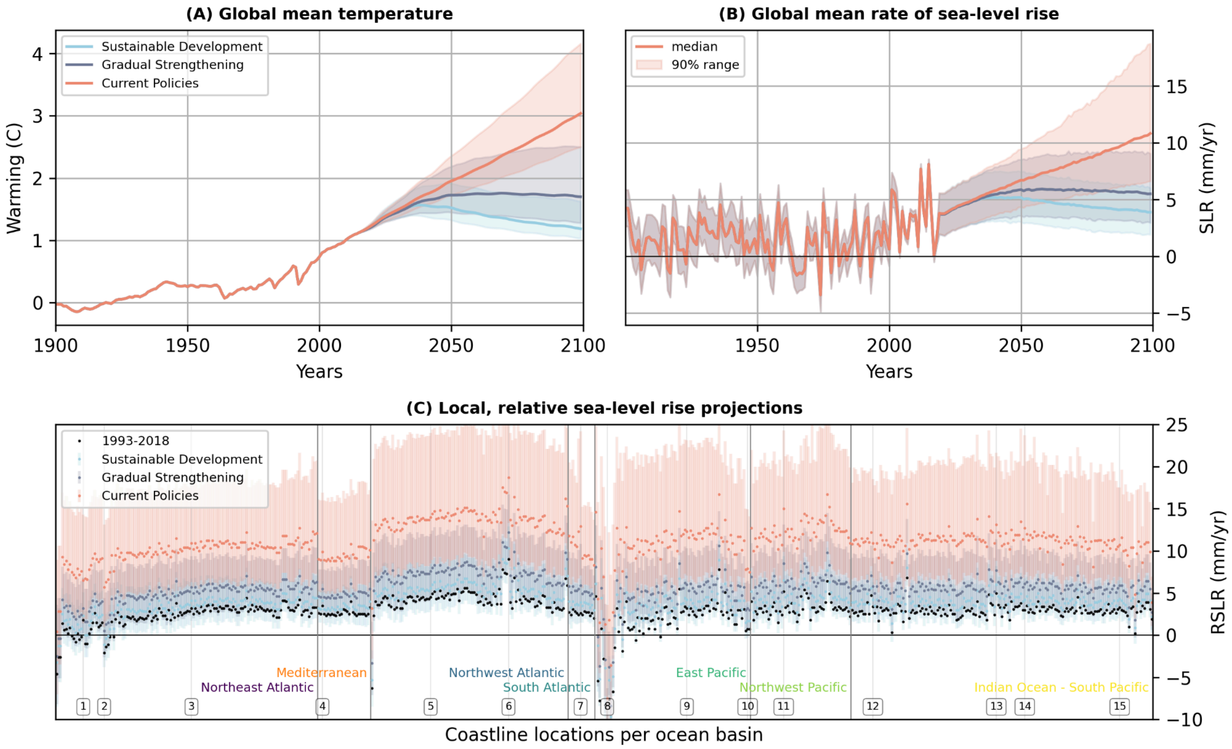

Fig. S21. Past and projected sea level change for three IPCC AR6 WG3 scenarios including temperature uncertainty. Like Fig. 4 but with temperature uncertainty using the temperature ensemble from (*57*)

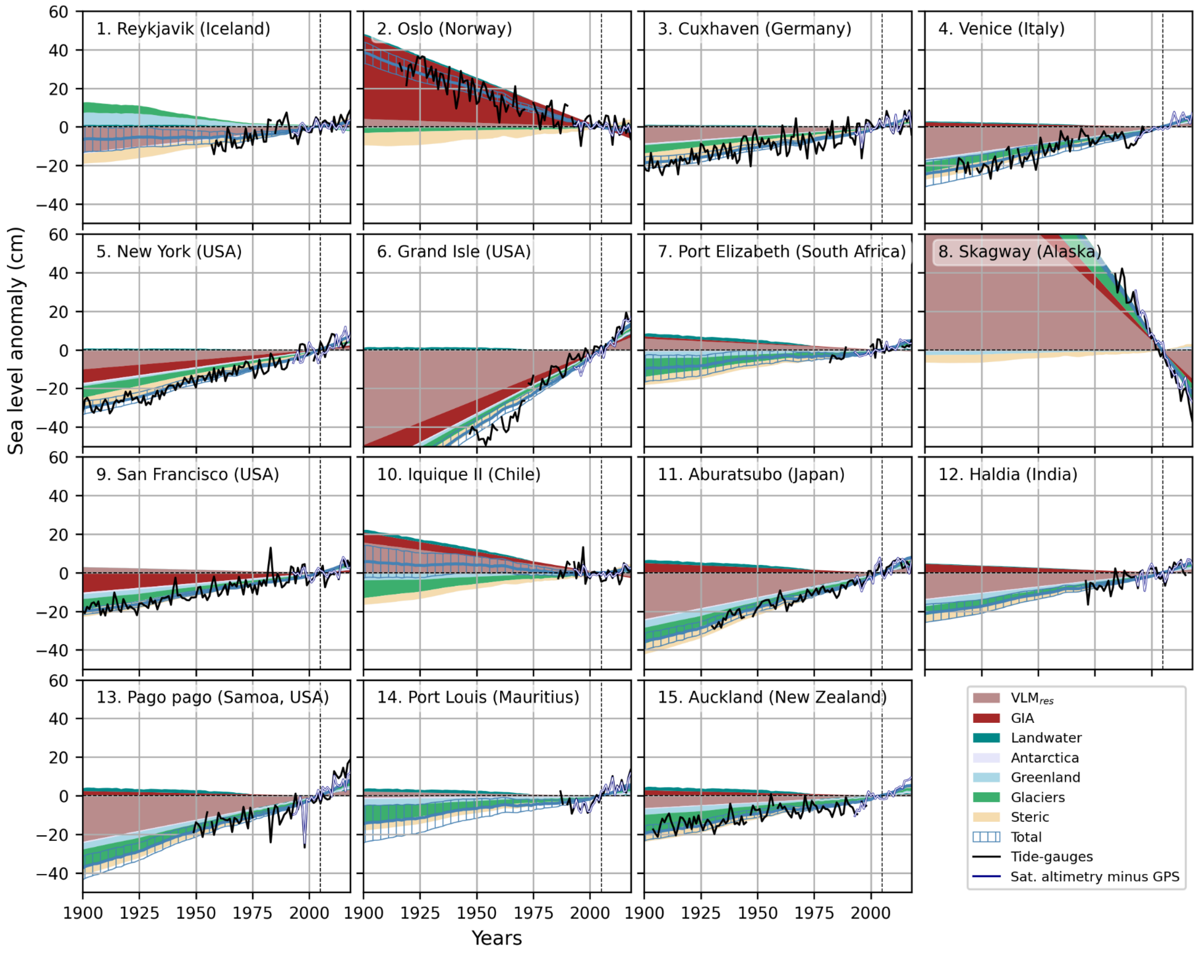

Fig. S22. Past relative sea level rise and its components at selected tide-gauge locations. Like Fig. 5, but for the historical period only.

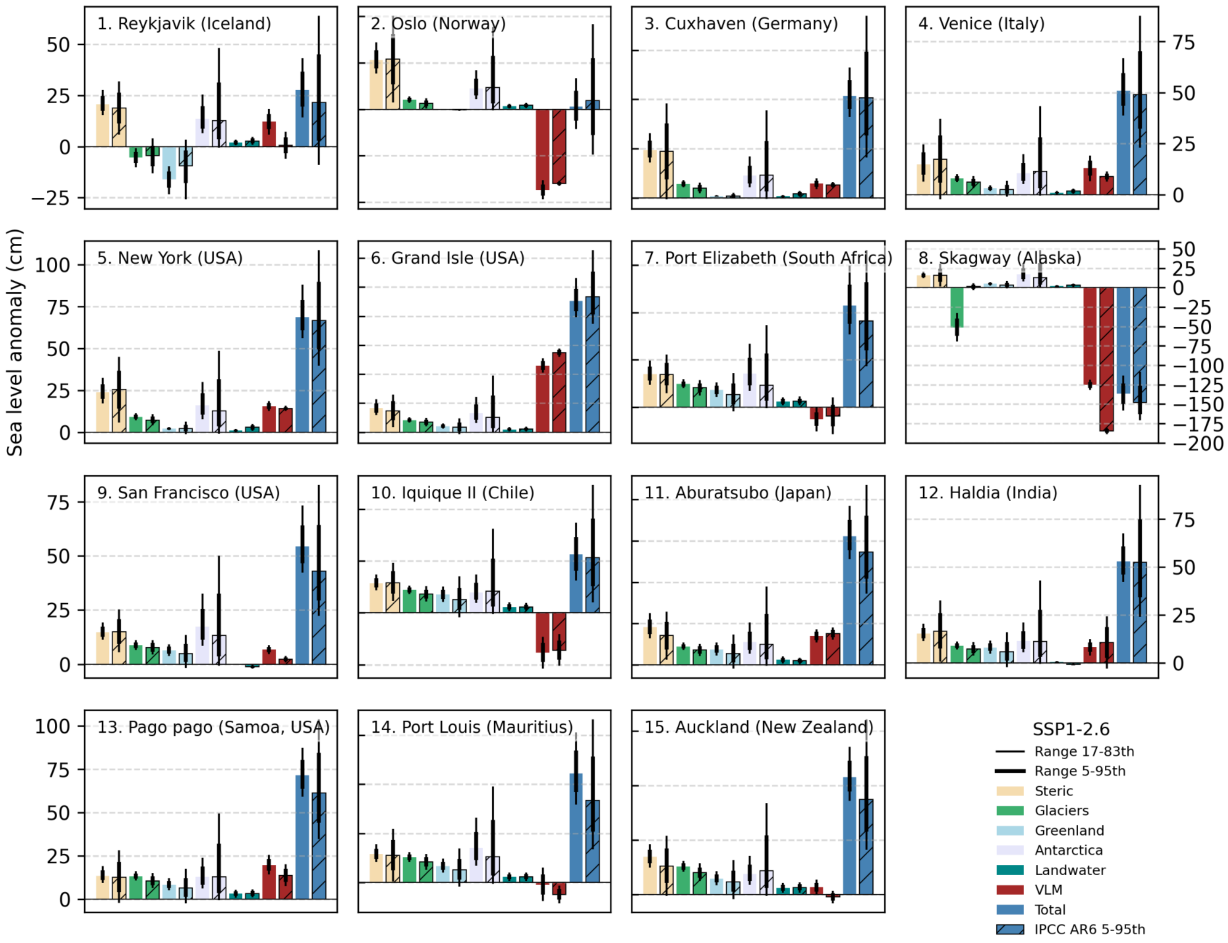

Fig. S23. Comparison of components and total relative sea level rise by 2100 for the 15 example locations of Fig. 4-6 for the SSP1-2.6 scenario. Colored bars show our model output, hatched bars with the same color the respective IPCC contribution taken from (*64*). We show the 67% range as thick black line and the 90% range as thin black line. Distance between y-axis ticks is 25cm with black horizontal line indicating zero.

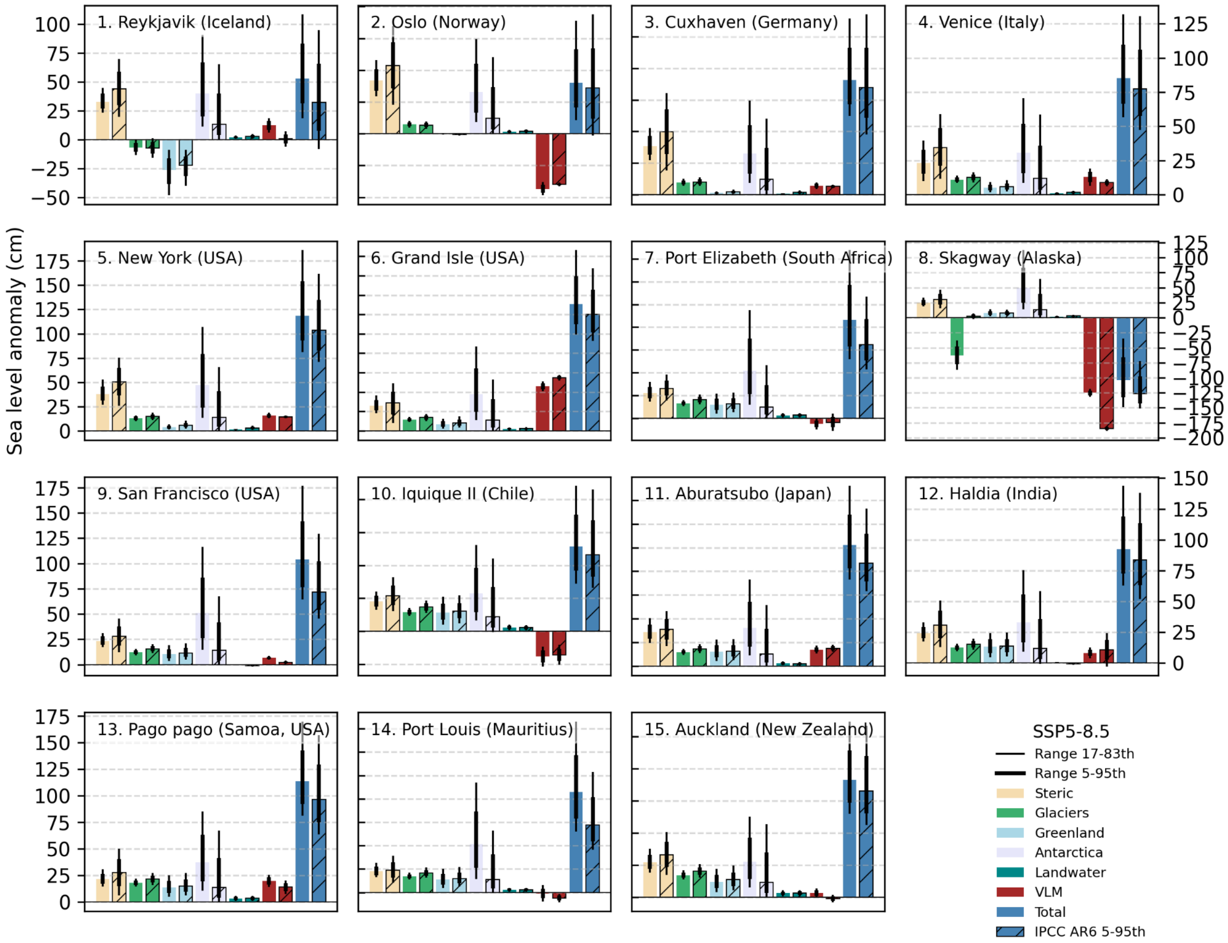

Fig. S24. Comparison of components and total relative sea level rise by 2100 for the 15 example locations of Fig. 4-6 for the SSP5-8.5 scenario. Colored bars show our model output, hatched bars with the same color the respective IPCC contribution taken from (*64*). We show the 67% range as thick black line and the 90% range as thin black line. Distance between y-axis ticks is 25cm with black horizontal line indicating zero.

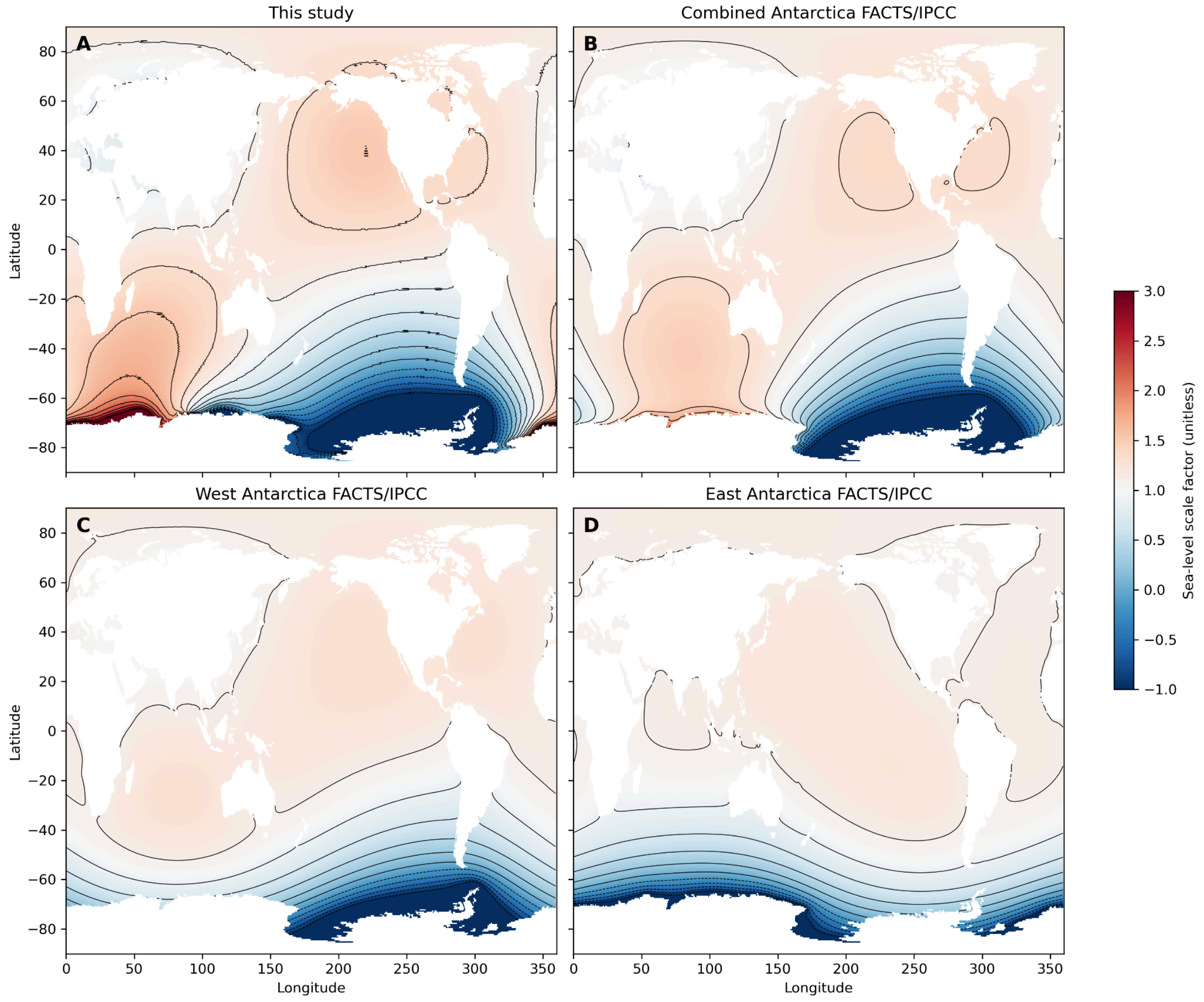

Fig. S25 Antarctic ice sheet fingerprint applied in this study as compared to IPCC AR6 / FACTS. We apply in this study a single fingerprint for Antarctica (A) based on observations (*13*), in contrast to the IPCC framework based on FACTS, which applies separate fingerprints. We show the best fit (B) of the IPCC combination of the West Antarctica (C) and East Antarctica (D) fingerprints. The best fit is X=1.46 for X*West + (1-X)*East. FACTS fingerprints taken from (*102*) are assumed to not have changed since applied for the IPCC regional projections.

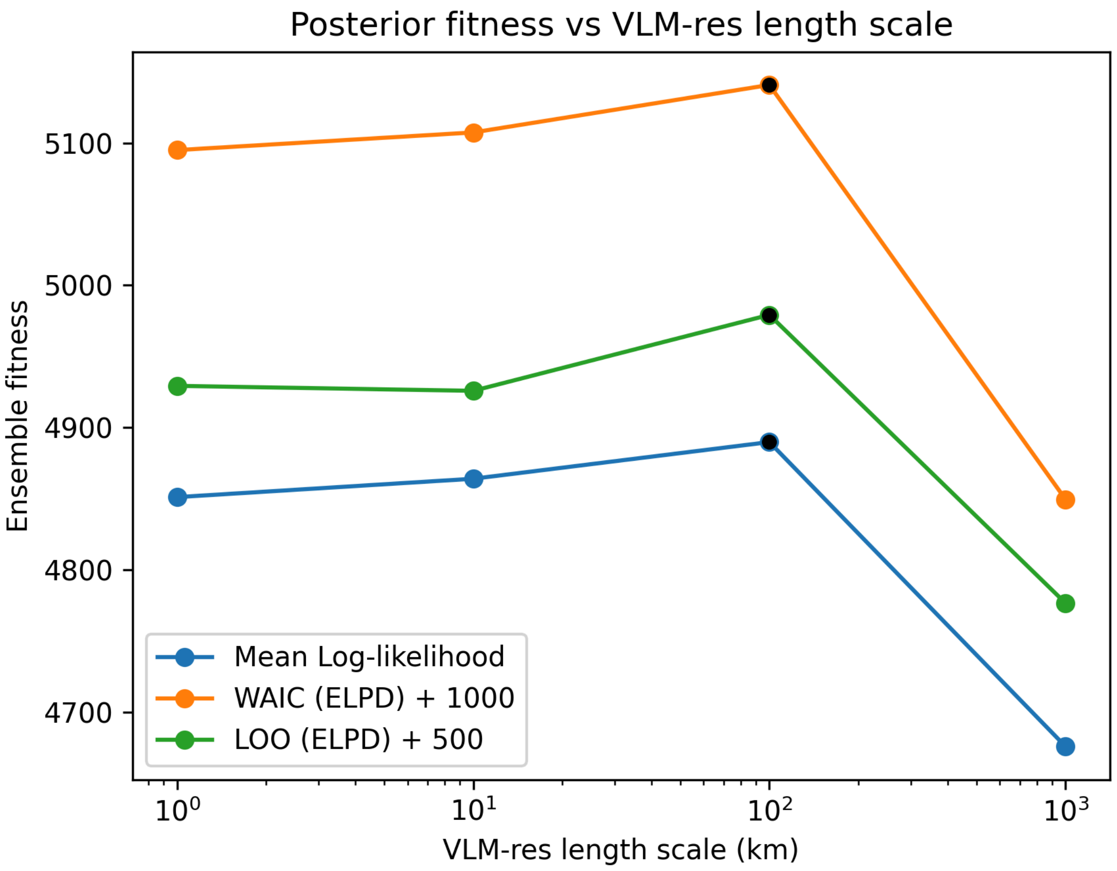

Fig. S26. Measures of model fit for different spatial correlation lengths for residual vertical land motion. Model comparison metrics include: Mean log-likelihood (average log probability of observed data under the posterior predictive distribution), WAIC (ELPD) (expected log pointwise predictive density using the Widely Applicable Information Criterion), and LOO (ELPD) (expected log pointwise predictive density using approximate leave-one-out cross-validation). All metrics are reported in log units, with higher values indicating better predictive performance. Differences should be interpreted in relation to their respective standard errors. We test the sensitivity for correlation scales 1km, 10km, 100km and 1000km. Our default is 100km has the best performance. The log-likelihood mean is across chains and draws.

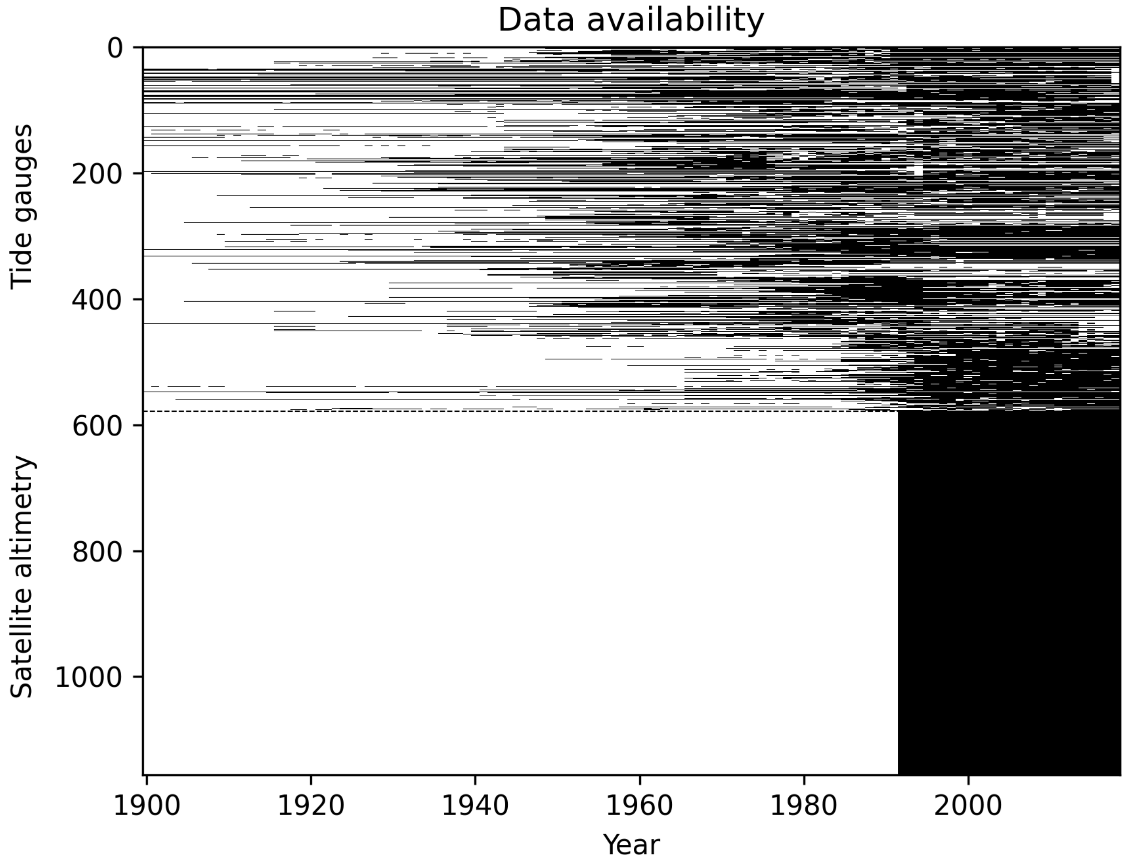

Fig. S27. Tide gauges and satellite altimetry data availability. The black areas indicate that data are available. This underlines the sampling mask applied to the variance-corrected CMIP6 ensemble when estimating trend error covariances. For satellite altimetry, this is between 1993 and 2019 (this figure only goes until 2018). The ordering on the y axis is consistent with fig. S31 and Fig. 3.

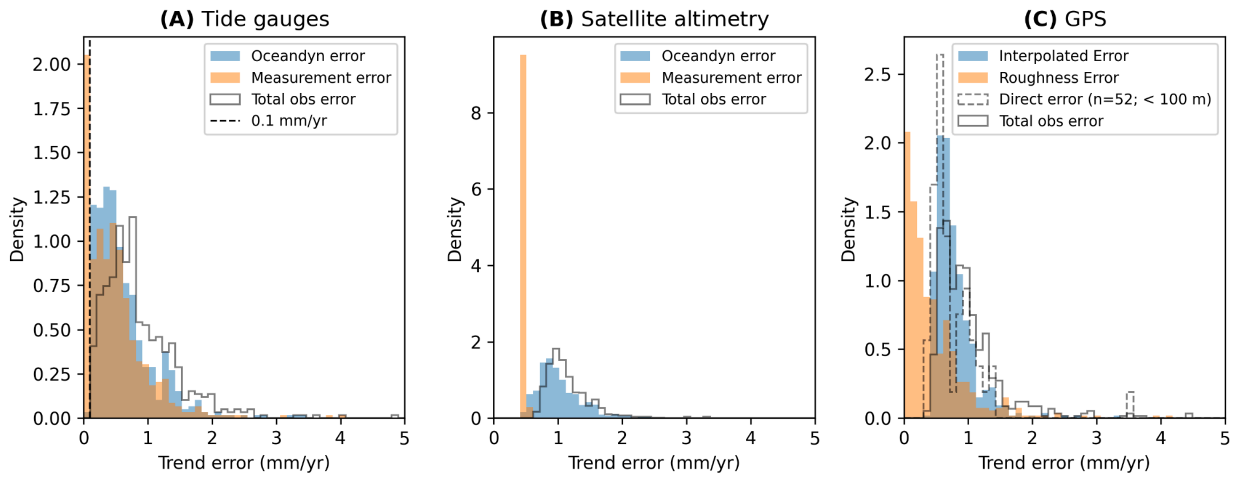

Fig. S28. Breakup of observation error into its various components for tide gauges (A), satellite altimetry (B) and GPS (C) observation errors. The distributions are over all tide-gauge locations. The "oceandyn" error in tide gauges and satellite altimetry is calculated from variance-corrected CMIP6 pre-industrial control runs. The "measurement error" in tide-gauges is calculated by scaling up the oceandyn error according to the annual ratio between tide-gauge and satellite variance. The "measurement error" in satellite altimetry is calculated according to (*53*). For most stations, the GPS error is the sum of error interpolated from nearby GPS stations, and a "roughness" error calculated as the difference between the smooth GPS field by (*43*), and the actual GPS trend (see details in Methods). Stations with a co-located GPS station (52 locations with GPS station less than 100m from the tide gauge) use the formal GPS trend error ("direct error") instead. In all panels the "total obs error" histogram shows the final, resulting error used in this work.

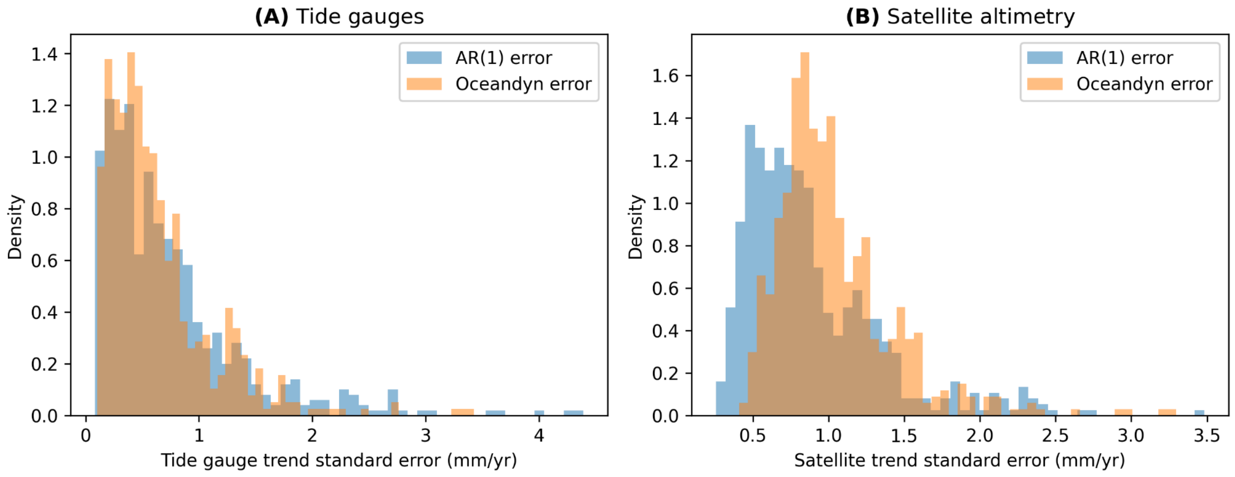

Fig. S29. Comparison between trend errors calculated from CMIP6-derived ocean dynamic and a simple AR(1) for tide gauges (A) and satellite altimetry (B). While the order of magnitude is generally similar for the tide gauges, using a simple AR(1) model instead of our more sophisticated approach with CMIP6 would result in notable underestimation of the point-wise trend error.

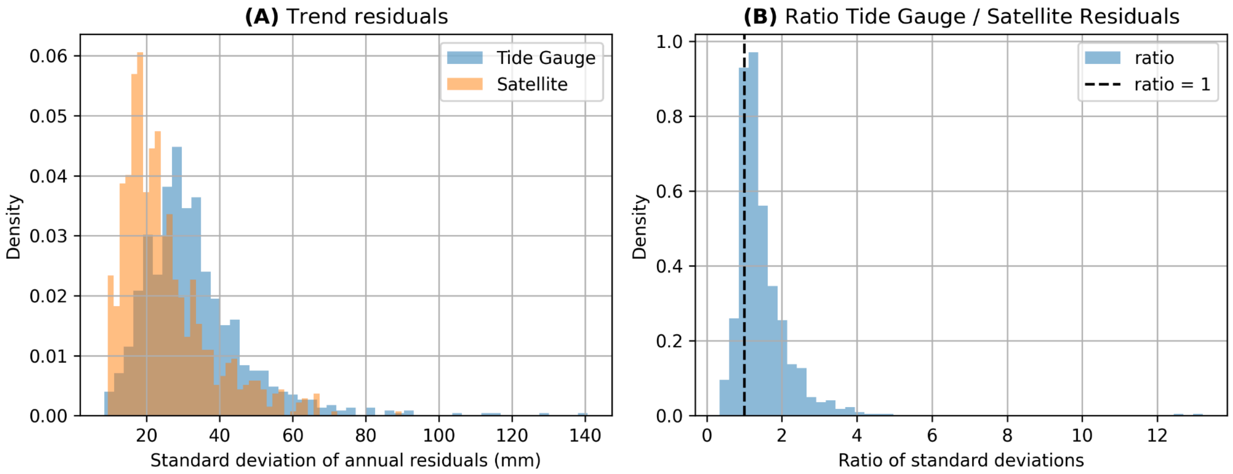

Fig. S30. Comparison between annual residuals from tide-gauge and satellite altimetry records. Annual residuals were obtained by fitting a linear trend to local records, and their standard deviation is shown here. The standard deviation tends to be larger for tide-gauge residuals compared to satellite residuals, as seen by their respective spatial distributions (A) and by the distribution of their ratio (B), and is generally larger than one. The ratio, when larger than one, is used to scale up tide-gauge error due to unforced ocean variability, and the excess error is attributed to locally-independent measurement error.

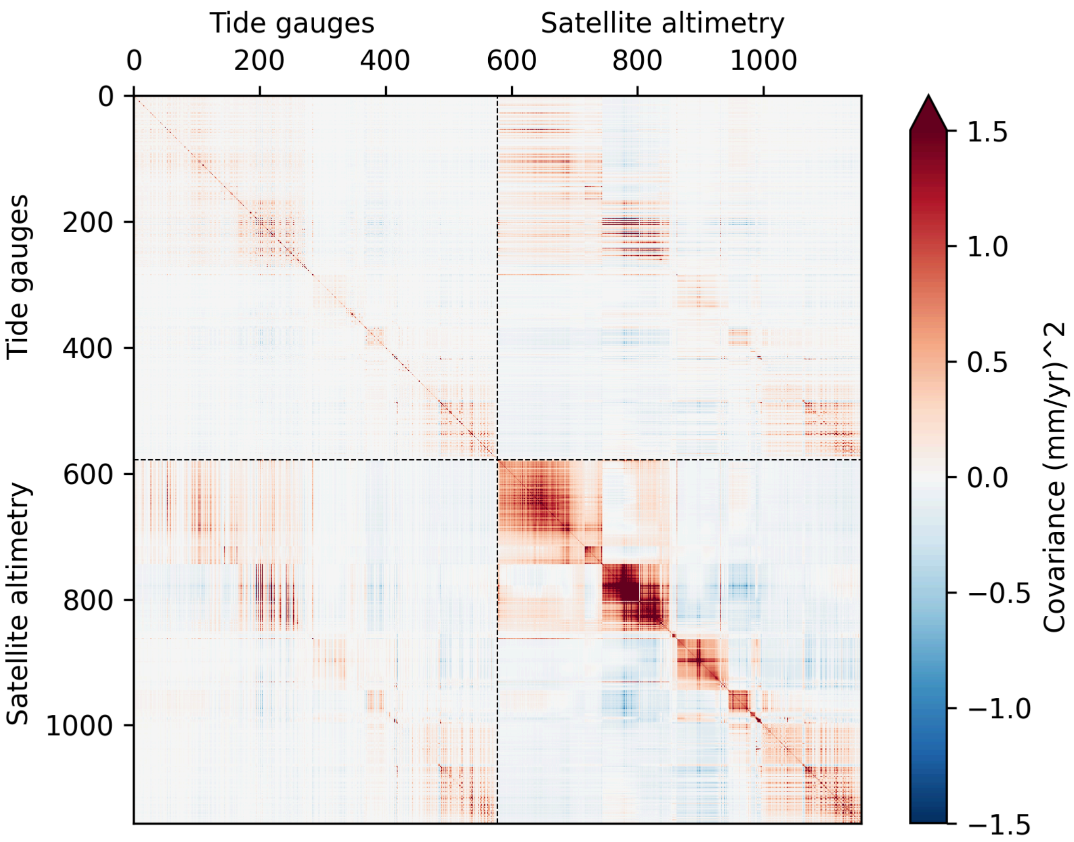

Fig. S31. Spatial covariance within and between tide gauges and satellite altimetry trend errors. The dashed line separates the values into spatial covariances within tide gauges (upper left), within satellite altimetry measurements (lower right), and across tide gauges and satellite altimetry measurements (lower left and upper right). All errors are included (the diagonal corresponds to "total obs error" in fig. S28 A, B). The locations are sorted according to ocean basin and latitude, like Fig. 3. Negative values indicate anticorrelation.

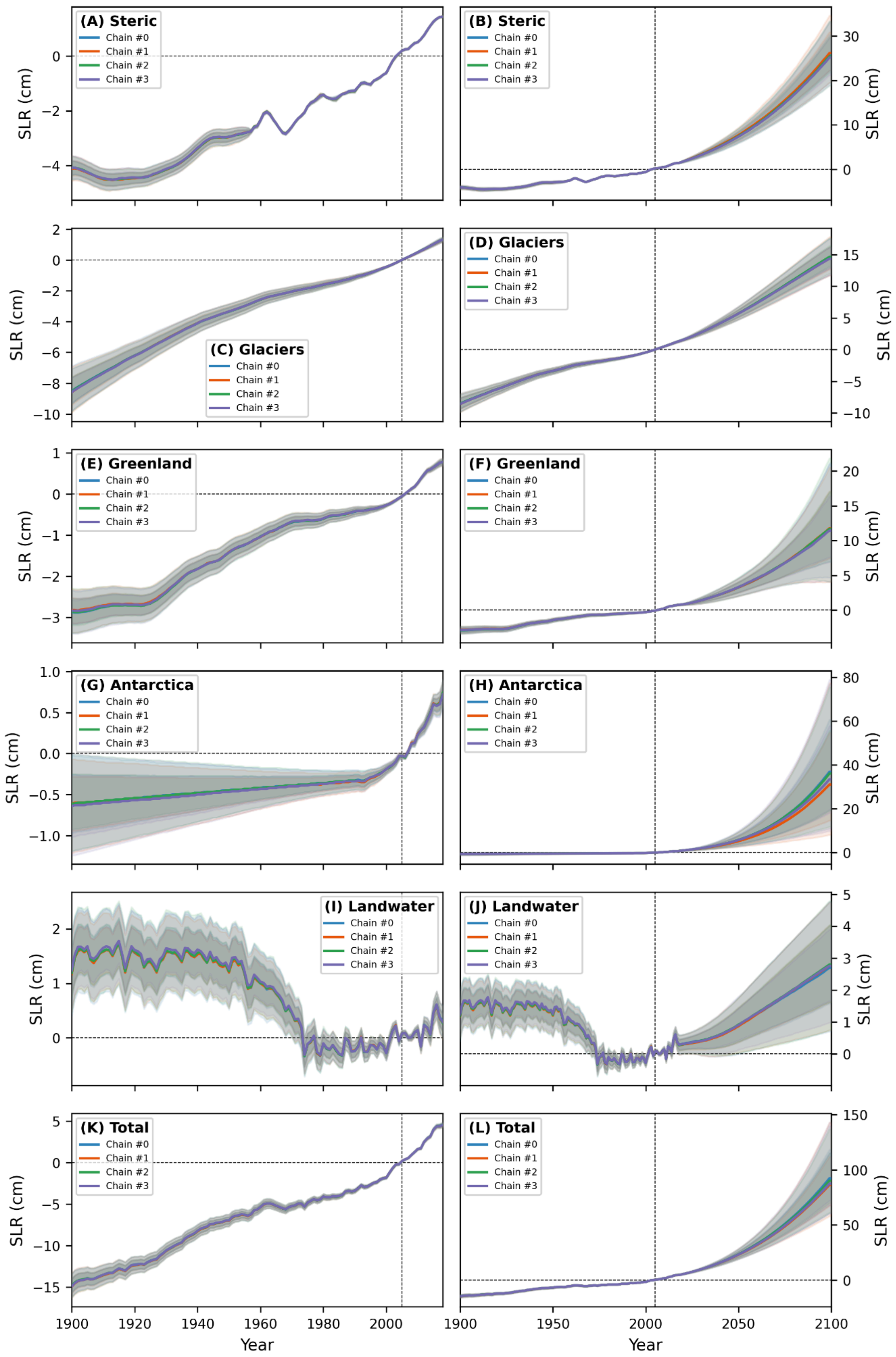

Fig. S32. Convergence diagnostics via individual sampling chains. Future sea-level projections in the SSP5-8.5 scenario with the posterior ensemble, shown for the four chains individually.

REFERENCES AND NOTES

1. B. Fox-Kemper, B. H. T. Hewitt, C. Xiao, G. Aðalgeirsdóttir, S. S. Drijfhout, T. L. Edwards, N. R. Golledge, M. Hemer, R. E. Kopp, G. Krinner, A. Mix, D. Notz, S. Nowicki, I. S. Nurhati, L. Ruiz, J.-B. Sallée, A. B. A. Slangen, Y. Yu, Ocean, Cryosphere and Sea Level Change, in Climate Change 2021: The Physical Science Basis. *Contribution of Working Group I to the Sixth Assessment Report of the Intergovernmental Panel on Climate Change*, V. Masson-Delmotte, P. Zhai, A. Pirani, S. L. Connors, C. Péan, S. Berger, N. Caud, Y. Chen, L. Goldfarb, M.I. Gomis, M. Huang, K. Leitzell, E. Lonnoy, J. B. R. Matthews, T. K. Maycock, T. Waterfield, O. Yelekçi, R. Yu, B. Zhou, Eds. (Cambridge Univ. Press, 2021), pp. 1211–1362.

2. IPCC, *The Ocean and Cryosphere in a Changing Climate: Special Report of the Intergovernmental Panel on Climate Change* (Cambridge Univ. Press, 2019).

3. IPCC, *Climate Change 2022 - Impacts, Adaptation and Vulnerability: Working Group II Contribution to the Sixth Assessment Report of the Intergovernmental Panel on Climate Change* (Cambridge Univ. Press, 2022).

4. R. J. Nicholls, S. E. Hanson, J. A. Lowe, R. A. Warrick, X. Lu, A. J. Long, Sea-level scenarios for evaluating coastal impacts. *Wiley Interdiscip. Rev. Clim. Change* **5**, 129–150 (2014).

5. R. E. Kopp, C. C. Hay, C. M. Little, J. X. Mitrovica, Geographic variability of sea-level change. *Curr. Clim. Change Rep.* **1**, 192–204 (2015).

6. J. M. Gregory, S. M. Griffies, C. W. Hughes, J. A. Lowe, J. A. Church, I. Fukimori, N. Gomez, R. E. Kopp, F. Landerer, G. L. Cozannet, R. M. Ponte, D. Stammer, M. E. Tamisiea, R. S. W. van de Wal, Concepts and terminology for sea level: Mean, variability and change, both local and global. *Surv. Geophys.* **40**, 1251–1289 (2019).

7. A. Wyns, COP27 establishes loss and damage fund to respond to human cost of climate change. *Lancet Planet Health* **7**, e21–e22 (2023).

8. J. Hinkel, J. C. J. H. Aerts, S. Brown, J. A. Jiménez, D. Lincke, R. J. Nicholls, P. Scussolini, A. Sanchez-Arcilla, A. Vafeidis, K. A. Addo, The ability of societies to adapt to twenty-first-century sea-level rise. *Nat. Clim. Change* **8**, 570–578 (2018).

9. J. Lawrence, R. Bell, P. Blackett, S. Stephens, S. Allan, National guidance for adapting to coastal hazards and sea-level rise: Anticipating change, when and how to change pathway. *Environ. Sci. Policy* **82**, 100–107 (2018).

10. R. J. Nicholls, S. E. Hanson, J. A. Lowe, A. B. A. Slangen, T. Wahl, J. Hinkel, A. J. Long, Integrating new sea-level scenarios into coastal risk and adaptation assessments: An ongoing process. *Wiley Interdiscip. Rev. Clim. Change* **12**, e706 (2021).

11. J. A. Church, N. J. White, L. F. Konikow, C. M. Domingues, J. G. Cogley, E. Rignot, J. M. Gregory, M. R. van den Broeke, A. J. Monaghan, I. Velicogna, Revisiting the Earth's sea-level and energy budgets from 1961 to 2008. *Geophys. Res. Lett.* **38**, L18601 (2011).

12. S. Dangendorf, C. Hay, F. M. Calafat, M. Marcos, C. G. Piecuch, K. Berk, J. Jensen, Persistent acceleration in global sea-level rise since the 1960s. *Nat. Clim. Change* **9**, 705–710 (2019).

13. T. Frederikse, F. Landerer, L. Caron, S. Adhikari, D. Parkes, V. W. Humphrey, S. Dangendorf, P. Hogarth, L. Zanna, L. Cheng, Y.-H. Wu, The causes of sea-level rise since 1900. *Nature* **584**, 393–397 (2020).

14. S. Dangendorf, Q. Sun, T. Wahl, P. Thompson, J. X. Mitrovica, B. Hamlington, Probabilistic reconstruction of sea-level changes and their causes since 1900. *Earth Syst. Sci. Data* **16**, 3471–3494 (2024).

15. A. J. Garner, J. L. Weiss, A. Parris, R. E. Kopp, R. M. Horton, J. T. Overpeck, B. P. Horton, Evolution of 21st century sea level rise projections. *Earths Future* **6**, 1603–1615 (2018).

16. S. Jevrejeva, T. Frederikse, R. E. Kopp, G. le Cozannet, L. P. Jackson, R. S. W. van de Wal, Probabilistic sea level projections at the coast by 2100. *Surv. Geophys.* **40**, 1673–1696 (2019).

17. A. B. A. Slangen, M. D. Palmer, C. M. L. Camargo, J. A. Church, T. L. Edwards, T. H. J. Hermans, H. T. Hewitt, G. G. Garner, J. M. Gregory, R. E. Kopp, V. M. Santos, R. S. W. van de Wal, The evolution of 21st century sea-level projections from IPCC AR5 to AR6 and beyond. *Camb. Prism. Coast. Futures* **1**, e7 (2023).

18. R. E. Kopp, R. M. Horton, C. M. Little, J. X. Mitrovica, M. Oppenheimer, D. J. Rasmussen, B. H. Strauss, C. Tebaldi, Probabilistic 21st and 22nd century sea-level projections at a global network of tide-gauge sites. *Earths Future* **2**, 383–406 (2014).

19. L. P. Jackson, S. Jevrejeva, A probabilistic approach to 21st century regional sea-level projections using RCP and high-end scenarios. *Glob. Planet. Change* **146**, 179–189 (2016).

20. A. Grinsted, S. Jevrejeva, R. E. M. Riva, D. Dahl-Jensen, Sea level rise projections for northern Europe under RCP8.5. *Climate Res.* **64**, 15–23 (2015).

21. M. D. Palmer, J. M. Gregory, M. Bagge, D. Calvert, J. M. Hagedoorn, T. Howard, V. Klemann, J. A. Lowe, C. D. Roberts, A. B. A. Slangen, G. Spada, Exploring the drivers of global and local sea-level change over the 21st century and beyond. *Earths Future* **8**, e2019EF001413 (2020).

22. M. Haasnoot, J. H. Kwakkel, W. E. Walker, J. ter Maat, Dynamic adaptive policy pathways: A method for crafting robust decisions for a deeply uncertain world. *Glob. Environ. Change* **23**, 485–498 (2013).

23. N. Ranger, T. Reeder, J. Lowe, Addressing ‘deep’ uncertainty over long-term climate in major infrastructure projects: Four innovations of the Thames Estuary 2100 Project. *EURO J. Decis. Process.* **1**, 233–262 (2013).

24. B. D. Hamlington, D. P. Chambers, T. Frederikse, S. Dangendorf, S. Fournier, B. Buzzanga, R. S. Nerem, Observation-based trajectory of future sea level for the coastal United States tracks near high-end model projections. *Commun. Earth Environ.* **3**, 230 (2022).

25. N. A. Cradock-Henry, N. Kirk, S. Ricart, G. Diprose, R. Kannemeyer, Decisions, options, and actions in the face of uncertainty: A systematic bibliometric and thematic review of climate adaptation pathways. *Environ. Res. Lett.* **18**, 073002 (2023).

26. A. Martínez-Asensio, G. Wöppelmann, V. Ballu, M. Becker, L. Testut, A. K. Magnan, V. K. E. Duvat, Relative sea-level rise and the influence of vertical land motion at Tropical Pacific Islands. *Glob. Planet. Change* **176**, 132–143 (2019).

27. M. Shirzaei, J. Freymueller, T. E. Törnqvist, D. L. Galloway, T. Dura, P. S. J. Minderhoud, Measuring, modelling and projecting coastal land subsidence. *Nat. Rev. Earth Environ.* **2**, 40–58 (2021).

28. M. Mengel, A. Nauels, J. Rogelj, C.-F. Schleussner, Committed sea-level rise under the Paris Agreement and the legacy of delayed mitigation action. *Nat. Commun.* **9**, 601 (2018).

29. A. Nauels, J. Gütschow, M. Mengel, M. Meinshausen, P. U. Clark, C. F. Schleussner, Attributing long-term sea-level rise to Paris Agreement emission pledges. *Proc. Natl. Acad. Sci. U.S.A.* **116**, 23487–23492 (2019).

30. C.-F. Schleussner, T. K. Lissner, E. M. Fischer, J. Wohland, M. Perrette, A. Golly, J. Rogelj, K. Childers, J. Schewe, K. Frieler, M. Mengel, W. Hare, M. Schaeffer, Differential climate impacts for policy-relevant limits to global warming: The case of 1.5°C and 2°C. *Earth Syst. Dynam.* **7**, 327–351 (2016).

31. IPCC, *Climate Change 2022 - Mitigation of Climate Change: Working Group III Contribution to the Sixth Assessment Report of the Intergovernmental Panel on Climate Change* (Cambridge Univ. Press, 2022).

32. J. S. Kikstra, Z. R. J. Nicholls, C. J. Smith, J. Lewis, R. D. Lamboll, E. Byers, M. Sandstad, M. Meinshausen, M. J. Gidden, J. Rogelj, E. Kriegler, G. P. Peters, J. S. Fuglestvedt, R. B. Skeie, B. H. Samset, L. Wienpahl, D. P. van Vuuren, K. I. van der Wijst, A. al Khourdajie, P. M. Forster, A. Reisinger, R. Schaeffer, K. Riahi, The IPCC Sixth Assessment Report WGIII climate assessment of mitigation pathways: From emissions to global temperatures. *Geosci. Model Dev.* **15**, 9075–9109 (2022).

33. A. Gelman, J. B. Carlin, H. S. Stern, D. B. Rubin, *Bayesian Data Analysis* (Chapman and Hall/CRC, 1995).

34. N. Cressie, C. K. Wikle, *Statistics for Spatio-Temporal Data* (John Wiley & Sons, 2015).

35. C. G. Piecuch, P. Huybers, C. C. Hay, A. C. Kemp, C. M. Little, J. X. Mitrovica, R. M. Ponte, M. P. Tingley, Origin of spatial variation in US East Coast sea-level trends during 1900-2017. *Nature* **564**, 400–404 (2018).

36. F. M. Calafat, T. Frederikse, K. Horsburgh, The sources of sea-level changes in the Mediterranean sea since 1960. *J. Geophys. Res. C: Oceans* **127**, e2022JC019061 (2022).

37. R. E. Kopp, A. C. Kemp, K. Bittermann, B. P. Horton, J. P. Donnelly, W. R. Gehrels, C. C. Hay, J. X. Mitrovica, E. D. Morrow, S. Rahmstorf, Temperature-driven global sea-level variability in the Common Era. *Proc. Natl. Acad. Sci. U.S.A.* **113**, E1434–E1441 (2016).

38. E. L. Ashe, N. Cahill, C. Hay, N. S. Khan, A. Kemp, S. E. Engelhart, B. P. Horton, A. C. Parnell, R. E. Kopp, Statistical modeling of rates and trends in Holocene relative sea level. *Quat. Sci. Rev.* **204**, 58–77 (2019).

39. A. M. R. Bakker, T. E. Wong, K. L. Ruckert, K. Keller, Sea-level projections representing the deeply uncertain contribution of the West Antarctic ice sheet. *Sci. Rep.* **7**, 3880 (2017).

40. T. E. Wong, A. M. R. Bakker, K. Ruckert, P. Applegate, A. B. A. Slangen, K. Keller, BRICK v0.2, a simple, accessible, and transparent model framework for climate and regional sea-level projections. *Geosci. Model Dev.* **10**, 2741–2760 (2017).

41. S. J. Holgate, A. Matthews, P. L. Woodworth, L. J. Rickards, M. E. Tamisiea, E. Bradshaw, P. R. Foden, K. M. Gordon, S. Jevrejeva, J. Pugh, New data systems and products at the Permanent Service for Mean Sea Level. *J. Coast. Res.* **29**, 493–504 (2013).

42. Permanent Service for Mean Sea Level, Obtaining Tide Gauge Data (PSMSL, 2023); www.psmsl.org/data/obtaining/.

43. W. C. Hammond, G. Blewitt, C. Kreemer, R. S. Nerem, GPS imaging of global vertical land motion for studies of sea level rise. *J. Geophys. Res. Solid Earth.* **126**, e2021JB022355 (2021).

44. Copernicus Climate Change Service, Sea level gridded data from satellite observations for the global ocean from 1993 to present, Climate Data Store (2018).

45. M. Mengel, A. Levermann, K. Frieler, A. Robinson, B. Marzeion, R. Winkelmann, Future sea level rise constrained by observations and long-term commitment. *Proc. Natl. Acad. Sci. U.S.A.* **113**, 2597–2602 (2016).

46. T. H. J. Hermans, J. M. Gregory, M. D. Palmer, M. A. Ringer, C. A. Katsman, A. B. A. Slangen, Projecting global mean sea-level change using CMIP6 models. *Geophys. Res. Lett.* **48**, e2020GL092064 (2021).

47. T. Bossy, T. Gasser, P. Ciais, Pathfinder v1.0.1: A Bayesian-inferred simple carbon-climate model to explore climate change scenarios. *Geosci. Model Dev.* **15**, 8831–8868 (2022).

48. M. Perrette, F. Landerer, R. Riva, K. Frieler, M. Meinshausen, A scaling approach to project regional sea level rise and its uncertainties. *Earth Syst. Dynam.* **4**, 11–29 (2013).

49. C. C. Hay, E. Morrow, R. E. Kopp, J. X. Mitrovica, Probabilistic reanalysis of twentieth-century sea-level rise. *Nature* **517**, 481–484 (2015).

50. J. Yin, M. E. Schlesinger, R. J. Stouffer, Model projections of rapid sea-level rise on the northeast coast of the United States. *Nat. Geosci.* **2**, 262–266 (2009).

51. J.-H. Malles, B. Marzeion, Twentieth century global glacier mass change: An ensemble-based model reconstruction. *Cryosphere* **15**, 3135–3157 (2021).

52. B. Marzeion, R. Hock, B. Anderson, A. Bliss, N. Champollion, K. Fujita, M. Huss, W. W. Immerzeel, P. Kraaijenbrink, J. H. Malles, F. Maussion, V. Radić, D. R. Rounce, A. Sakai, S. Shannon, R. van de Wal, H. Zekollari, Partitioning the uncertainty of ensemble projections of global glacier mass change. *Earths Future* **8**, e2019EF001470 (2020).

53. P. Prandi, B. Meyssignac, M. Ablain, G. Spada, A. Ribes, J. Benveniste, Local sea level trends, accelerations and uncertainties over 1993-2019. *Sci Data* **8**, 1 (2021).

54. V. Eyring, S. Bony, G. A. Meehl, C. A. Senior, B. Stevens, R. J. Stouffer, K. E. Taylor, Overview of the Coupled Model Intercomparison Project Phase 6 (CMIP6) experimental design and organization. *Geosci. Model Dev.* **9**, 1937–1958 (2016).

55. O. Abril-Pla, V. Andreani, C. Carroll, L. Dong, C. J. Fonnesbeck, M. Kochurov, R. Kumar, J. Lao, C. C. Luhmann, O. A. Martin, M. Osthege, R. Vieira, T. Wiecki, R. Zinkov, PyMC: A modern, and comprehensive probabilistic programming framework in Python. *PeerJ Comput. Sci.* **9**, e1516 (2023).

56. M. Zemp, M. Huss, E. Thibert, N. Eckert, R. McNabb, J. Huber, M. Barandun, H. Machguth, S. U. Nussbaumer, I. Gärtner-Roer, L. Thomson, F. Paul, F. Maussion, S. Kutuzov, J. G. Cogley, Global glacier mass changes and their contributions to sea-level rise from 1961 to 2016. *Nature* **568**, 382–386 (2019).

57. Z. Nicholls, M. Meinshausen, J. Lewis, AR6 WG3 Plots and Processing (2022); https://zenodo.org/records/6496232.

58. United Nations Framework Convention on Climate Change, The Paris Agreement (UNFCCC, 2015); http://unfccc.int/paris_agreement/items/9485.php.

59. A. S. Kolker, M. A. Allison, S. Hameed, An evaluation of subsidence rates and sea-level variability in the northern Gulf of Mexico. *Geophys. Res. Lett.* **38**, L21404 (2011).

60. A. Boretti, A revised procedure to analyze the time series of monthly average mean sea levels corrected for non-linear subsidence. *Arab. J. Geosci.* **15**, 1667 (2022).

61. T. Frederikse, S. Adhikari, T. J. Daley, S. Dangendorf, R. Gehrels, F. Landerer, M. Marcos, T. L. Newton, G. Rush, A. B. A. Slangen, G. Wöppelmann, Constraining 20th-century sea-level rise in the south Atlantic ocean. *J. Geophys. Res. Oceans* **126**, e2020JC016970 (2021).

62. C. G. Piecuch, River effects on sea-level rise in the Río de la Plata estuary during the past century. *Ocean Sci.* **19**, 57–75 (2023).

63. J. Oelsmann, M. Marcos, M. Passaro, L. Sanchez, D. Dettmering, S. Dangendorf, F. Seitz, Regional variations in relative sea-level changes influenced by nonlinear vertical land motion. *Nat. Geosci.* **17**, 137–144 (2024).

64. G. G. Garner, T. Hermans, R. E. Kopp, A. B. A. Slangen, T. L. Edwards, A. Levermann, S. Nowikci, M. D. Palmer, C. Smith, B. Fox-Kemper, H. T. Hewitt, C. Xiao, G. Aðalgeirsdóttir, S. S. Drijfhout, T. L. Edwards, N. R. Golledge, M. Hemer, G. Krinner, A. Mix, D. Notz, S. Nowicki, I. S. Nurhati, L. Ruiz, J-B. Sallée, Y. Yu, L. Hua, T. Palmer, B. Pearson, *IPCC AR6 Sea Level Projections* (2021).

65. J. Wang, X. Zhang, J. A. Church, M. King, X. Chen, Near-term future sea-level projections supported by extrapolation of tide-gauge observations. *Geophys. Res. Lett.* **52**, e2024GL112940 (2025).

66. M. Carson, K. Lyu, K. Richter, M. Becker, C. M. Domingues, W. Han, L. Zanna, Climate model uncertainty and trend detection in regional sea level projections: A review. *Surv. Geophys.* **40**, 1631–1653 (2019).

67. G. Erkens, T. Bucx, R. Dam, G. de Lange, J. Lambert, "Sinking coastal cities," in *Prevention and Mitigation of Natural and Anthropogenic Hazards due to Land Subsidence - Ninth International Symposium on Land Subsidence (NISOLS)*, Nagoya, Japan, 15 to 19 November 2015 (Copernicus GmbH, 2015), vol. 372, pp. 189–198.

68. C. F. Larsen, K. A. Echelmeyer, J. T. Freymueller, R. J. Motyka, Tide gauge records of uplift along the northern Pacific-North American plate boundary, 1937 to 2001. *J. Geophys. Res.* **108**, 2216 (2003).

69. J. A. Lowe, J. M. Gregory, A sea of uncertainty. *Nat. Clim. Change* **1**, 42–43 (2010).

70. S. Rahmstorf, A semi-empirical approach to projecting future sea-level rise. *Science* **315**, 368–370 (2007).

71. F. Pattyn, M. Morlighem, The uncertain future of the Antarctic Ice Sheet. *Science* **367**, 1331–1335 (2020).

72. R. E. Kopp, R. M. DeConto, D. A. Bader, C. C. Hay, R. M. Horton, S. Kulp, M. Oppenheimer, D. Pollard, B. H. Strauss, Evolving understanding of Antarctic ice-sheet physics and ambiguity in probabilistic sea-level projections. *Earths Future* **5**, 1217–1233 (2017).

73. A. Aschwanden, T. C. Bartholomaus, D. J. Brinkerhoff, M. Truffer, Brief communication: A roadmap towards credible projections of ice sheet contribution to sea level. *Cryosphere* **15**, 5705–5715 (2021).

74. I. N. Otosaka, A. Shepherd, E. R. Ivins, N. J. Schlegel, C. Amory, M. R. van den Broeke, M. Horwath, I. Joughin, M. D. King, G. Krinner, S. Nowicki, A. J. Payne, E. Rignot, T. Scambos, K. M. Simon, B. E. Smith, L. S. Sørensen, I. Velicogna, P. L. Whitehouse, G. A, C. Agosta, A. P. Ahlstrøm, A. Blazquez, W. Colgan, M. E. Engdahl, X. Fettweis, R. Forsberg, H. Gallée, A. Gardner, L. Gilbert, N. Gourmelen, A. Groh, B. C. Gunter, C. Harig, V. Helm, S. A. Khan, C. Kittel, H. Konrad, P. L. Langen, B. S. Lecavalier, C. C. Liang, B. D. Loomis, M. McMillan, D. Melini, S. H. Mernild, R. Mottram, J. Mouginot, J. Nilsson, B. Noël, M. E. Pattle, W. R. Peltier, N. Pie, M. Roca, I. Sasgen, H. V. Save, K. W. Seo, B. Scheuchl, E. J. O. Schrama, L. Schröder, S. B. Simonsen, T. Slater, G. Spada, T. C. Sutterley, B. D. Vishwakarma, J. M. van Wessem, D. Wiese, W. van der Wal, B. Wouters, Mass balance of the Greenland and Antarctic ice sheets from 1992 to 2020. *Earth Syst. Sci. Data* **15**, 1597–1616 (2023).

75.J A. Church, P. U. Clark, A. Cazenave, J. M. Gregory, S. Jevrejeva, A. Levermann, M. A. Merrifield, G. A. Milne, R. S. Nerem, P. D. Nunn, A. J. Payne, W. T. Pfeffer, D. Stammer, A. S. Unnikrishnan, Sea Level Change (Chapter 13), in *Climate Change 2013: The Physical Science Basis.*, Contribution of Working Group I to the Fifth Assessment Report of the Intergovernmental Panel on Climate Change, (Cambridge Univ. Press, Cambridge, United Kingdom and New York, NY, USA 2013), pp. 1137–1216.

76. C. R. Stokes, N. J. Abram, M. J. Bentley, T. L. Edwards, M. H. England, A. Foppert, S. S. R. Jamieson, R. S. Jones, M. A. King, J. T. M. Lenaerts, B. Medley, B. W. J. Miles, G. J. G. Paxman, C. Ritz, T. van de Flierdt, P. L. Whitehouse, Response of the East Antarctic Ice Sheet to past and future climate change. *Nature* **608**, 275–286 (2022).

77. O. Geoffroy, D. Saint-Martin, D. J. L. Olivié, A. Voldoire, G. Bellon, S. Tytéca, Transient climate response in a two-layer energy-balance model. Part I: Analytical solution and parameter calibration using CMIP5 AOGCM experiments. *J. Clim.* **26**, 1841–1857 (2013).

78. C. J. Smith, P. M. Forster, M. Allen, N. Leach, R. J. Millar, G. A. Passerello, L. A. Regayre, FAIR v1.3: A simple emissions-based impulse response and carbon cycle model. *Geosci. Model Dev.* **11**, 2273–2297 (2018).

79. E. Lambert, D. Le Bars, H. Goelzer, R. S. W. van de Wal, Correlations between sea-level components are driven by regional climate change. *Earths Future* **9**, e2020EF001825 (2021).

80. R. Hawkins, L. Husson, G. Choblet, T. Bodin, J. Pfeffer, Virtual tide gauges for predicting relative sea level rise. *J. Geophys. Res. Solid Earth.* **124**, 13367–13391 (2019).

81. B. H. Strauss, P. M. Orton, K. Bittermann, M. K. Buchanan, D. M. Gilford, R. E. Kopp, S. Kulp, C. Massey, H. Moel, S. Vinogradov, Economic damages from Hurricane Sandy attributable to sea level rise caused by anthropogenic climate change. *Nat. Commun.* **12**, 2720 (2021).

82. RGI Consortium, Randolph Glacier Inventory - A dataset of global glacier outlines, version 7 (2023).

83. T. M. L. Wigley, S. C. B. Raper, Extended scenarios for glacier melt due to anthropogenic forcing. *Geophys. Res. Lett.* **32**, L05704 (2005).

84. D. Parkes, B. Marzeion, Twentieth-century contribution to sea-level rise from uncharted glaciers. *Nature* **563**, 551–554 (2018).

85. D. Farinotti, M. Huss, J. J. Fürst, J. Landmann, H. Machguth, F. Maussion, A. Pandit, A consensus estimate for the ice thickness distribution of all glaciers on Earth. *Nat. Geosci.* **12**, 168–173 (2019).

86. R. Hugonnet, R. McNabb, E. Berthier, B. Menounos, C. Nuth, L. Girod, D. Farinotti, M. Huss, I. Dussaillant, F. Brun, A. Kääb, Accelerated global glacier mass loss in the early twenty-first century. *Nature* **592**, 726–731 (2021).

87. T. Frederikse, F. Landerer, L. Caron, S. Adhikari, D. Parkes, V. W. Humphrey, S. Dangendorf, P. Hogarth, L. Zanna, L. Cheng, Y.-H. Wu, Data supplement of "The causes of sea-level rise since 1900" (2020); https://doi.org/10.5281/zenodo.3862995.

88. M. Vermeer, S. Rahmstorf, Global sea level linked to global temperature. *Proc. Natl. Acad. Sci. U.S.A.* **106**, 21527–21532 (2009).

89. G. G. Garner, R. E. Kopp, Framework for Assessing Changes To Sea-level (FACTS) modules, scripts, and data for the IPCC AR6 sea level projections (2022); https://zenodo.org/records/6419954.

90. L. Caron, E. R. Ivins, E. Larour, S. Adhikari, J. Nilsson, G. Blewitt, GIA model statistics for GRACE hydrology, cryosphere, and ocean science. *Geophys. Res. Lett.* **45**, 2203–2212 (2018).

91. IPCC, Data for the Working Group I (WGI) Contribution to the Intergovernmental Panel on Climate Change (IPCC) Sixth Assessment Report (AR6) (2021); https://catalogue.ceda.ac.uk/uuid/3234e9111d4f4354af00c3aaecd879b7.

92. G. Le Cozannet, J.-C. Manceau, J. Rohmer, Bounding probabilistic sea-level projections within the framework of the possibility theory. *Environ. Res. Lett.* **12**, 014012 (2017).

93. R. E. Kopp, M. Oppenheimer, J. L. O'Reilly, S. S. Drijfhout, T. L. Edwards, B. Fox-Kemper, G. G. Garner, N. R. Golledge, T. H. J. Hermans, H. T. Hewitt, B. P. Horton, G. Krinner, D. Notz, S. Nowicki, M. D. Palmer, A. B. A. Slangen, C. Xiao, Communicating future sea-level rise uncertainty and ambiguity to assessment users. *Nat. Clim. Change* **13**, 648–660 (2023).

94. R. M. Ponte, Low-frequency sea level variability and the inverted barometer effect. *J. Atmos. Oceanic Tech.* **23**, 619–629 (2006).

95. G. Taburet, A. Sanchez-Roman, M. Ballarotta, M.-I. Pujol, J.-F. Legeais, F. Fournier, Y. Faugere, G. Dibarboure, DUACS DT2018: 25 years of reprocessed sea level altimetry products. *Ocean Sci.* **15**, 1207–1224 (2019).

96. K. Gobron, O. de Viron, G. Wöppelmann, É. Poirier, V. Ballu, M. van Camp, Assessment of tide gauge biases and precision by the combination of multiple collocated time series. *J. Atmos. Oceanic Tech.* **36**, 1983–1996 (2019).

97. C. Tay, E. O. Lindsey, S. T. Chin, J. W. McCaughey, D. Bekaert, M. Nguyen, H. Hua, G. Manipon, M. Karim, B. P. Horton, T. Li, E. M. Hill, Sea-level rise from land subsidence in major coastal cities. *Nat. Sustain.* **5**, 1049–1057 (2022).

98. G. Blewitt, C. Kreemer, W. C. Hammond, J. Gazeaux, MIDAS robust trend estimator for accurate GPS station velocities without step detection. *J. Geophys. Res. Solid Earth.* **121**, 2054–2068 (2016).

99. M. D. Hoffman, A. Gelman, The No-U-Turn Sampler: Adaptively setting path lengths in Hamiltonian Monte Carlo. *J. Mach. Learn. Res.* **15**, 1593–1623 (2014).

100. P. R. Thompson, M. A. Merrifield, A unique asymmetry in the pattern of recent sea level change. *Geophys. Res. Lett.* **41**, 7675–7683 (2014).

101. T. Frederikse, T. Gerkema, Multi-decadal variability in seasonal mean sea level along the North Sea coast. *Ocean Sci.* **14**, 1491–1501 (2018).

102. R. E. Kopp, Framework for Assessing Changes To Sea-level (FACTS) module data (2022). [Data set]. Zenodo. https://doi.org/10.5281/zenodo.7478192.
